# Engineering Robust Metallic Zero-Mode States in Olympicene Graphene Nanoribbons

**DOI:** 10.1021/jacs.3c01576

**Published:** 2023-07-10

**Authors:** Ryan D. McCurdy, Aidan Delgado, Jingwei Jiang, Junmian Zhu, Ethan Chi Ho Wen, Raymond E. Blackwell, Gregory C. Veber, Shenkai Wang, Steven G. Louie, Felix R. Fischer

**Affiliations:** † Department of Chemistry, University of California, Berkeley, California 94720, United States; ‡ Department of Physics, University of California, Berkeley, California 94720, United States; § Materials Sciences Division, Lawrence Berkeley National Laboratory, Berkeley, California 94720, United States; ∥ Kavli Energy NanoSciences Institute at the University of California Berkeley and the Lawrence Berkeley National Laboratory, Berkeley, California 94720, United States; ⊥ Bakar Institute of Digital Materials for the Planet, Division of Computing, Data Science, and Society, University of California, Berkeley, California 94720, United States

## Abstract

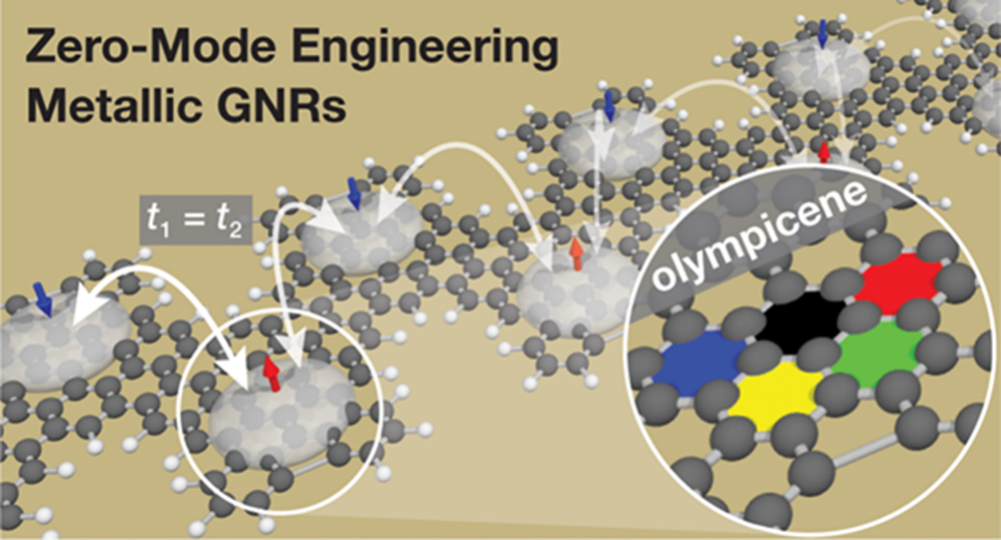

Metallic graphene nanoribbons (GNRs) represent a critical component in the toolbox of low-dimensional functional materials technology serving as 1D interconnects capable of both electronic and quantum information transport. The structural constraints imposed by on-surface bottom-up GNR synthesis protocols along with the limited control over orientation and sequence of asymmetric monomer building blocks during the radical step-growth polymerization have plagued the design and assembly of metallic GNRs. Here, we report the regioregular synthesis of GNRs hosting robust metallic states by embedding a symmetric zero-mode (ZM) superlattice along the backbone of a GNR. Tight-binding electronic structure models predict a strong nearest-neighbor electron hopping interaction between adjacent ZM states, resulting in a dispersive metallic band. First-principles density functional theory-local density approximation calculations confirm this prediction, and the robust, metallic ZM band of olympicene GNRs is experimentally corroborated by scanning tunneling spectroscopy.

## Introduction

Graphene nanoribbons (GNRs) are representatives of an emerging class of bottom-up synthesized designer quantum materials whose electronic structure can be tuned with atomic precision by deterministic chemical designs. Their structures exhibit unusual and some never before realized physical properties that extend far beyond the parent 2D graphene. Highly tunable band gaps,^1−3^ photoemission,^4^ magnetic spin chains,^5^ and even symmetry-protected topological states^6−9^ can all be tailored by real space structural parameters including, among others, width, symmetry, edge termination, and substitutional doping.^10−13^ A dominant electronic feature common to almost all GNRs is the opening of a sizeable band gap imposed by laterally confining a 2D graphene sheet to a quasi-1D GNR (width < 2 nm). This quantum confinement effect has emerged as a veritable challenge to the design of intrinsically metallic band structures. Bottom-up access to a family of robust metallic GNRs not only represents a critical component in the development of advanced nanographene-based logic circuits,^14^ e.g., as covalent interconnects capable of electronic and quantum transport, but could serve a versatile and highly tunable platform to explore emergent physical phenomena such as Luttinger liquids,^15−18^ plasmonics,^19−22^ charge density waves,^23−26^ and superconductivity in 1D.^27−30^

**Figure 1:**
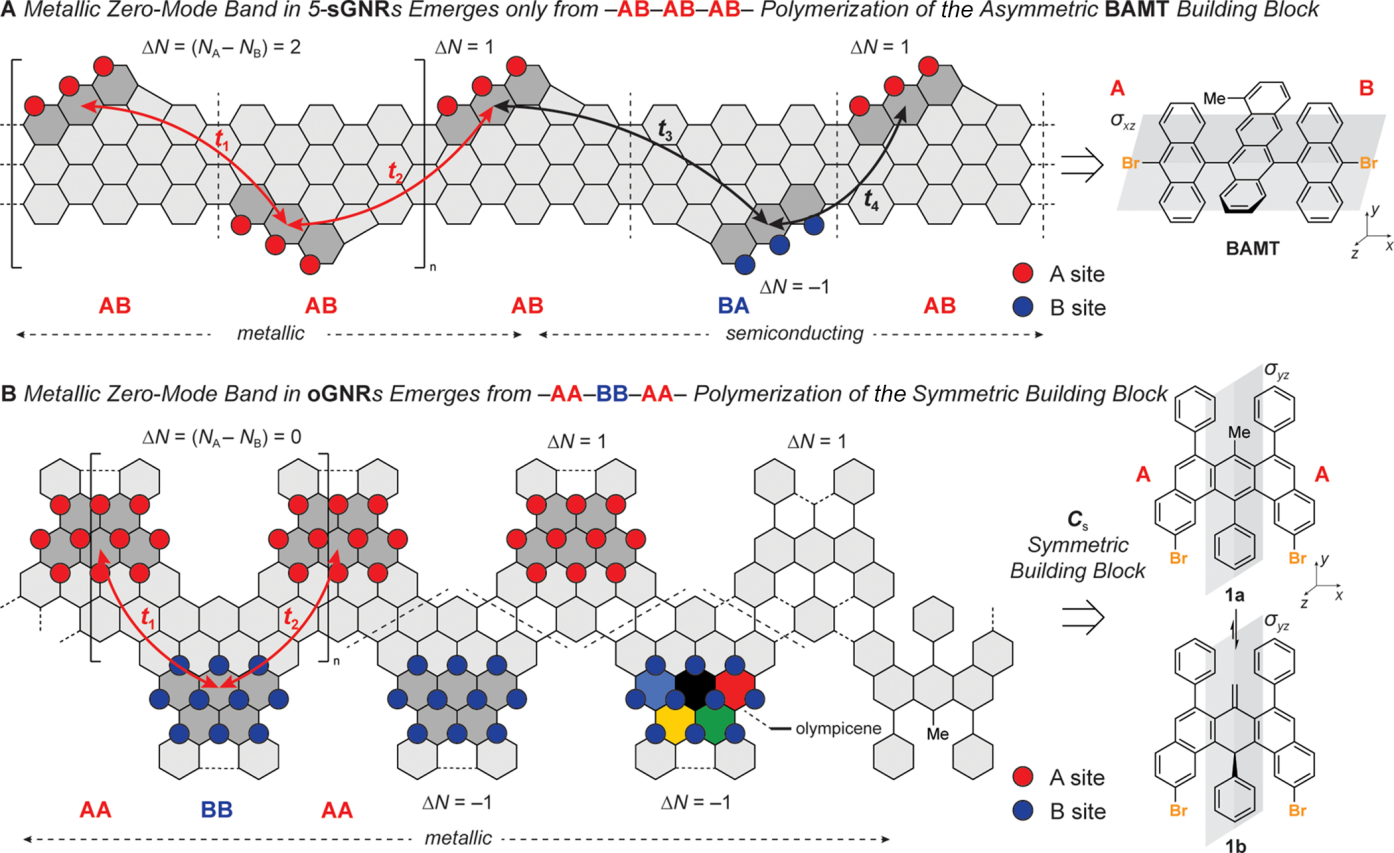
Bottom-up design and ZM engineering of metallic GNRs. (A) Metallic band in 5-sGNRs emerges only if the orientation of the monomers along the axis of polymerization (*x*-axis) follows a regioregular −AB–AB–AB– pattern. (B) Presence of a σ_*yz*_ mirror plane in **1a**/**b** normal to the axis of polymerization (*x*-axis) ensures that either of two possible orientations of a monomer during the radical step-growth polymerization gives rise to a metallic ZM band in oGNRs.

We recently reported a general approach for accessing metallic GNRs by embedding a superlattice of localized zero-mode (ZM) states along the backbone of a bottom-up synthesized sawtooth GNR (sGNR).^31,32^ A key ingredient to this approach was the design of a molecular building block, 6,11-bis(10-bromoanthracen-9-yl)-1-methyltetracene (BAMT in Figure 1), that introduces a sublattice imbalance (Δ*N* = *N*_A_ – *N*_B_) between carbon atoms occupying the A and the B sublattice sites of graphene, respectively. The concept is reminiscent of Lieb’s theorem,^33^ a surplus of carbon atoms on sublattice A versus sublattice B will lead to Δ*N* eigenstates at *E* = 0 eV, or ZMs, localized on the majority sublattice. Application of a simple tight binding model, the Su–Schrieffer–Heeger (SSH) dispersion relationship,^34^ that describes the interaction between these local ZM states gave rise to two distinctive bands defined by an intracell hopping amplitude *t*_1_ and an intercell hopping amplitude *t*_2_. The energy gap enclosed by these bands is Δ*E* = 2||*t*_1_| – |*t*_2_||. If the absolute magnitude of the two hopping amplitudes is equal, i.e., |*t*_1_| = |*t*_2_|, as illustrated for the evenly spaced ZM states in sGNR (Figure 1A), the energy gap vanishes and the 1D electronic structure becomes metallic.^35,36^ The presence of a metallic ZM band at the Fermi level (*E*_F_) in sGNRs could be visualized by scanning tunneling spectroscopy (STS) and was further corroborated by density functional theory-local density approximation (DFT-LDA) calculations. This method, however, suffered from a Stoner-type instability for narrow bands that could open up a spin-splitting gap. To overcome this, we had to introduce an effective sublattice mixing (e.g., introduction of 5-membered rings in 5-sGNRs) to facilitate the hopping between the localized zero modes.

A major shortcoming inherent to the design of 5-sGNRs is the requirement that all bonds formed between molecular precursors as part of the on-surface radical step-growth polymerization have to follow a strict head-to-tail pattern (−AB–AB–AB– in Figure 1A) to ensure that the intracell hopping amplitude |*t*_1_| remains equal in magnitude to the intercell hopping amplitude |*t*_2_|. The statistical probability that this specific arrangement is adopted for a single C–C bond-forming step on the surface is only ∼50%. Were the molecular building blocks to fuse in the undesirable head-to-head (−BA–AB−) or tail-to-tail (−AB–BA−) configuration, the ZM bands would split (|*t*_3_| ≠ |*t*_4_|) and give rise to a semiconductor rather than a metal.^31,32^ The probability of producing a metallic sGNR segment from *n* monomers is therefore *P_n_* = (0.5)^*n*^ or less than 1% for *n* > 7, severely limiting the use of metallic sGNRs at length scales necessary for applications as device interconnects. While sGNRs served as a successful proof of concept for our general approach to access metallic phases in GNRs, designs that ensure regioregularity and an efficient sublattice mixing of ZM states are needed to obtain uniform samples of extended GNRs with persistent, intrinsically metallic ZM bands.

Here, we report the design and on-surface synthesis of metallic olympicene GNRs (oGNRs) derived from *C*_s_ symmetric molecular building blocks **1a**,**b** (Figure 1B) (herein, **1a** and **1b** represent two discrete constitutional isomers that interconvert through a tautomerization equilibrium). Rather than relying on a statistical distribution of bond-forming events that dictated the band structure in sGNRs, the molecular building blocks for oGNRs feature a σ_*yz*_ mirror plane perpendicular to the *x*-axis, the main axis of polymerization, ensuring that oGNRs arising from **1a**,**b** will always be metallic. This could be achieved by placing the carbon atom contributing to the sublattice imbalance Δ*N*, the methyl group in **1a** or the methylene in **1b**, along the σ_*yz*_ mirror plane of the building block. The arrangement of any two monomers forming the oGNR unit cell ensures that the position of the ZM state alternates between the A and the B sublattice sites. The efficient sublattice mixing that gives rise to a robust metallic ZM band is built into the design. Atomically precise oGNRs were synthesized from molecular precursors on a Au(111) surface and characterized in ultrahigh-vacuum (UHV) by low-temperature scanning tunneling microscopy (STM) and STS. Experimental results are further corroborated by first-principles calculations, revealing a robust metallic band that spans across *E*_F_ emerging from the interaction of ZM states along the backbone of oGNRs.

## Results and Discussion

### Synthesis of Molecular Precursors for oGNRs

**Figure 2:**
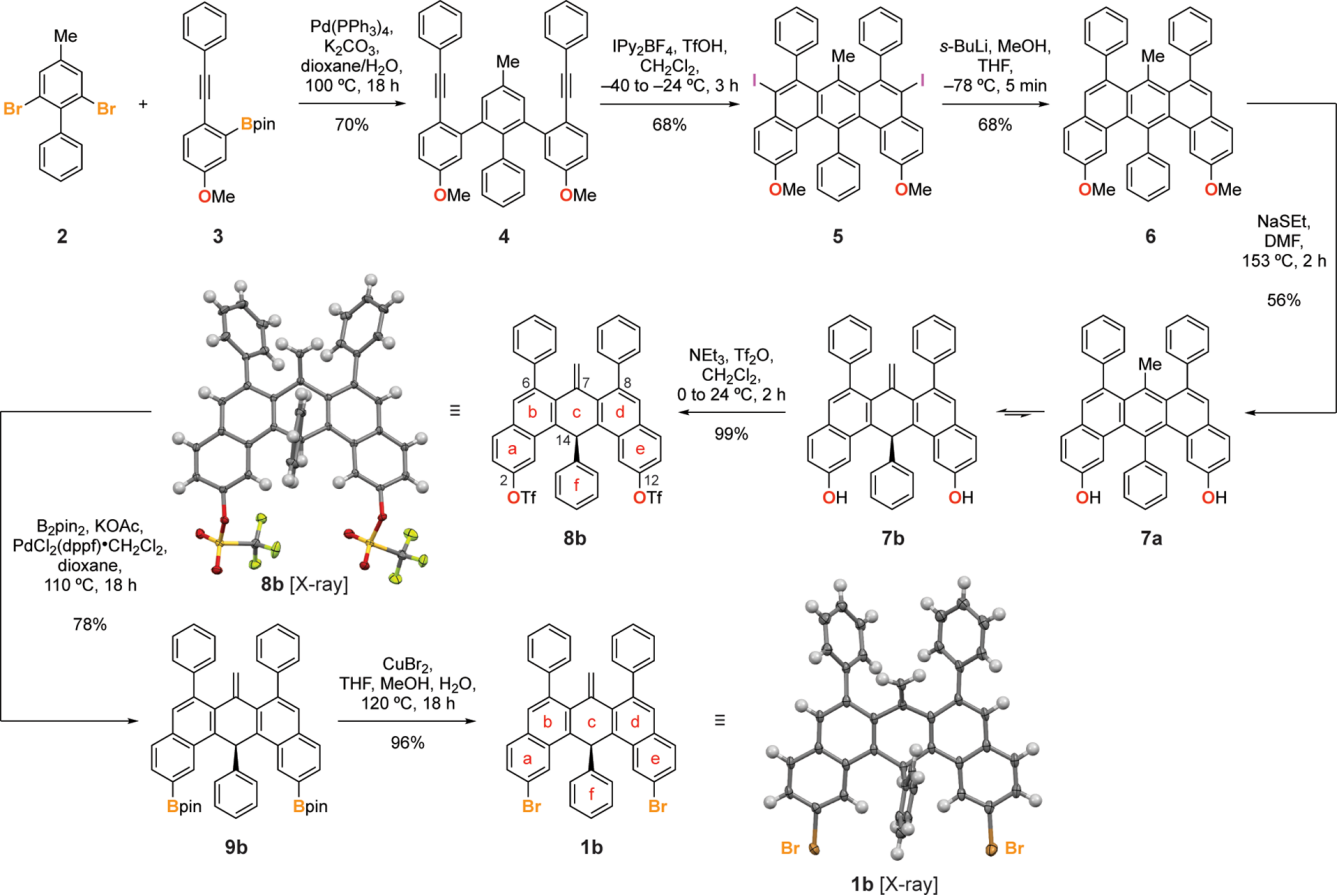
Synthesis of molecular precursor **1b** for oGNRs. Single X-ray crystal structures of **8b** and **1b**. Thermal ellipsoids are drawn at the 50% probability level. Color coding: C (gray), O (red), F (green), S (yellow), Br (orange). Hydrogen atoms are placed at calculated positions.

The synthesis of the molecular precursor **1b** for oGNRs is depicted in Figure 2. Double Suzuki cross-coupling of 2,6-dibromo-4-methyl-1,1′-biphenyl (**2**) with 2 equiv of 2-(5-methoxy-2-(phenylethynyl)phenyl)-4,4,5,5-tetramethyl-1,3,2-dioxaborolane (**3**) yielded the diyne **4**. Treatment of **4** with Barluenga’s reagent in TfOH successfully induced the sterically demanding benzannulation to give the benzo[*m*]tetraphene core **5**. The two aryl iodide groups in **5** were removed by lithium-halogen exchange with *s*-BuLi followed by protonation with MeOH to yield **6**. With the assembly of the characteristic carbon backbone of the monomer building block completed, the task shifted to converting the methoxy groups in **6** to aryl halides that serve as thermally labile chemical handles during the on-surface GNR growth. A well-precedented route involves deprotection of aryl-methyl ethers to reveal the free alcohols followed by conversion into aryltriflates which serve as versatile handles for further diversification.^11^^1^H and ^13^C NMR revealed that deprotection of **6** under Lewis/Brønsted acidic (e.g., BBr_3_, AlBr_3_, TMSI, HBr, HI, and TfOH) or nucleophilic (e.g., NaSEt and LiI) conditions induced a tautomerization of the benzo[*m*]tetraphene core to yield predominantly the 7-methylene-7,14-dihydrobenzo[*m*]tetraphene **7b** rather than the tautomeric species **7a**. Following the synthetic route outlined above, treatment of **7b** with Tf_2_O gave access to the triflate **8b**. Single crystals suitable for X-ray diffraction were grown by slow diffusion of MeOH into a saturated solution of **8b** in CH_2_Cl_2_. The crystal structure of **8b** revealed that the central ring of the dihydrobenzo[*m*]tetraphene core, ring **c** in Figure 2, adopts a boat-like conformation placing the methylene group at C7 and the phenyl group at C14 at angles of 35.0° and 76.0° above the base plane spanned by the remaining four carbon atoms (C6a, C7a, C13b, and C14a) of ring **c**, respectively. While this conformation comes at the cost of breaking the extended aromatic ring system of a benzo[*m*]tetraphene core into two isolated naphthalene units, the boat conformation adopted by ring **c** significantly reduces the A^1,3^ strain between the exocyclic methylene group and the two phenyl substituents at C6 and C8. To complete the synthesis, the triflates in **8b** were converted into the diboronic ester **9b** before treatment with excess CuBr_2_ yielded the 2,12-dibromo-7,14-dihydrobenzo[*m*]tetraphene **1b**, the molecular building block for oGNRs. Single crystals of **1b** suitable for X-ray diffraction and surface-assisted oGNR growth were obtained by diffusion of MeOH into a saturated solution of **1b** in CH_2_Cl_2_. In close analogy to the conformation adopted by **8b**, the ring **c** in dihydrobenzo[*m*]tetraphene **1b** adopts a boat-like conformation. The included angles between the methylene group at C7 and the phenyl substituent at C14 with the base plane of ring **c** are 37.5° and 75.0°, respectively.

### Surface-Assisted Growth and Electronic Structure Characterization of oGNRs

**Figure 3:**
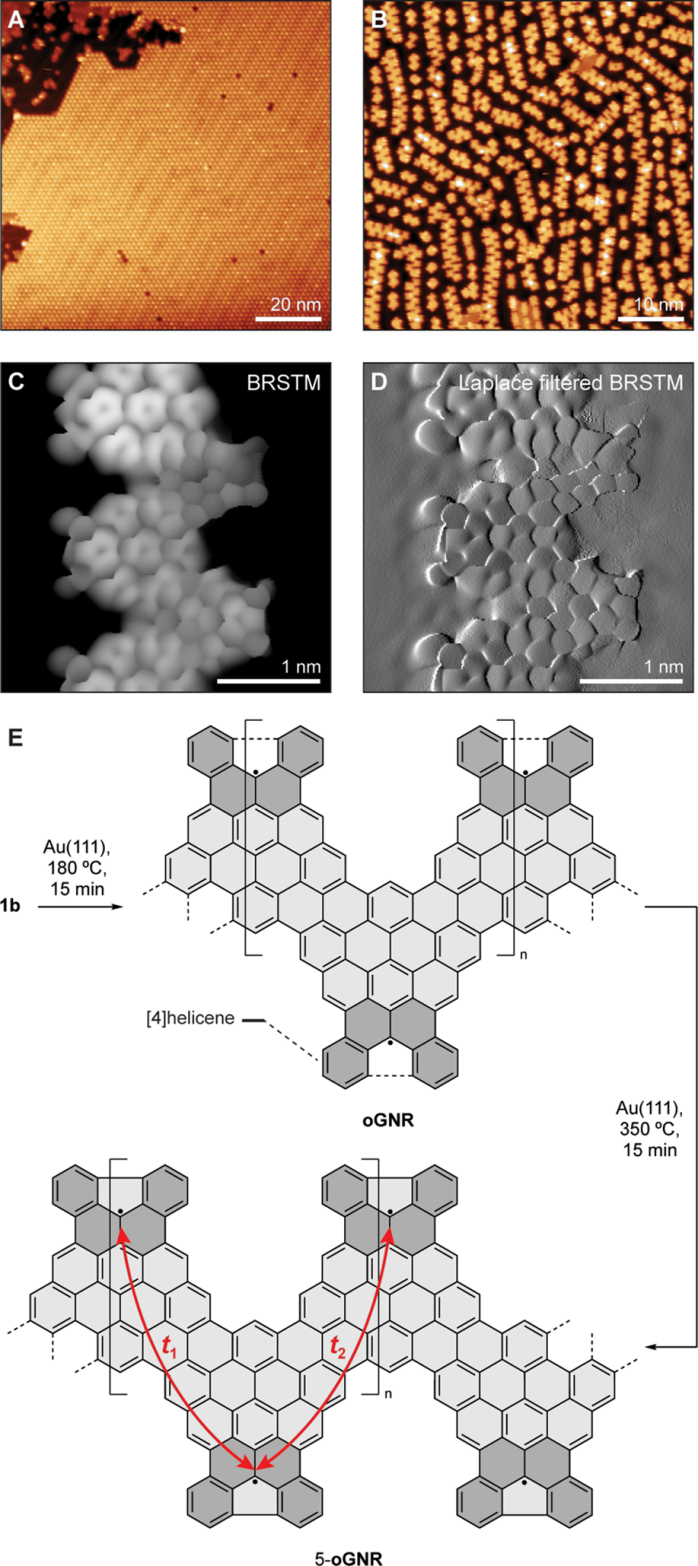
Bottom-up synthesis of 5-oGNRs. (A) STM topographic image of a self-assembled island of molecular precursor **1b** on Au(111) (*V*_s_ = 0.05 V, *I*_t_ = 20 pA). (B) STM topographic image of a high coverage sample of 5-oGNRs following annealing to 350 °C (*V*_s_ = 0.05 V, *I*_t_ = 20 pA). (C and D) BRSTM and Laplace-filtered BRSTM image of a 5-oGNR segment showing the 5-membered rings resulting from the fusion of [4]helicene groups along the oGNR edges (*V*_s_ = 0.01 V, *I*_t_ = 400 pA). (E) Schematic representation of the stepwise thermally induced cyclodehydrogenation that gives rise to 5-oGNRs.

Samples of metallic oGNRs were prepared following an established surface-assisted bottom-up GNR synthesis. Molecular precursor **1b** was sublimed in UHV from a Knudsen cell evaporator onto a Au(111) surface held at 25 °C. Figure 3A shows a representative topographic STM image of self-assembled islands of **1b** on an atomically flat Au(111) terrace. Step-growth polymerization of **1b** was induced by annealing the molecule-decorated surface first to 180 °C for 15 min followed by a second annealing step at 350 °C for 15 min to complete the cyclodehydrogenation. Topographic images of a high coverage sample, Figure 3B, reveal extended GNRs featuring a characteristic alternating pattern of protrusions along the backbone of the GNR and lengths ranging up to 30 nm (Supporting Information Figure S1). Bond-resolved STM (BRSTM) with CO-functionalized tips reveals that the radical step-growth polymerization proceeds concurrently with the partial cyclodehydrogenation of the oGNR backbone (Figure 3C,D). At 180 °C, the [4]-helicene fragments lining the edges of oGNRs have partially fused to form 5-membered rings (Figure 3E, Supporting Information Figure S1C). A second annealing step (350 °C for 15 min) merely completes the process, giving rise to a uniform edge termination in 5-oGNRs (Figure 3C,D, Supporting Information Figure S2).

**Figure 4:**
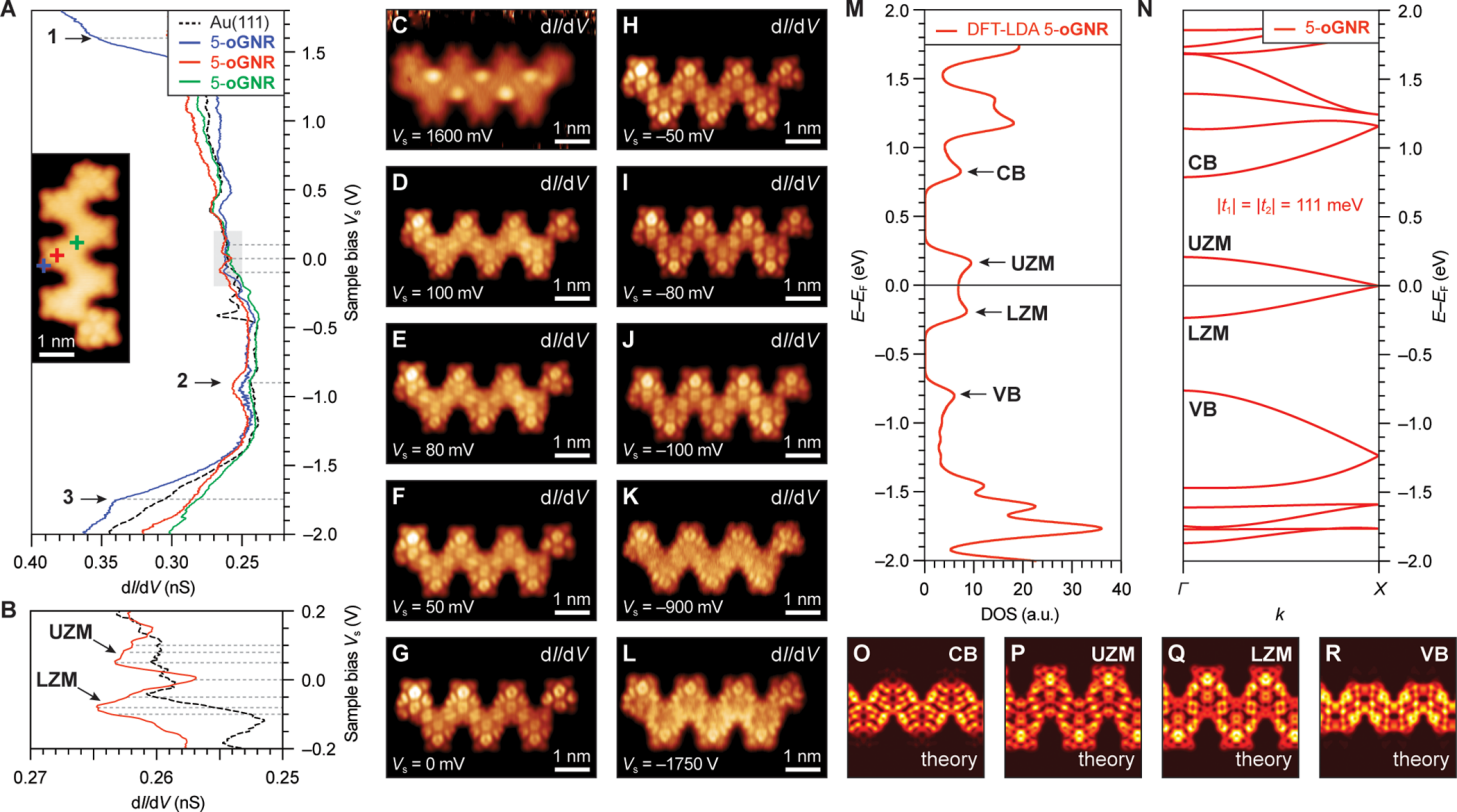
Electronic structure of 5-oGNRs. (A and B) STS d*I*/dV spectra recorded on a 5-oGNR at the positions marked in the inset STM topographic image with a red, blue, and green cross (spectroscopy: *V*_ac_ = 11 mV, *f* = 455 Hz; imaging: *V*_s_ = 50 mV, *I*_t_ = 20 pA, CO-functionalized tip). (C–L) Constant height d*I*/d*V* maps recorded at the indicated biases (spectroscopy: *V*_ac_ = 11 mV, *f* = 455 Hz). (M) DFT-LDA-calculated DOS of 5-oGNR (spectrum broadened by 10 meV Gaussian). Features associated with the CB, UZM, LZM, and VB are indicted by arrows. (N) DFT-LDA-calculated band structure of a freestanding 5-oGNR. A tight binding fit to the DFT-LDA band structure yields the hopping parameters |*t*_1_| = |*t*_2_| = 111 meV. (O–R) Calculated DFT-LDA LDOS maps evaluated at the edge of the bulk CB, at the UZM and LZM bands, and at the edge of the bulk VB.

Having resolved the chemical structure of 5-oGNRs, we shifted our focus to the characterization of its local electronic structure using differential tunneling spectroscopy. Figure 4A shows typical d*I*/d*V* point spectra for a 5-oGNR recorded with a CO-functionalized STM tip at the positions highlighted in the inset. Three spectral features can clearly be seen in the range of −2.00 V < *V*_s_ < +1.80 V. Two shoulders at *V*_s_ = +1.60 V (*Peak* 1) and *V*_s_ = −1.75 V (*Peak* 3) dominate the spectrum, along with a broad peak centered at *V*_s_ = −0.90 V (*Peak* 2). The signal intensities of *Peaks* 1 and 3 are strongest when the STM tip is placed close to the convex protrusions lining the edge of the ribbon (blue line in Figure 4A), whereas *Peak* 2 is prominently featured in both spectra recorded above the center of an olympicene unit (red line in Figure 4A, Supporting Information Figure S3) and along the edge of the ribbon. Figure 4B shows d*I*/d*V* spectra taken over a narrower bias range −0.20 V < *V*_s_ < +0.20 V. Most prominent here is a U-shaped feature anchored by two peaks in the differential conductance spectrum at *V*_s_ = −0.08 V and *V*_s_ = +0.05 V when the STM tip is placed above the center of the olympicene unit. Differential conductance maps recorded over a bias range of *V*_s_ = +0.10 V to *V*_s_ = −0.10 V (Figure 4D–J, Supporting Information Figure S3H–P) show characteristic wavefunction patterns associated with two degenerate low-bias states that intersect at *V*_s_ = +0.00 V. The peak at *V*_s_ = −0.08 V can thus be assigned to the bottom edge of the lower ZM (LZM) of two ZM bands contributing to the metallic state in 5-oGNRs, while the peak at *V*_s_ = +0.05 V captures the top edge of the upper ZM (UZM) band. The U-shaped local density of states (LDOS) spanning across *E*_F_ is the signature of van Hove singularities associated with the flat band edges of the LZM and UZM bands.

### First-Principles Calculation of the 5-oGNR Electronic Structure

We further explored the metallic band structure of 5-oGNRs using ab initio DFT. Figure 4M,N shows the theoretical DOS and the band structure of a 5-oGNR calculated using an LDA to the exchange-correlation potential. Two highly dispersive bands, labeled LZM and UZM, span across the energy scale from *E*–*E*_F_ = −0.25 V to *E*–*E*_F_ = +0.25 V. The LZM and UZM bands cross *E*_F_ at ***k*** = *X*, giving rise to a robust metallic band with a width of *E*_ZM_ ∼ 0.5 eV. Both the upper and lower edges of the ZM bands show a flattening as they approach ***k*** = Γ. The corresponding calculated DOS (Figure 4M) faithfully reproduces the U-shaped signature of the metallic band identified in the experimental LDOS (Figure 4A,B). DFT-LDA LDOS projections evaluated at the energy of the UZM and LZM edges (Figure 4P,Q) show the characteristic nodal pattern observed in the corresponding d*I*/d*V* maps (Figure 4D–J). At energies above and below *E*–*E*_F_ = ±0.25 V, the calculated metallic ZMB gives way to minigaps, narrow regions of vanishing DOS, that span the energy window separating the ZMB from the bottom of the CB and the top of the VB, located at *E*–*E*_F_ = +0.80 V and *E*–*E*_F_ = −0.75 V, respectively. Both LZM and UZM bands can be fit to an SSH tight binding model
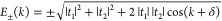
1with the intra- and intercell hopping amplitudes |*t*_1_| = |*t*_2_| = 111 meV and δ = 0 (δ is the relative phase between *t*_1_ and *t*_2_). Supercell calculations further show that the rigid GNR backbone renders oGNR virtually impervious to mechanical deformations usually associated with strong electron–phonon coupling along the main *x*-axis of the ribbon that would otherwise induce spontaneous metal–insulator transitions (i.e., Peierls distortion). A decisive advantage of oGNRs over the first-generation metallic sGNRs is that the *C*_s_ symmetric molecular precursor **1b** features a σ_*yz*_ mirror plane perpendicular to the axis of polymerization. This plane of symmetry ensures a uniform, predictable monomer sequence that exclusively gives rise to a metallic band structure. Besides this key structural design feature, the family of oGNRs has one last trick up its sleeve.

Robust metallicity in sGNRs required the fusion of [4]helicene fragments along the sawtooth edge to induce an effective mixing of sublattice spin-polarized ZM states. The resulting broadening of the metallic ZMB (i.e., a reduced DOS at *E*_F_) proved sufficient to circumvent Mott insulator or Stoner magnetic phase transitions. In contrast, an efficient hopping between ZM states localized on the A and B sublattice sites is already built into the design of oGNRs. The −AA–BB–AA– polymerization places ZM states on alternating sublattice sites, ensuring that the hopping amplitudes *t*_1_ and *t*_2_ between adjacent states are dominated by the nearest-neighbor hopping term rather than the much smaller second nearest-neighbor hopping (Figure 1B). The sublattice mixing resulting from the fusion of [4]helicene fragments along the edges of 5-oGNRs is small and has a negligible effect on the width of the metallic ZMB (Supporting Information Figure S4A). Band structure calculations using the local spin density approximation (LSDA) show no sign of magnetic phase transitions for the disperse metallic ZM bands in 5-oGNRs (Supporting Information Figure S4B).

## Conclusions

We herein demonstrate the versatility of ZM engineering for introducing robust metallicity in 1D GNRs. A *C*_s_ symmetric molecular building block undergoes a regiocontrolled on-surface polymerization to yield homogeneous samples of 5-oGNRs featuring a symmetric superlattice of ZM states along the GNR backbone. Guided by elementary tight-binding analysis, we pioneer the design of 5-oGNRs around a strong nearest-neighbor hopping interaction between electrons in adjacent ZM states, giving rise to a large ZM bandwidth that is insensitive to Peierls and Stoner metal–insulator transitions. First-principles DFT-LDA calculations and STS corroborate the emergence of metallic ZM bands in 5-oGNRs. The design and synthesis of robust, metallic GNRs pave the way toward the realization of energy-efficient integrated circuit architectures based on low-dimensional carbon materials that are capable of high-speed electronic^37,38^ and quantum information processing.^39,40^

## Experimental Section

### Materials and Instrumentation

Unless otherwise stated, all manipulations of air- and/or moisture-sensitive compounds were carried out in an oven-dried glassware under an atmosphere of N_2_. All solvents and reagents were purchased from Alfa Aesar, Spectrum Chemicals, Acros Organics, TCI America, and Sigma-Aldrich and were used as received unless otherwise noted. Organic solvents were dried by passing through a column of alumina and were degassed by vigorous bubbling of N_2_ through the solvent for 20 min. Flash column chromatography was performed on SiliCycle silica gel (particle size 40–63 μm). Thin layer chromatography was carried out using SiliCycle silica gel 60 Å F-254 precoated plates (0.25 mm thick) and visualized by UV absorption. All ^1^H and ^13^C{^1^H} NMR spectra were recorded on a Bruker AV-600 spectrometer and are referenced to residual solvent peaks (CD_2_Cl_2_^1^H NMR = 5.32 ppm, ^13^C{^1^H} NMR = 53.84 ppm). ESI mass spectrometry was performed on a Finnigan LTQFT (Thermo) spectrometer in positive ionization mode. X-ray crystallography was performed on a Rigaku XtaLAB P200 equipped with a MicroMax 007HF dual-source rotating anode and a Pilatus 200 K hybrid pixel array detector. Data were collected using Mo-Kα (λ = 0.71073 Å) radiation. Crystals were kept at 100 K throughout the collection using an Oxford Cryostream 700 for **1b** and **8b**. Data collection was performed with CrysAlisPro.^41^ Data was processed with CrysAlisPro and includes a multi-scan absorption correction applied using the SCALE3 ABSPACK scaling algorithm within CrysAlisPro. Crystallographic data was solved with ShelXT, refined with ShelXL and finalized in Olex1.5.

#### 2-(5-Methoxy-2-(phenylethynyl)phenyl)-4,4,5,5-tetramethyl-1,3,2-dioxaborolane (**2**)

A 50 mL Schlenk flask was charged under N_2_ with 2-bromo-4-methoxy-1-(phenylethynyl)benzene (0.500 g, 1.75 mmol), bis(pinacolato)diboron (0.670 g, 2.63 mmol), and potassium acetate (0.515 g, 5.25 mmol) in dry dioxane (10 mL). The reaction mixture was degassed by sparging with N_2_ for 20 min before [1,1′-bis(diphenylphosphino)ferrocene]dichloropalladium(II) (0.07 g, 0.09 mmol) was added under N_2_. A reflux condenser was attached, and the reaction mixture was stirred under N_2_ for 18 h at 80 °C. The reaction mixture was concentrated on a rotary evaporator. Column chromatography (SiO_2_; CH_2_Cl_2_) yielded **2** (0.570 g, 1.7 mmol, 97%) as a colorless solid. ^1^H NMR (600 MHz, CD_2_Cl_2_) δ *=* 7.55 (d, *J* = 8.0 Hz, 2H), 7.49 (d, *J* = 8.0 Hz, 1H), 7.38–7.33 (m, 3H), 7.29 (d, *J* = 2.0 Hz, 1H), 6.97 (dd, *J* = 8.0 Hz, *J* = 2.0 Hz, 1H), 3.85 (s, 3H), 1.39 (s, 12H) ppm; ^13^C {^1^H} NMR (151 MHz, CD_2_Cl_2_) δ = 159.5, 134.4, 131.8, 128.9, 128.3, 124.8, 120.6, 117.1, 91.1, 90.0, 84.6, 83.8, 55.9, 25.3 ppm; HRMS (ESI-TOF) *m*/*z*: [C_21_H_24_O_3_B_1_]^+^ calcd [C_21_H_24_O_3_B_1_] 335.1813; found 335.1815.

#### 2,6-Dibromo-4-methyl-1,1′-biphenyl (**3**)

A 250 mL Schlenk flask was charged under N_2_ with *N*,*N*-diisopropylethylamine (2.0 g, 20 mmol) in dry THF (140 mL). The reaction mixture was cooled to −78 °C and stirred for 20 min. *n*-BuLi (6.2 mL, 15.5 mmol, 2.5 M in hexanes) was added dropwise and stirred for 5 min. 3,5-Dibromotoluene (3.75 g, 15 mmol) was added dropwise, and the reaction mixture was stirred for 20 min. ZnCl_2_ (2.10 g, 15.5 mmol) was added, and the reaction mixture was stirred for 2.5 h at 24 °C. Iodobenzene (1.00 g, 5 mmol) and tetrakis(triphenylphosphine)palladium(0) (0.82 g, 0.71 mmol) were added, and the reaction mixture was stirred for 18 h at 24 °C. The reaction mixture was concentrated on a rotary evaporator, diluted with H_2_O (200 mL), and extracted with CH_2_Cl_2_ (300 mL). The combined organic phases were washed with H_2_O (100 mL) and saturated aqueous NaCl (100 mL), dried over MgSO_4_, and concentrated on a rotary evaporator. Column chromatography (SiO_2_; hexane) yielded **3** (1.60 g, 4.9 mmol, 98%) as a colorless crystalline solid. ^1^H NMR (600 MHz, CD_2_Cl_2_) δ *=* 7.49 (s, 2H), 7.47–7.41 (m, 3H), 7.20 (d, *J* = 8.0 Hz, 2H), 2.36 (s, 3H) ppm; ^13^C {^1^H} NMR (151 MHz, CD_2_Cl_2_) δ = 141.7, 141.3, 140.4, 133.0, 130.0, 128.7, 128.5, 124.4, 20.8 ppm; HRMS (EI-TOF) *m*/*z*: [C_13_H_10_Br_2_]^+^ calcd [C_13_H_10_Br_2_] 325.9129; found 325.9125.

#### 5-Methoxy-3′-(5-methoxy-2-(phenylethynyl)phenyl)-5′-methyl-2-(phenylethynyl)-1,1′:2′,1″-terphenyl (**4**)

A 1000 mL Schlenk flask was charged with **2** (1.45 g, 4.3 mmol), **3** (6.48 g, 19.4 mmol), and K_2_CO_3_ (3.57 g, 25.8 mmol) in dioxane (250 mL) and H_2_O (40 mL). The reaction mixture was degassed by sparging with N_2_ for 20 min before tetrakis(triphenylphosphine)palladium(0) (0.50 g, 0.43 mmol) was added under N_2_. A reflux condenser was attached, and the reaction mixture was stirred under N_2_ for 18 h at 100 °C. The reaction mixture was concentrated on a rotary evaporator, diluted with H_2_O (200 mL), and extracted with CH_2_Cl_2_ (300 mL). The combined organic phases were washed with H_2_O (100 mL) and saturated aqueous NaCl (100 mL), dried over MgSO_4_, and concentrated on a rotary evaporator. Column chromatography (SiO_2_; 3:2 hexane/CH_2_Cl_2_) yielded **4** (1.75 g, 3.0 mmol, 70%) as a light-yellow solid. ^1^H NMR (600 MHz, CD_2_Cl_2_) δ = 7.45 (s, 2H), 7.36 (d, *J* = 8.0 Hz, 2H), 7.30–7.06 (m, 10H), 6.97–6.86 (m, 5H), 6.70 (d, *J* = 8.0 Hz, 2H), 6.58(m, 2H), 3.58 (s, 6H), 2.53 (s, 3H) ppm; ^13^C{^1^H} NMR (151 MHz, CD_2_Cl_2_) δ = 159.4, 146.7, 140.7, 140.0, 138.1, 136.2, 133.5, 131.6, 128.8, 128.2, 127.2, 126.3, 124.4, 116.6, 115.7, 113.7, 91.7, 90.1, 55.8, 21.5 ppm; HRMS (ESI-TOF) *m*/*z*: [C_43_H_33_O_2_]^+^ calcd [C_43_H_33_O_2_] 581.2475; found 581.2477.

#### 5,9-Diiodo-2,12-dimethoxy-7-methyl-6,8,14-triphenylbenzo[*m*]-tetraphene (**5**)

A 500 mL Schlenk flask was charged in the dark under N_2_ with bis(pyridine)iodonium tetrafluoroborate (2.25 g, 6.0 mmol) in dry CH_2_Cl_2_ (240 mL). Trifluoromethane sulfonic acid was added dropwise, and the reaction mixture was stirred for 15 min at 24 °C. The reaction mixture was cooled to −40 °C before **4** (1.00 g, 1.7 mmol) was added as a solution in CH_2_Cl_2_ (60 mL). The reaction mixture was stirred for 30 min at −40 °C before being warmed to 24 °C over 1.5 h. The reaction mixture was diluted with saturated aqueous Na_2_S_2_O_3_ (200 mL) and extracted with CH_2_Cl_2_ (300 mL). The combined organic phases were washed with H_2_O (100 mL) and saturated aqueous NaCl (100 mL), dried over MgSO_4_, and concentrated on a rotary evaporator. The crude solid was dissolved in a minimum amount of CH_2_Cl_2_, filtered over a short pad of SiO_2_, and precipitated by trituration with MeOH, yielding **5** (0.971 g, 1.17 mmol, 68%) as a yellow solid. ^1^H NMR (600 MHz, CD_2_Cl_2_) δ *=* 8.19 (d, *J* = 8.0 Hz, 2H), 7.55 (d, *J* = 8.0 Hz, 2H), 7.51–7.47 (m, 3H), 7.30–7.29 (m, 6H), 7.14–7.13 (m, 4H), 6.96 (dd, *J* = 8.0 Hz, *J* = 2.0 Hz, 2H), 6.76 (d, *J* = 2.0 Hz, 2H), 3.23 (s, 6H), 1.37 (s, 3H) ppm; ^13^C{^1^H} NMR (151 MHz, CD_2_Cl_2_) δ = 157.1, 148.8, 144.9, 141.7, 135.2, 134.1, 133.5, 133.4, 132.4, 131.8, 131.4, 130.7, 130.0, 129.1, 128.4, 128.3, 127.9, 117.7, 111.7, 108.5, 55.5, 23.2 ppm; HRMS (ESI-TOF) *m*/*z*: [C_43_H_30_O_2_I_2_]^+^ calcd [C_43_H_30_O_2_I_2_] 832.0330; found 832.0331.

#### 2,12-Dimethoxy-7-methyl-6,8,14-triphenylbenzo[*m*]tetraphene (**6**)

A 500 mL Schlenk flask was charged under N_2_ with **5** (0.95 g, 1.14 mmol) in dry THF (120 mL). The reaction mixture was cooled to −78 °C and stirred for 20 min. *s*-BuLi (16.3 mL, 22.8 mmol, 1.4 M in cyclohexane) was added dropwise, and the reaction mixture was stirred for 5 min at −78 °C. The reaction mixture was quenched by rapid addition of MeOH (10 mL). The reaction mixture was concentrated on a rotary evaporator, diluted with H_2_O (200 mL), and extracted with CH_2_Cl_2_ (300 mL). The combined organic phases were washed with H_2_O (100 mL) and saturated aqueous NaCl (100 mL), dried over MgSO_4_, and concentrated on a rotary evaporator. The crude solid was dissolved in a minimum amount of CH_2_Cl_2_, filtered over a short pad of SiO_2_, and concentrated on a rotary evaporator. The crude solid was sonicated in a minimum amount of pentane, filtered, and washed with a minimum amount of pentane, yielding **6** (0.450 g, 0.77 mmol, 68%) as a yellow solid. ^1^H NMR (600 MHz, CD_2_Cl_2_) δ *=* 7.66 (d, *J* = 8.0 Hz, 2H), 7.60 (m, 2H), 7.55 (m, 2H), 7.50 (m, 3H), 7.31 (m, 8H), 7.26 (m, 2H), 6.96 (dd, *J* = 8.0 Hz, *J* = 2.0 Hz, 2H), 6.81 (d, *J* = 2.0 Hz, 2H), 3.24 (s, 6H), 1.78 (s, 3H) ppm; ^13^C{^1^H} NMR (151 MHz, CD_2_Cl_2_) δ = 156.5, 146.0, 145.8, 136.3, 134.7, 134.0, 132.7, 131.8, 131.4, 130.8, 130.5, 129.5, 129.1, 129.1, 128.8, 128.4, 127.7, 127.0, 117.1, 112.2, 55.3, 25.1 ppm; HRMS (ESI-TOF) *m*/*z*: [C_43_H_32_O_2_]^+^ calcd [C_43_H_32_O_2_] 580.2397; found 580.2389.

#### 7-Methylene-6,8,14-triphenyl-7,14-dihydrobenzo[*m*]tetraphene-2,12-diol (**7b**)

A 100 mL Schlenk flask was charged under N_2_ with **6** (0.375 g, 0.65 mmol) in dry DMF (16 mL). NaSEt (0.540 g, 6.5 mmol) was added under N_2_ as a solid in one portion. The reaction mixture was stirred under N_2_ for 3 h at 153 °C. The reaction mixture was quenched with 1 M HCl, causing the crude product to precipitate. The crude solid was isolated by filtration and washed with 1 M HCl (50 mL) and H_2_O (100 mL). The crude solid was dissolved in a minimum amount of CH_2_Cl_2_ and precipitated by trituration with hexanes, yielding **7b** (0.200 g, 0.36 mmol, 56%) as a colorless solid. ^1^H NMR (600 MHz, CD_2_Cl_2_) δ *=* 7.95 (d, *J* = 2.0 Hz, 2H), 7.81 (d, *J* = 8.0 Hz, 2H), 7.67 (s, 2H), 7.46 (m, 4H), 7.40–7.35 (m, 6H), 7.33–7.30 (m, 2H), 7.16 (dd, *J* = 8.0 Hz, *J* = 2.0 Hz, 2H), 7.07–7.02 (m, 3H), 6.74 (s, 1H), 5.55 (s, 2H), 4.89 (s, 2H) ppm; ^13^C{^1^H} NMR (151 MHz, CD_2_Cl_2_) δ = 155.0, 143.6, 143.1, 139.8, 136.4, 135.6, 134.7, 132.1, 131.4, 130.6 (2C), 128.8, 128.6, 128.5, 128.5, 127.0, 127.0, 125.8, 118.4, 106.9, 42.9 ppm; (ESI-TOF) *m*/*z*: [C_41_H_27_O_2_]^+^ calcd [C_41_H_27_O_2_] 551.2017; found 551.2009.

#### 7-Methylene-6,8,14-triphenyl-7,14-dihydrobenzo[*m*]tetraphene-2,12-diyl bis(trifluoromethanesulfonate) (**8b**)

A 100 mL Schlenk flask was charged under N_2_ with **7b** (0.190 g, 0.34 mmol) in dry CH_2_Cl_2_ (34 mL). The reaction mixture was cooled to 0 °C. Et_3_N (0.425 g, 4.2 mmol) was added dropwise under N_2_, and the reaction mixture was stirred at 0 °C for 15 min. Trifluoromethanesulfonic anhydride (0.593 g, 2.1 mmol) was added dropwise under N_2_. The reaction mixture was warmed to 24 °C and stirred for 1.5 h at 24 °C. The reaction mixture was concentrated on a rotary evaporator. The crude solid was dissolved in a minimum amount of 1:1 hexanes/CH_2_Cl_2_, filtered over a short pad of SiO_2_, and concentrated on a rotary evaporator, yielding **8b** (0.277 g, 0.34 mmol, 99%) as a colorless solid. ^1^H NMR (600 MHz, CD_2_Cl_2_) δ *=* 8.50 (d, *J* = 2.0 Hz, 2H), 8.01 (d, *J* = 8.0 Hz, 2H), 7.84 (s, 2H), 7.49–7.36 (m, 14H), 7.13–7.06 (m, 3H), 6.70 (s, 1H), 5.04 (s, 2H) ppm; ^13^C{^1^H} NMR (151 MHz, CD_2_Cl_2_) δ = 148.8, 142.8, 142.2, 140.2, 138.9, 136.1, 132.3, 132.2, 130.9 (2C), 130.4, 129.3, 128.7, 128.6, 127.8, 127.6, 127.6, 120.6, 120.5, 118.5, 116.2, 43.9 ppm; (ESI-TOF) *m*/*z*: [C_43_H_27_O_6_F_6_S_2_] + calcd [C_43_H_27_O_6_F_6_S_2_] 817.1148; found 817.1152.

#### 2,2′-(7-Methylene-6,8,14-triphenyl-7,14-dihydrobenzo[*m*]-tetraphene-2,12-diyl)bis(4,4,5,5-tetramethyl-1,3,2-dioxaborolane) (**9b**)

A 50 mL Schlenk flask was charged under N_2_ with **8b** (0.130 g, 0.16 mmol), bis(pinacolato)diboron (0.254 g, 0.96 mmol), and KOAc (0.300 g, 2.88 mmol) in dry dioxane (15 mL). The reaction mixture was degassed by sparging with N_2_ for 20 min before [1,1′-bis(diphenylphosphino)-ferrocene]dichloropalladium(II) (0.013 g, 0.02 mmol) was added under N_2_. A reflux condenser was attached, and the reaction mixture was stirred under N_2_ for 18 h at 80 °C. The reaction mixture was concentrated on a rotary evaporator. Column chromatography (SiO_2_; CH_2_Cl_2_) yielded **9b** (0.096 g, 0.12 mmol, 78%) as a colorless solid. ^1^H NMR (600 MHz, CD_2_Cl_2_) δ *=* 9.24 (s, 2H), 7.89–7.85 (m, 4H), 7.77 (s, 2H), 7.53 (d, *J* = 8.0 Hz, 4H), 7.43 (t, *J* = 8.0 Hz, 4H), 7.38–7.34 (m, 4H), 7.28 (s, 1H), 7.08–7.01 (m, 3H), 4.90 (s, 2H), 1.49 (s, 24H) ppm; ^13^C{^1^H} NMR (151 MHz, CD_2_Cl_2_) δ = 144.3, 143.1, 139.8, 139.6, 137.0, 135.0, 134.7, 132.7, 131.1, 130.7, 130.5, 130.2, 129.1, 128.6, 128.5, 128.4, 127.2, 126.8, 125.7, 84.6, 42.7, 25.5, 25.4 ppm; (ESI-TOF) *m*/*z*: [C_53_H_51_O_4_B_2_]^+^ calcd [C_53_H_51_O_4_B_2_] 773.3968; found 773.3961.

#### 2,12-Dibromo-7-methylene-6,8,14-triphenyl-7,14-dihydrobenzo-[*m*]tetraphene (**1b**)

A 25 mL sealable Schlenk tube was charged under N_2_ with **9b** (0.040 g, 0.05 mmol) and CuBr_2_ (0.070 g, 0.31 mmol) in THF (1 mL), MeOH (2 mL), and H_2_O (2 mL). The reaction mixture was degassed by sparging with N_2_ for 20 min. The reaction mixture was sealed under N_2_ and stirred for 18 h at 120 °C. The reaction mixture was concentrated on a rotary evaporator, diluted with H_2_O (10 mL), and extracted with CH_2_Cl_2_ (30 mL). The combined organic phases were washed with H_2_O (10 mL) and saturated aqueous NaCl (10 mL), dried over MgSO_4_, and concentrated on a rotary evaporator. Column chromatography (SiO_2_; 4:1 hexane/CH_2_Cl_2_) yielded **1b** (0.034 g, 0.05 mmol, 96%) as a colorless solid. ^1^H NMR (600 MHz, CD_2_Cl_2_) δ *=* 8.78 (s, 2H), 7.79–7.73 (m, 4H), 7.63 (d, *J* = 8.0 Hz, 2H), 7.47–7.26 (m, 12H), 7.11–7.05 (m, 3H), 6.82 (s, 1H), 4.93 (s, 2H) ppm; ^13^C{^1^H} NMR (151 MHz, CD_2_Cl_2_) δ = 142.9, 142.6, 139.2, 139.1, 135.9, 135.1, 132.0, 131.7, 131.1, 130.8, 130.4, 130.1, 129.0, 128.6, 128.5, 127.4, 127.2, 126.8, 126.7, 121.7, 42.8 ppm; HRMS (EI-TOF) *m*/*z*: [C_41_H_26_Br_2_]^+^ calcd [C_41_H_26_Br_2_] 678.0381; found 678.0381.

### 5-oGNR Growth on Au(111) Surfaces

5-oGNRs were grown on Au(111)/mica films under UHV conditions. Atomically clean Au(111) surfaces were prepared through iterative Ar^+^ sputter/anneal cycles. A sub-monolayer coverage of **1b** on atomically clean Au(111) was obtained by sublimation at crucible temperatures of 453–473 K using a Knudsen cell evaporator. After deposition, the surface temperature was slowly ramped (≤2 K min^–1^) to 453 K and held at this temperature for 15 min to induce the radical step-growth polymerization and then ramped slowly (≤2 K min^–1^) to 623 K and held there for 15 min to induce cyclodehydrogenation.

### Scanning Tunneling Microscopy and Spectroscopy

All STM experiments were performed using a commercial OMICRON LT-STM operating at *T* = 4 K using PtIr STM tips. STM tips were optimized for STM using an automated tip conditioning program.^42^ d*I*/d*V* measurements were recorded with CO-functionalized STM tips using a lock-in amplifier with a modulation frequency of *f* = 455 Hz and a modulation amplitude of *V*_ac_ = 11 mV. d*I*/d*V* point spectra were recorded under open feedback loop conditions. d*I*/d*V* maps were collected under constant height conditions. Peak positions in d*I*/d*V* point spectroscopy were determined by fitting the spectra with Lorentzian peaks. Each peak position is based on an average of ∼10 spectra collected on various GNRs with different tips, all of which were first calibrated to the Au(111) Shockley surface state.

### Calculations

First-principles DFT calculations in the LDA and LSDA approximations were implemented using the Quantum Espresso package.^43^ We used Norm-conserving pseudopotentials with a 60 Ry energy cut-off and 0.005 Ry Gaussian broadening. To ensure the accuracy of our results, a sufficiently large vacuum region was included in the supercell calculation. All of the dangling bonds at the edge of the carbon skeleton were hydrogenated. The structures were first fully relaxed until all components of the force were smaller than 0.01 eV/Å.

## Supporting Information Available

Experimental details, STM/BRSTM images, additional spectroscopic data on 5-oGNRs, calculations, and ^1^H and ^13^C NMR spectra for all compounds (PDF)
