# Supplementary material for: Engineering Robust Metallic Zero-Mode States in Olympicene Graphene Nanoribbons
Source: J Am Chem Soc. 2023 Jul 10;145(28):15162–70. doi: 10.1021/jacs.3c01576 (PMC10360063; doi:10.1021/jacs.3c01576)
Supplement: Supplementary file 1 — ja3c01576_si_001.pdf [file ja3c01576_si_001.pdf]

Engineering Robust Metallic Zero-Mode States in Olympicene Graphene Nanoribbons

Ryan D. McCurdy,[†,‡] Aidan Delgado,[†,‡] Jingwei Jiang,[‡,§,‡] Junmian Zhu,[†] Ethan Chi Ho Wen,[†] Raymond E. Blackwell,[†] Gregory C. Veber,[†] Shenkai Wang,[†] Steven G. Louie,[‡,§,*] Felix R. Fischer[†,§,¶,◊,*]

[†]Department of Chemistry, University of California, Berkeley, CA 94720, U.S.A.

[‡]Department of Physics, University of California, Berkeley, CA 94720, U.S.A.

[§]Materials Sciences Division, Lawrence Berkeley National Laboratory, Berkeley, CA 94720, U.S.A.

[¶]Kavli Energy NanoSciences Institute at the University of California Berkeley and the Lawrence Berkeley National Laboratory, Berkeley, California 94720, U.S.A.

[◊]Bakar Institute of Digital Materials for the Planet, Division of Computing, Data Science, and Society, University of California, Berkeley, CA 94720, USA.

**1.**	**Figure S1.** Bottom-up synthesis of 5-oGNRs.	**S3**
**2.**	**Figure S2.** Bond resolved scanning tunneling microscopy images of 5-oGNRs.	**S4**
**3.**	**Figure S3.** Electronic structure of 5-oGNRs.	**S5**
**4.**	**Figure S4.** DFT-LSDA calculated band structure for 5-oGNRs.	**S6**
**5.**	**Figure S5.** ^{1}H NMR (600 MHz, CD_2Cl_2) of 2-(5-methoxy-2-(phenylethynyl)phenyl)-4,4,5,5-tetramethyl-1,3,2-dioxaborolane (**2**) at 24 °C.	**S7**
**6.**	**Figure S6.** ^{13}C {^{1}H} NMR (151 MHz, CD_2Cl_2) spectrum of 2-(5-methoxy-2-(phenylethynyl)phenyl)-4,4,5,5-tetramethyl-1,3,2-dioxaborolane (**2**) at 24 °C.	**S8**
**7.**	**Figure S7.** ^{1}H NMR (600 MHz, CD_2Cl_2) of 2,6-dibromo-4-methyl-1,1'-biphenyl (**3**) at 24 °C.	**S9**
**8.**	**Figure S8.** ^{13}C {^{1}H} NMR (151 MHz, CD_2Cl_2) spectrum of 2,6-dibromo-4-methyl-1,1'-biphenyl (**3**) at 24 °C.	**S10**
**9.**	**Figure S9.** ^{1}H NMR (600 MHz, CD_2Cl_2) of 5-methoxy-3'-(5-methoxy-2-(phenylethynyl)phenyl)-5'-methyl-2-(phenylethynyl)-1,1':2',1''-terphenyl (**4**) at 24 °C.	**S11**
**10.**	**Figure S10.** ^{13}C {^{1}H} NMR (151 MHz, CD_2Cl_2) spectrum of 5-methoxy-3'-(5-methoxy-2-(phenylethynyl)phenyl)-5'-methyl-2-(phenylethynyl)-1,1':2',1''-terphenyl (**4**) at 24 °C.	**S12**
**11.**	**Figure S11.** ^{1}H NMR (600 MHz, CD_2Cl_2) of 5,9-diiodo-2,12-dimethoxy-7-methyl-6,8,14-triphenylbenzo[m]tetraphene (**5**) at 24 °C.	**S13**
**12.**	**Figure S12.** ^{13}C {^{1}H} NMR (151 MHz, CD_2Cl_2) spectrum of 5,9-diiodo-2,12-dimethoxy-7-methyl-6,8,14-triphenylbenzo[m]tetraphene (**5**) at 24 °C.	**S14**
**13.**	**Figure S13.** ^{1}H NMR (600 MHz, CD_2Cl_2) of 2,12-dimethoxy-7-methyl-6,8,14-triphenylbenzo[m]tetraphene (**6**) at 24 °C.	**S15**
**14.**	**Figure S14.** ^{13}C {^{1}H} NMR (151 MHz, CD_2Cl_2) spectrum of 2,12-dimethoxy-7-methyl-6,8,14-triphenylbenzo[m]tetraphene (**6**) at 24 °C.	**S16**
**15.**	**Figure S15.** ^{1}H NMR (600 MHz, CD_2Cl_2) of 7-methylene-6,8,14-triphenyl-7,14-dihydrobenzo[m]tetraphene-2,12-diol (**7b**) at 24 °C.	**S17**
**16.**	**Figure S16.** ^{13}C {^{1}H} NMR (151 MHz, CD_2Cl_2) spectrum of 7-methylene-6,8,14-triphenyl-7,14-dihydrobenzo[m]tetraphene-2,12-diol (**7b**) at 24 °C	**S18**
**17.**	**Figure S17.** ^{1}H NMR (600 MHz, CD_2Cl_2) of 7-methylene-6,8,14-triphenyl-7,14-dihydrobenzo[m]tetraphene-2,12-diyl bis(trifluoromethanesulfonate) (**8b**) at 24 °C.	**S19**

**18.**	**Figure S18.** ^{13}C {^{1}H} NMR (151 MHz, CD_2Cl_2) spectrum of 7-methylene-6,8,14-triphenyl-7,14-dihydrobenzo[m]tetraphene-2,12-diyl bis(trifluoromethanesulfonate) (**8b**) at 24 °C.	**S20**
**19.**	**Figure S19.** ^{1}H NMR (600 MHz, CD_2Cl_2) of 2,2'-(7-methylene-6,8,14-triphenyl-7,14-dihydrobenzo[m]tetraphene-2,12-diyl)bis(4,4,5,5-tetramethyl-1,3,2-dioxaborolane) (**9b**) at 24 °C.	**S21**
**20.**	**Figure S20.** ^{13}C {^{1}H} NMR (151 MHz, CD_2Cl_2) spectrum of 2,2'-(7-methylene-6,8,14-triphenyl-7,14-dihydrobenzo[m]tetraphene-2,12-diyl)bis(4,4,5,5-tetramethyl-1,3,2-dioxaborolane) (**9b**) at 24 °C.	**S22**
**21.**	**Figure S21.** ^{1}H NMR (600 MHz, CD_2Cl_2) of 2,12-dibromo-7-methylene-6,8,14-triphenyl-7,14-dihydrobenzo[m]tetraphene (**1b**) at 24 °C.	**S23**
**22.**	**Figure S22.** ^{13}C {^{1}H} NMR (151 MHz, CD_2Cl_2) spectrum of 2,12-dibromo-7-methylene-6,8,14-triphenyl-7,14-dihydrobenzo[m]tetraphene (**1b**) at 24 °C.	**S24**
**23.**	**Figure S23.** Single crystal X-ray structure diagram of 7-methylene-6,8,14-triphenyl-7,14-dihydrobenzo[m]tetraphene-2,12-diyl bis(trifluoromethanesulfonate) (**8b**).	**S25**
**24.**	**Table S1.** Crystal data and structure refinement for 7-methylene-6,8,14-triphenyl-7,14-dihydrobenzo[m]tetraphene-2,12-diyl bis(trifluoromethanesulfonate) (**8b**).	**S25**
**25.**	**Table S2.** Atomic coordinates ($\times 10^4$) and equivalent isotropic displacement parameters ($Å^2 \times 10^3$) for 7-methylene-6,8,14-triphenyl-7,14-dihydrobenzo[m]tetraphene-2,12-diyl bis(trifluoromethanesulfonate) (**8b**).	**S26**
**26.**	**Table S3.** Bond lengths [Å] and angles [°] for 7-methylene-6,8,14-triphenyl-7,14-dihydrobenzo[m]tetraphene-2,12-diyl bis(trifluoromethanesulfonate) (**8b**).	**S27**
**27.**	**Table S4.** Anisotropic displacement parameters ($Å^2 \times 10^3$) for 7-methylene-6,8,14-triphenyl-7,14-dihydrobenzo[m]tetraphene-2,12-diyl bis(trifluoromethanesulfonate) (**8b**).	**S31**
**28.**	**Table S5.** Hydrogen coordinates ($\times 10^4$) and isotropic displacement parameters ($Å^2 \times 10^3$) for 7-methylene-6,8,14-triphenyl-7,14-dihydrobenzo[m]tetraphene-2,12-diyl bis(trifluoromethanesulfonate) (**8b**).	**S32**
**29.**	**Table S6.** Torsion angles [°] for 7-methylene-6,8,14-triphenyl-7,14-dihydrobenzo[m]tetraphene-2,12-diyl bis(trifluoromethanesulfonate) (**8b**).	**S33**
**30.**	**Figure S24.** Single crystal X-ray structure diagram of 2,12-dibromo-7-methylene-6,8,14-triphenyl-7,14-dihydrobenzo[m]tetraphene (**1b**). ORTEP thermal ellipsoids are at the 50% probability level.	**S36**
**31.**	**Table S7.** Crystal data and structure refinement for 2,12-dibromo-7-methylene-6,8,14-triphenyl-7,14-dihydrobenzo[m]tetraphene (**1b**).	**S36**
**32.**	**Table S8.** Atomic coordinates ($\times 10^4$) and equivalent isotropic displacement parameters ($Å^2 \times 10^3$) for 2,12-dibromo-7-methylene-6,8,14-triphenyl-7,14-dihydrobenzo[m]tetraphene (**1b**).	**S37**
**33.**	**Table S9.** Bond lengths [Å] and angles [°] for 2,12-dibromo-7-methylene-6,8,14-triphenyl-7,14-dihydrobenzo[m]tetraphene (**1b**).	**S39**
**34.**	**Table S10.** Anisotropic displacement parameters ($Å^2 \times 10^3$) for 2,12-dibromo-7-methylene-6,8,14-triphenyl-7,14-dihydrobenzo[m]tetraphene (**1b**).	**S46**
**35.**	**Table S11.** Hydrogen coordinates ($\times 10^4$) and isotropic displacement parameters ($Å^2 \times 10^3$) for 2,12-dibromo-7-methylene-6,8,14-triphenyl-7,14-dihydrobenzo[m]tetraphene (**1b**).	**S48**
**36.**	**Table S12.** Torsion angles [°] for 2,12-dibromo-7-methylene-6,8,14-triphenyl-7,14-dihydrobenzo[m]tetraphene (**1b**).	**S49**

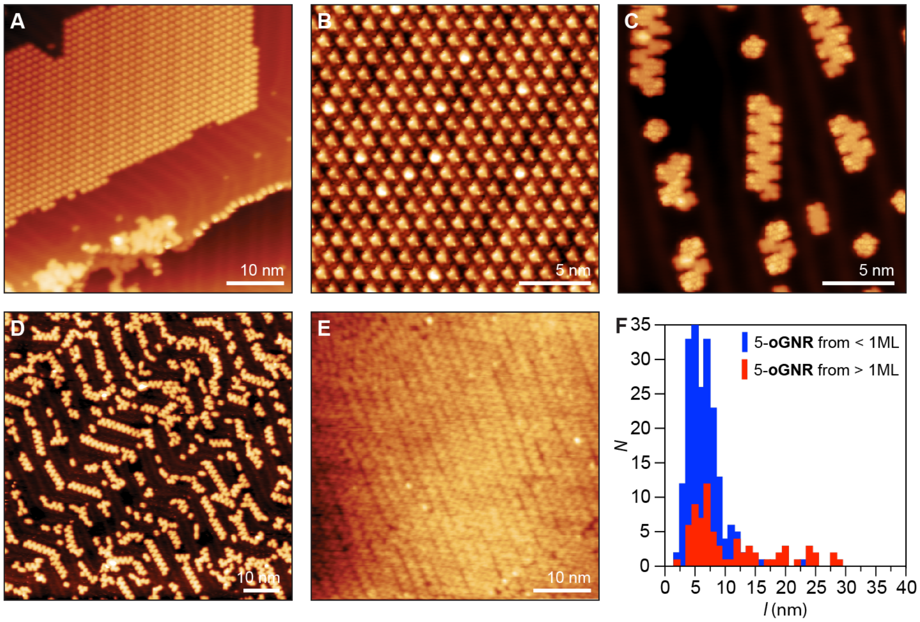

Figure S1. Bottom-up synthesis of 5-oGNRs. (A) STM topographic image of a self-assembled island of molecular precursor **1b** on Au(111) (V_s = 0.05 V, I_t = 20 pA). (B) Magnification of a self-assembled island of molecular precursor **1b** on Au(111) (V_s = 0.05 V, I_t = 20 pA). (B) STM topographic image of partially cyclodehydrogenated sample of oGNRs following annealing to 180 °C (V_s = 0.05 V, I_t = 20 pA). Bright spots lining the edges of oGNRs correspond to the protruding phenyl rings of uncyclized [4]-helicene fragments. (D) STM topographic image of a low coverage sample of 5-oGNRS following annealing to 350 °C (V_s = 0.05 V, I_t = 20 pA). (E) STM topographic image of a high coverage (>1 ML) sample of 5-oGNRs following annealing to 350 °C (V_s = 0.05 V, I_t = 20 pA). (F) Statistical distribution of 5-oGNR lengths grown from a sub-monolayer (ML) sample of precursor **1b** (blue) and a >1 ML deposition of **1b** (red).

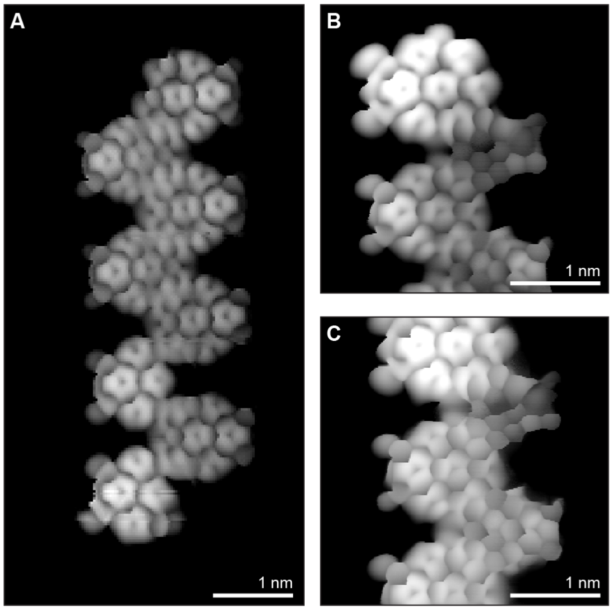

Figure S2. Bond resolved scanning tunneling microscopy images of 5-oGNRs. (A) BRSTM image of 5-oGNRs showing the 5-membered rings resulting from the fusion of [4]-helicene groups along the oGNRs edges (V_s = 0.01 V, I_t = 300 pA). (B) BRSTM image of a 5-oGNR segment showing the 5-membered rings resulting from the fusion of [4]-helicene groups along the oGNRs edges (V_s = 0.01 V, I_t = 400 pA). (C) BRSTM image of a 5-oGNR segment showing the 5-membered rings resulting from the fusion of [4]-helicene groups along the oGNRs edges (V_s = 0.01 V, I_t = 670 pA).

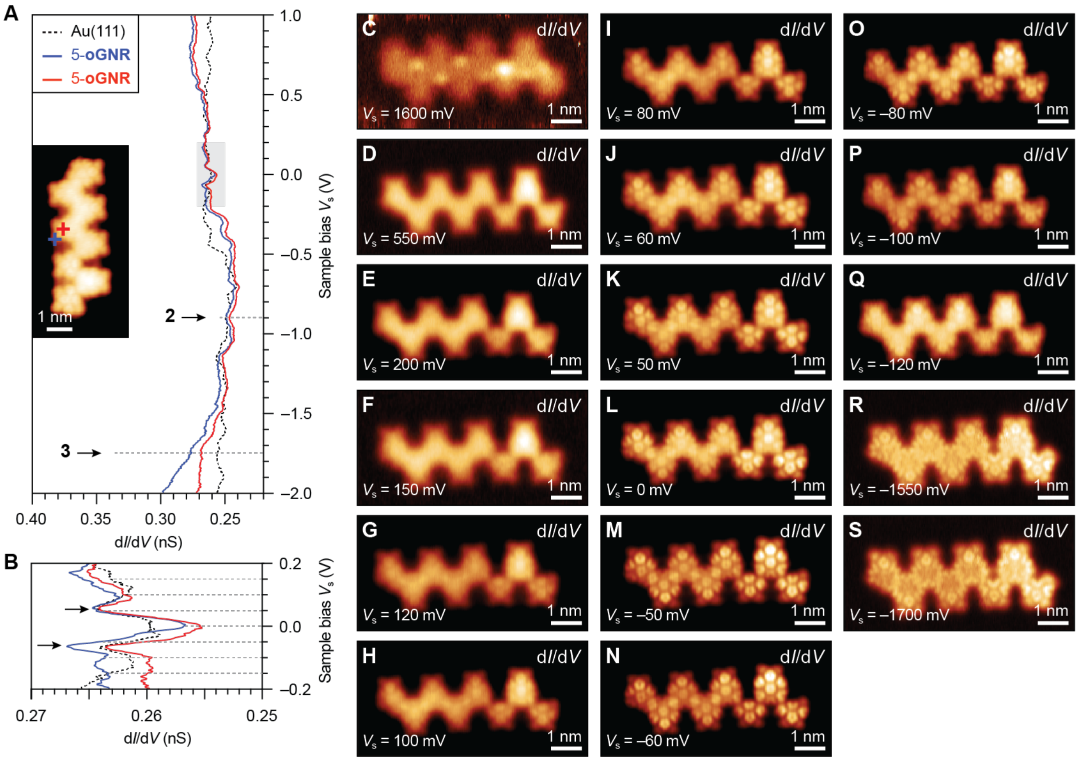

Figure S3. Electronic structure of 5-oGNRs. (A–B) STS dI/dV spectra recorded on a 5-oGNR at the positions marked in the inset STM topographic image with a red and blue cross (spectroscopy: V_{ac} = 11 mV, f = 455 Hz; imaging: V_s = 50 mV, I_t = 20 pA, CO-functionalized tip). (C–S) Constant height dI/dV maps recorded at the indicated biases (spectroscopy: V_{ac} = 11 mV, f = 455 Hz).

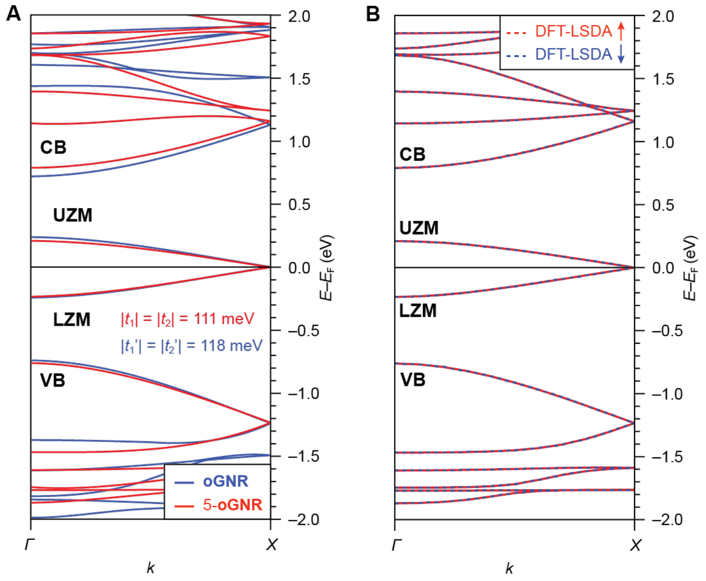

Figure S4. (A) DFT-LSDA calculated band structure for oGNRs and 5-oGNRs. The sublattice mixing of A and B sites is built into the design of oGNRs. The closure of the 5-membered ring along the edges in 5-oGNRs has a negligible effect on the width of the metallic band. (B) DFT-LSDA calculated band structure for 5-oGNRs. Lieb's theorem raises the possibility of antiferromagnetic ordering, but the large overlap between adjacent zero-mode states favors a non-magnetic ground state in oGNRs.

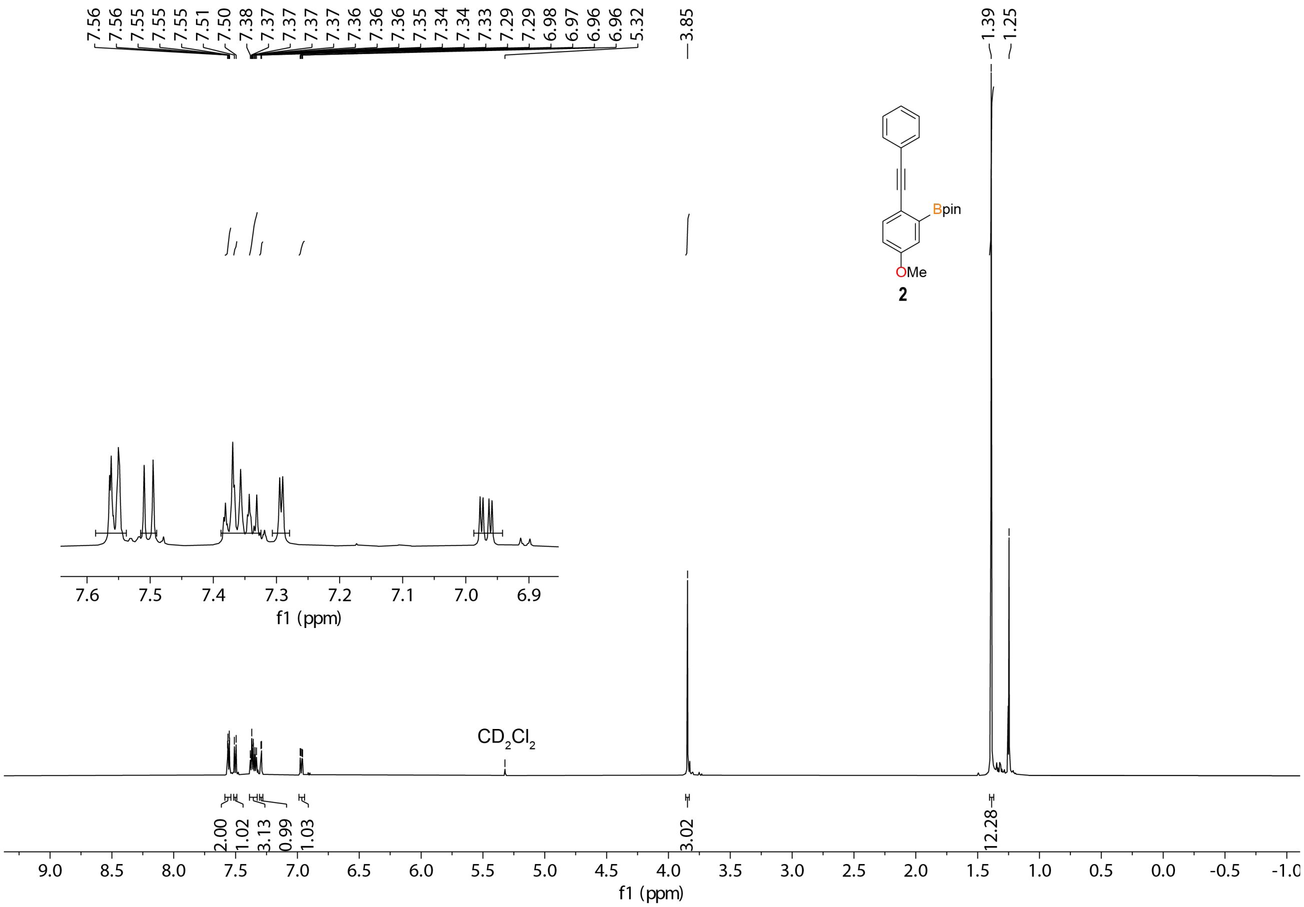

Figure S5. ^{1}H NMR (600 MHz, CD_2Cl_2) of 2-(5-methoxy-2-(phenylethynyl)phenyl)-4,4,5,5-tetramethyl-1,3,2-dioxaborolane (**2**) at 24 °C.

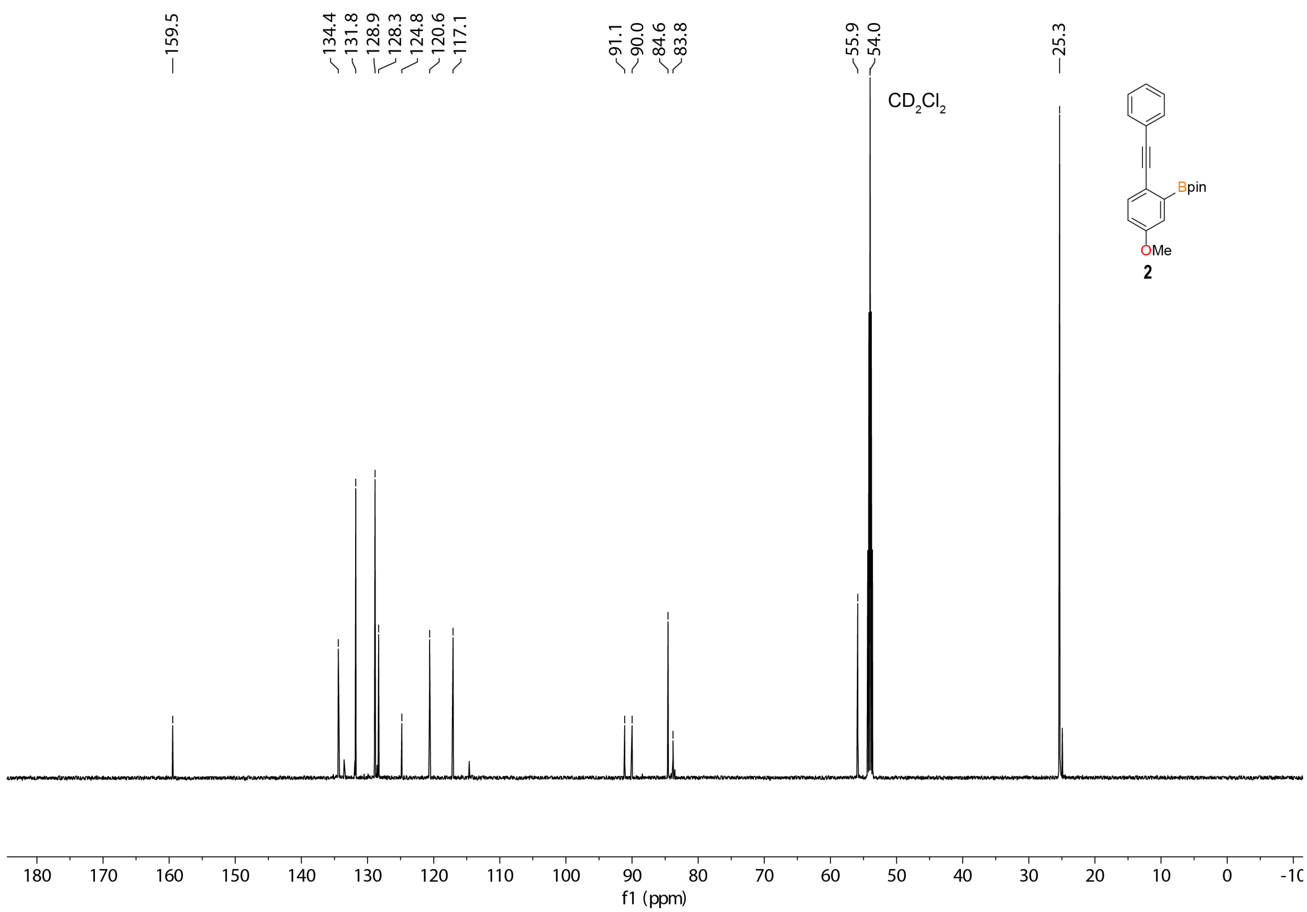

Figure S6. $^{13}C\ \{^{1}H\}$ NMR (151 MHz, CD_2Cl_2) spectrum of 2-(5-methoxy-2-(phenylethynyl)phenyl)-4,4,5,5-tetramethyl-1,3,2-dioxaborolane (**2**) at 24 °C.

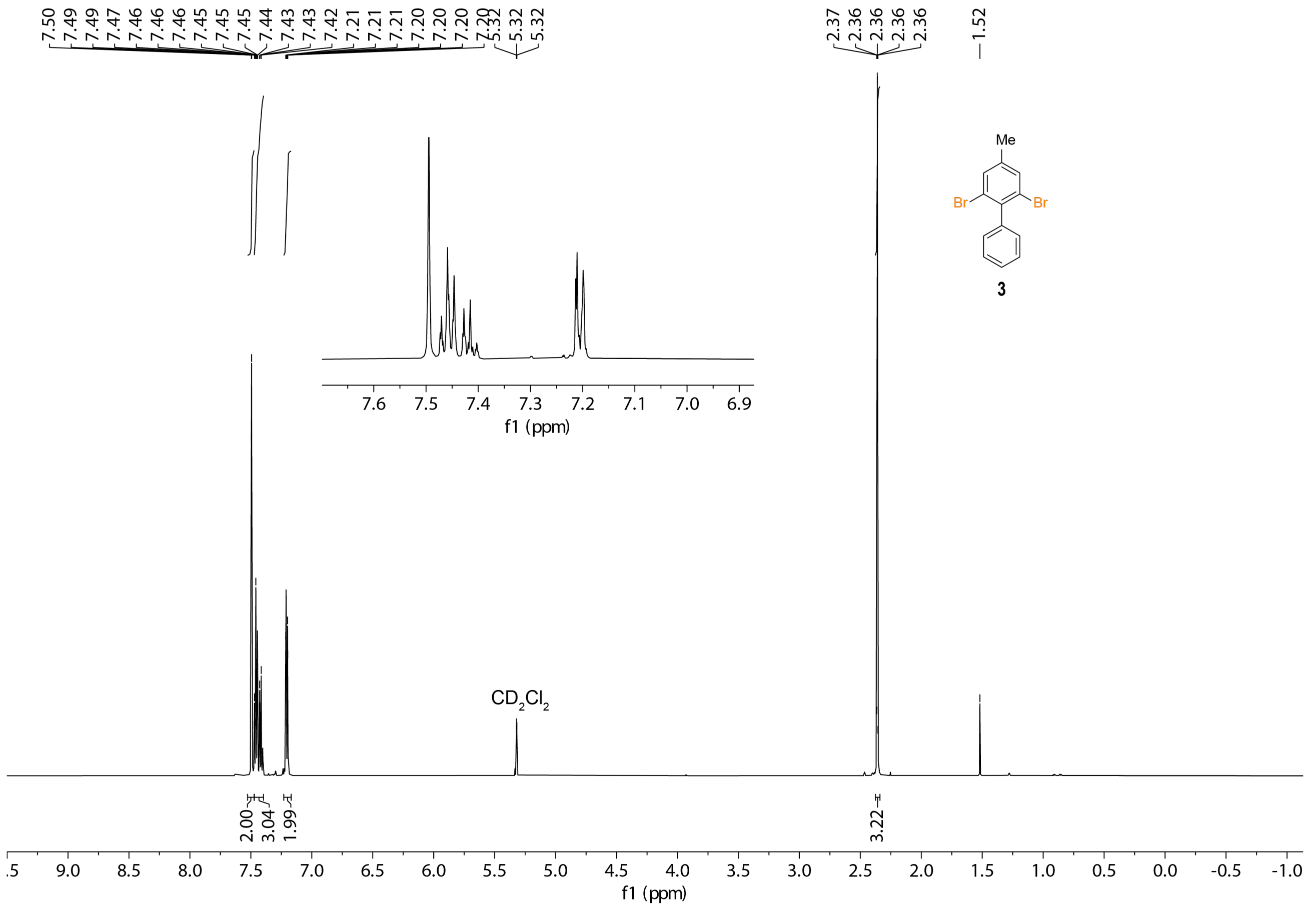

Figure S7. ^{1}H NMR (600 MHz, CD_2Cl_2) of 2,6-dibromo-4-methyl-1,1'-biphenyl (**3**) at 24 °C.

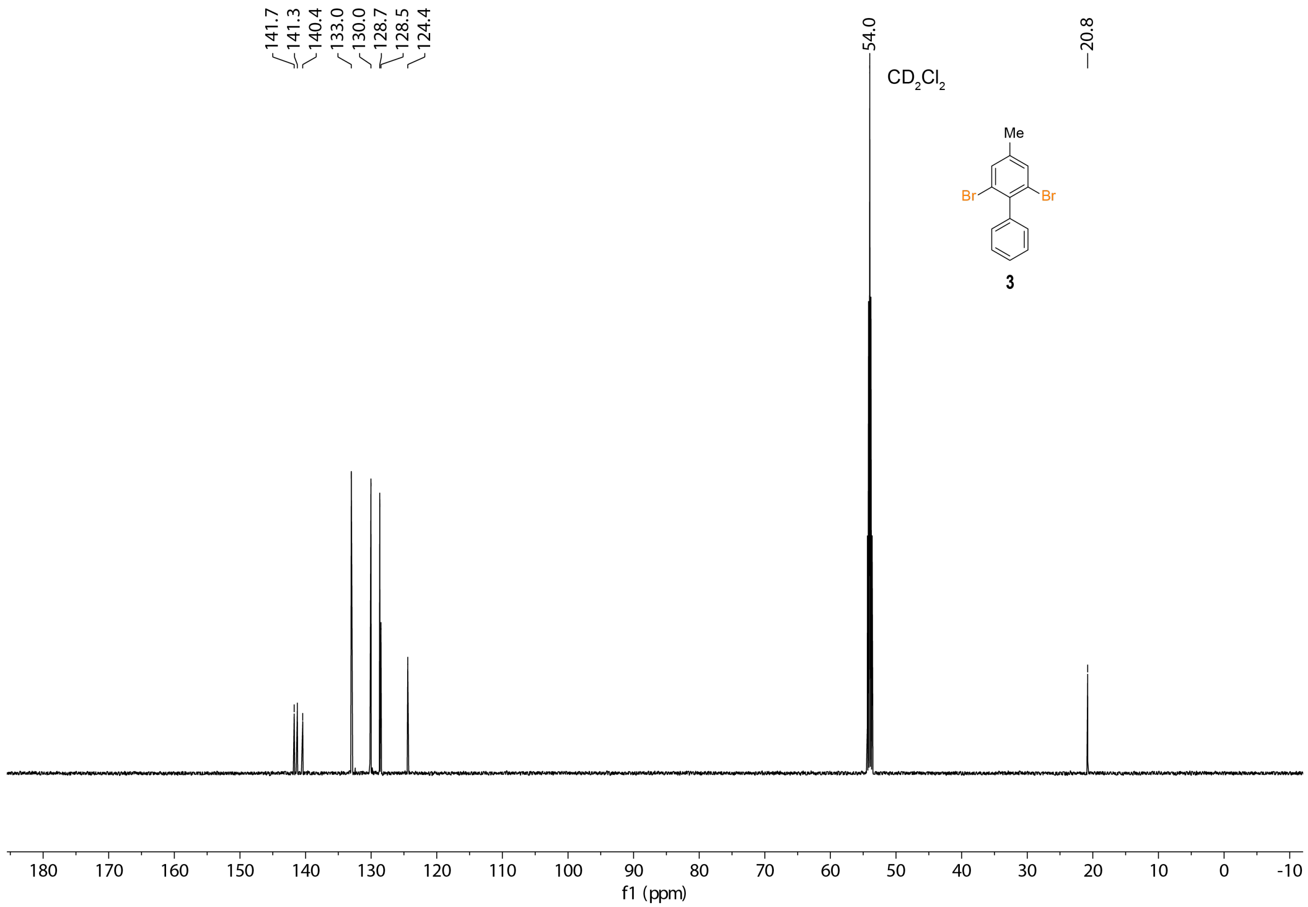

Figure S8. $^{13}C\{^1H\}$ NMR (151 MHz, CD_2Cl_2) spectrum of 2,6-dibromo-4-methyl-1,1'-biphenyl (**3**) at 24 °C.

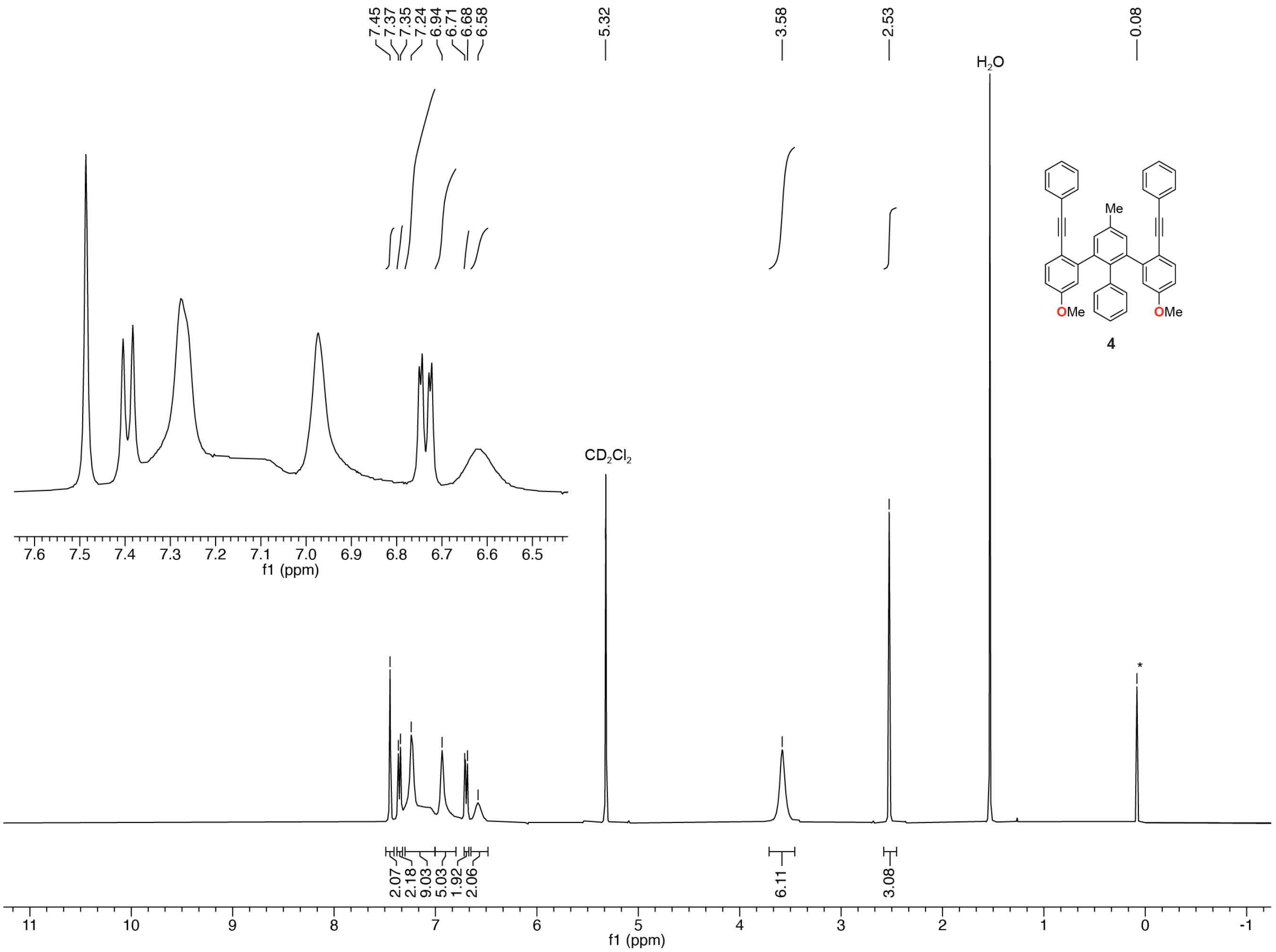

Figure S9. ^{1}H NMR (600 MHz, CD_2Cl_2) of 5-methoxy-3'-(5-methoxy-2-(phenylethynyl)phenyl)-5'-methyl-2-(phenylethynyl)-1,1':2',1''-terphenyl (**4**) at 24 °C (* silicon grease).

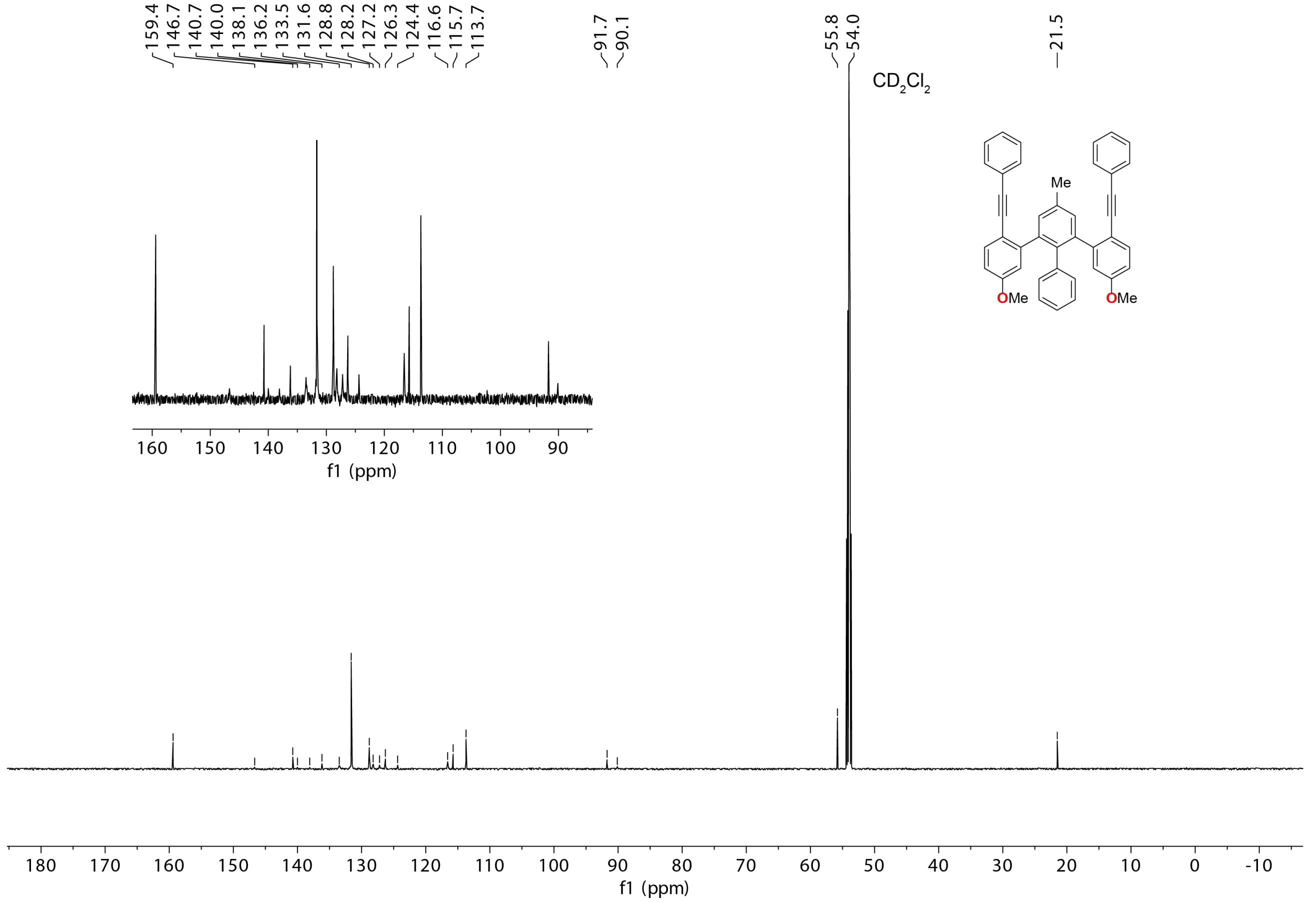

Figure S10. ^{13}C {^{1}H} NMR (151 MHz, CD_2Cl_2) spectrum of 5-methoxy-3'-(5-methoxy-2-(phenylethynyl)phenyl)-5'-methyl-2-(phenylethynyl)-1,1':2',1''-terphenyl (**4**) at 24 °C.

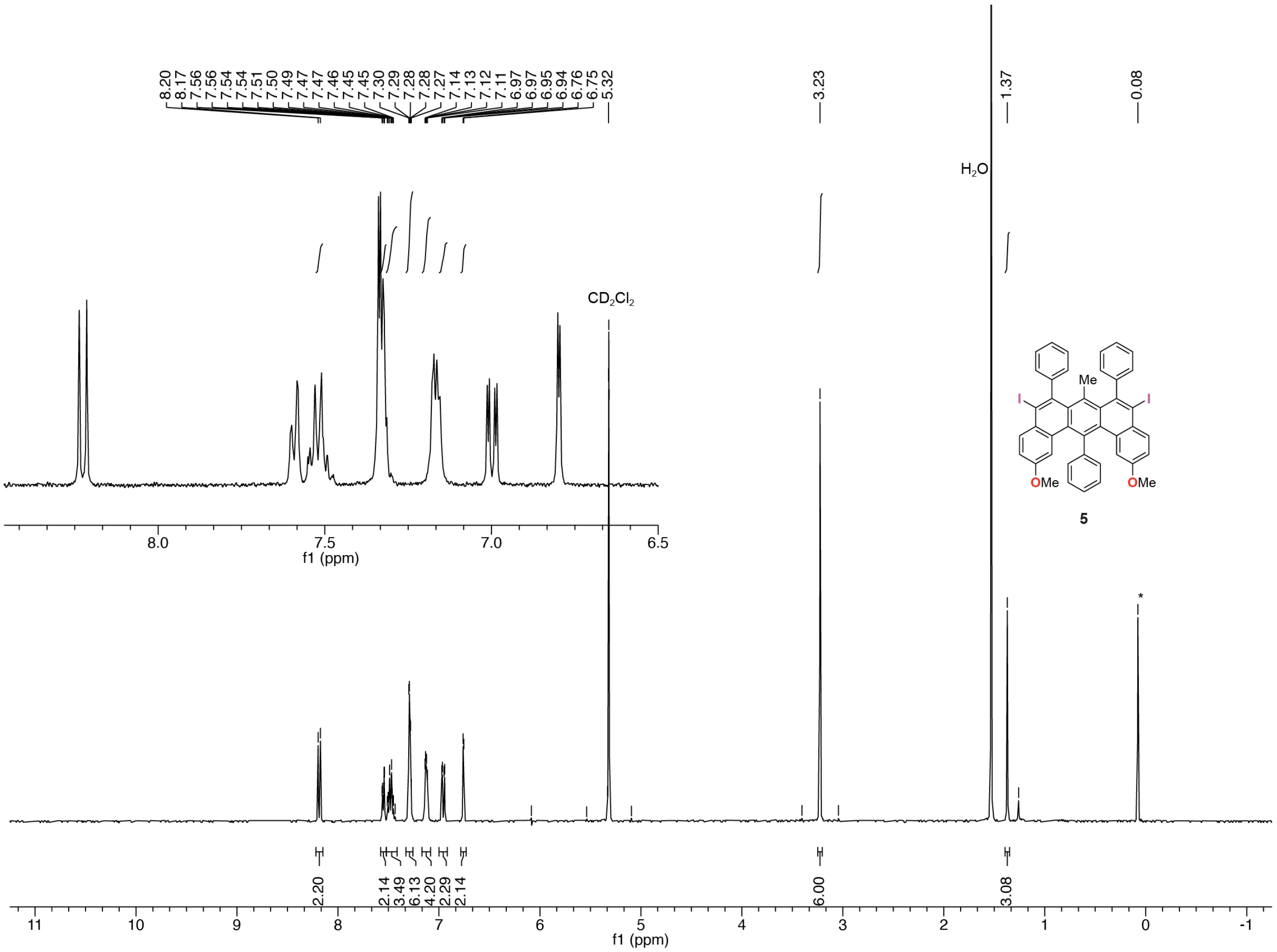

Figure S11. ^{1}H NMR (600 MHz, CD_2Cl_2) of 5,9-diiodo-2,12-dimethoxy-7-methyl-6,8,14-triphenylbenzo[m]tetraphene (**5**) at 24 °C (* silicon grease).

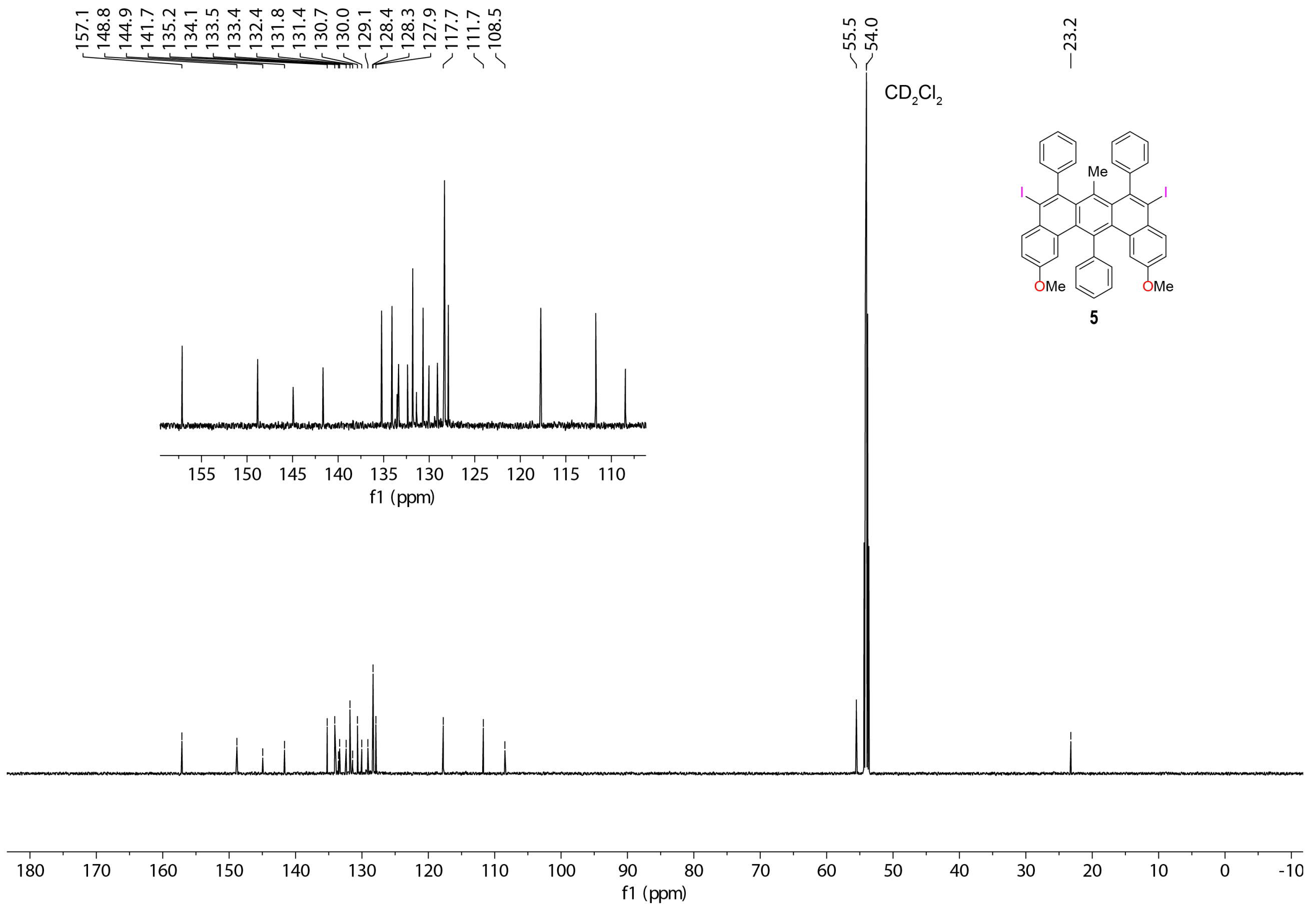

Figure S12. $^{13}C\{^1H\}$ NMR (151 MHz, CD_2Cl_2) spectrum of 5,9-diiodo-2,12-dimethoxy-7-methyl-6,8,14-triphenylbenzo[m]tetraphene (**5**) at 24 °C.

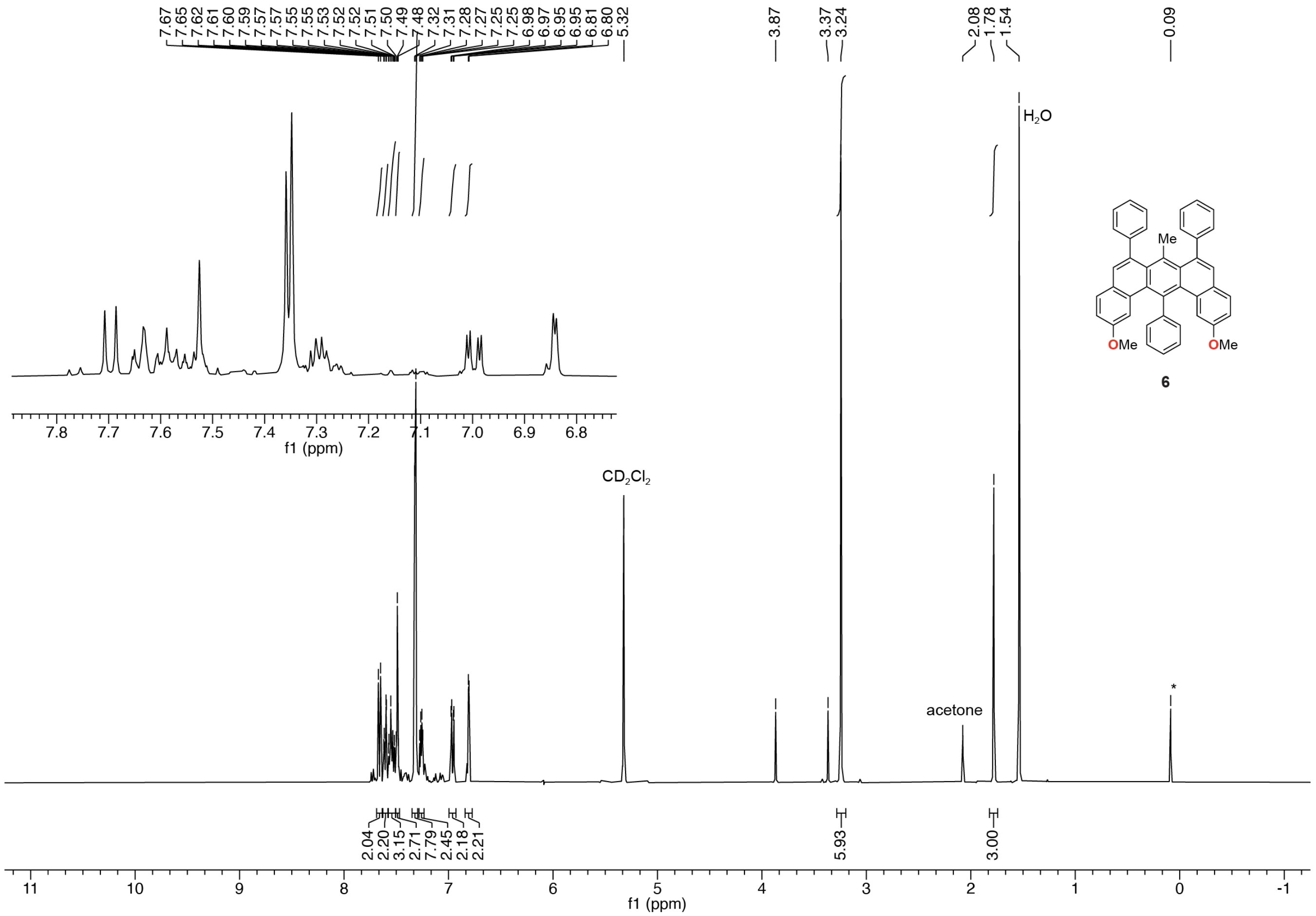

Figure S13. ^{1}H NMR (600 MHz, CD_2Cl_2) of 2,12-dimethoxy-7-methyl-6,8,14-triphenylbenzo[m]tetraphene (**6**) at 24 °C (* silicon grease).

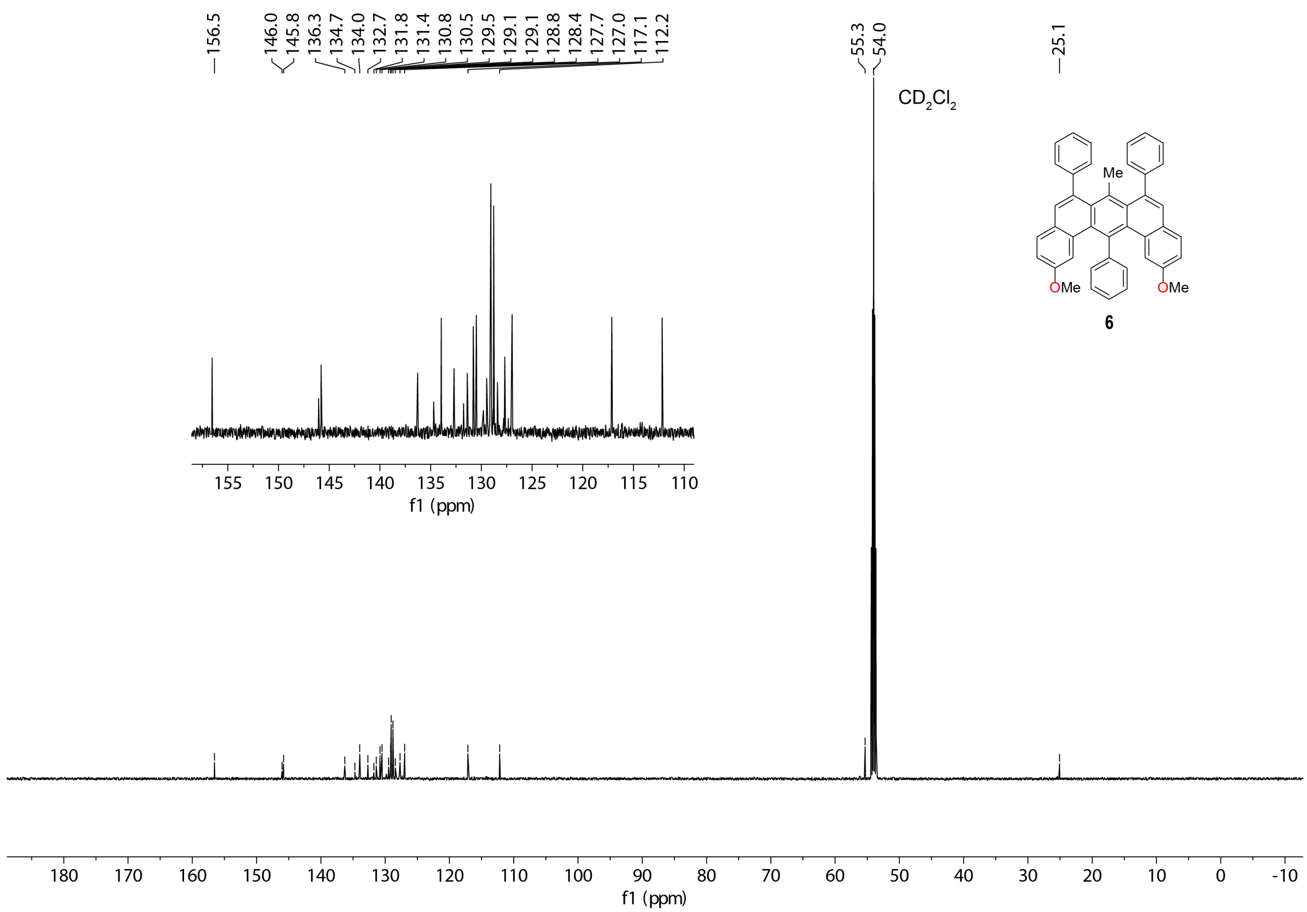

Figure S14. $^{13}C\{^1H\}$ NMR (151 MHz, CD_2Cl_2) spectrum of 2,12-dimethoxy-7-methyl-6,8,14-triphenylbenzo[m]tetraphene (**6**) at 24 °C.

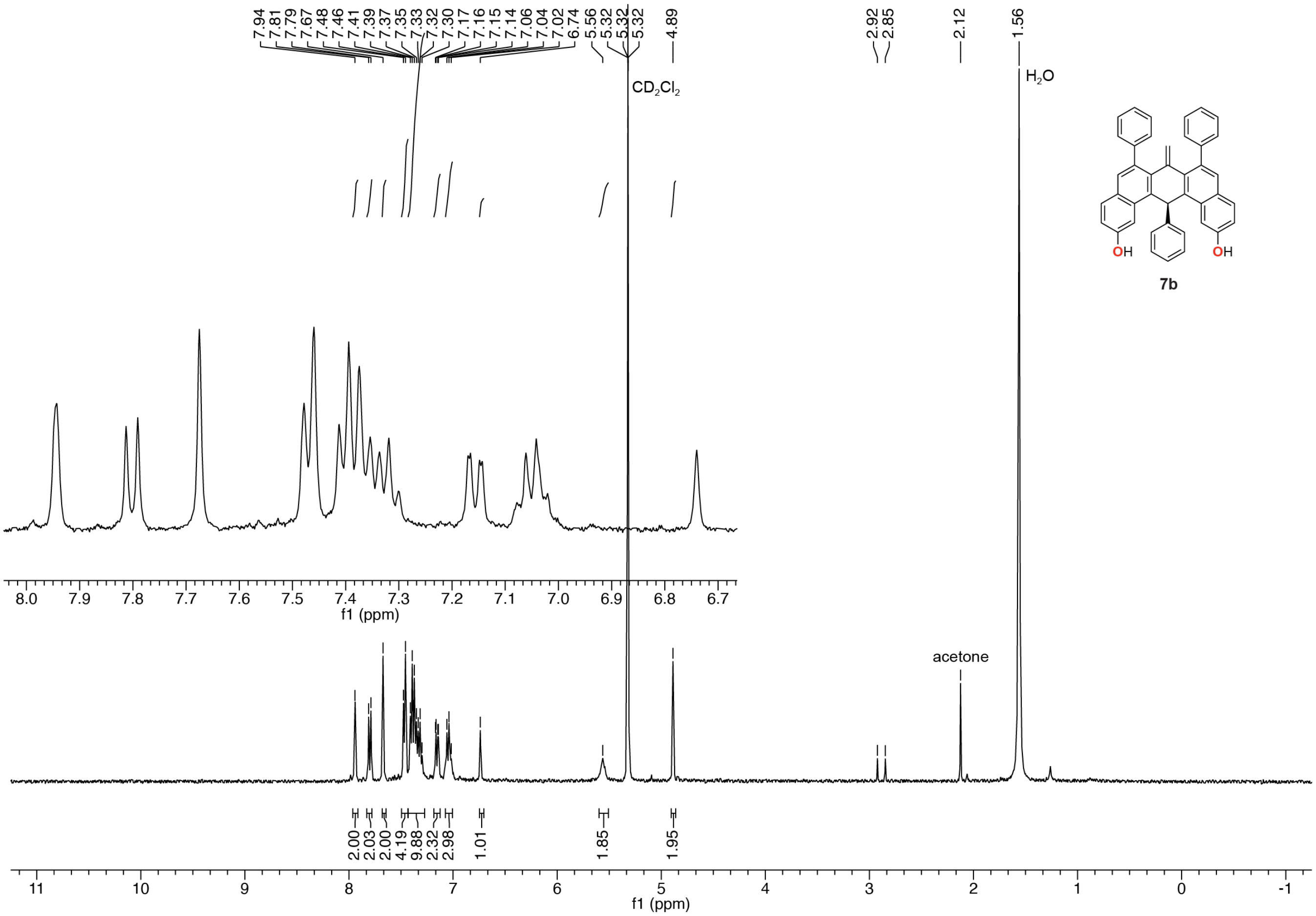

Figure S15. ^{1}H NMR (600 MHz, CD_2Cl_2) of 7-methylene-6,8,14-triphenyl-7,14-dihydrobenzo[m]tetraphene-2,12-diol (**7b**) at 24 °C.

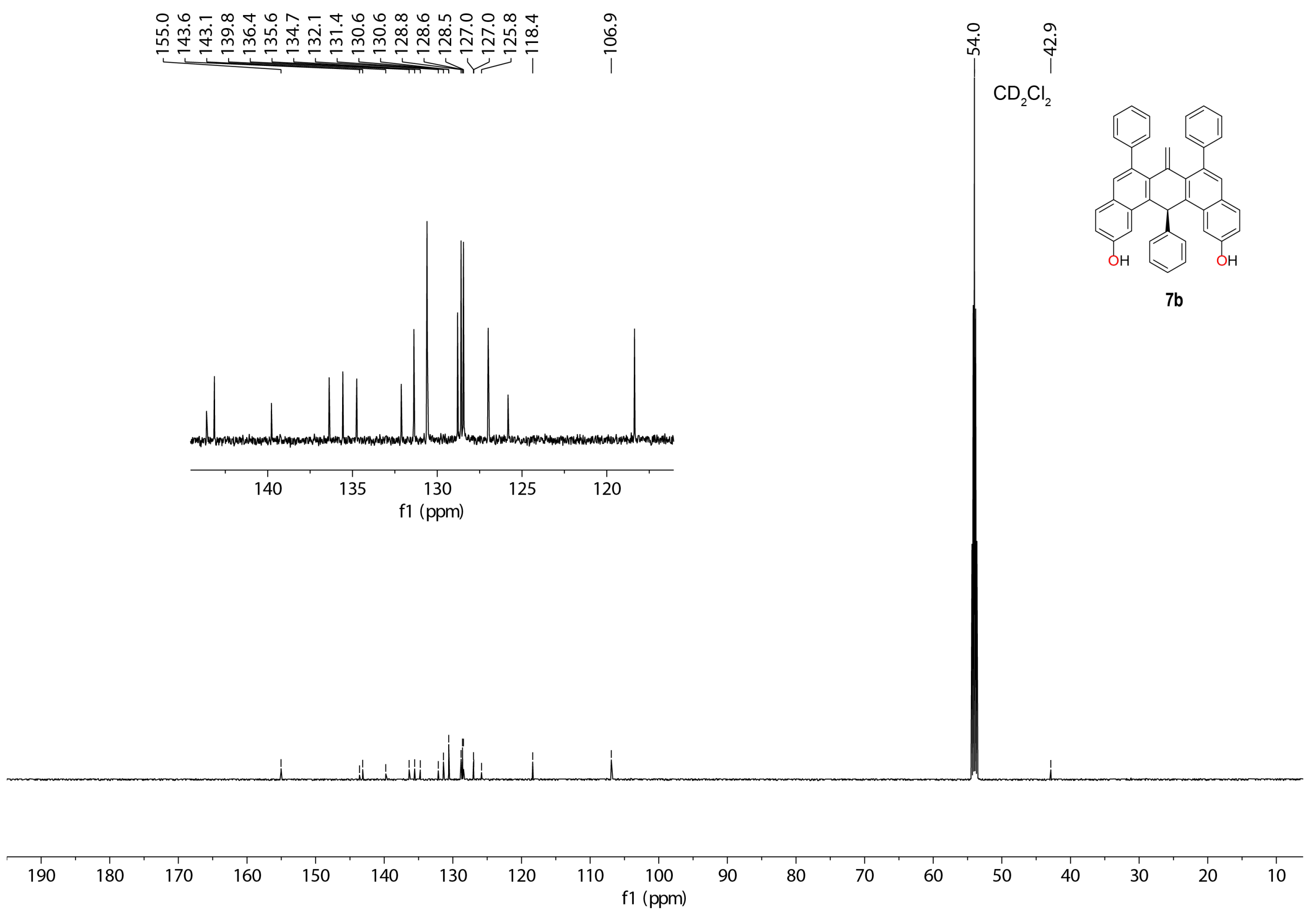

Figure S16. ^{13}C {^1H} NMR (151 MHz, CD_2Cl_2) spectrum of 7-methylene-6,8,14-triphenyl-7,14-dihydrobenzo[m]tetraphene-2,12-diol (**7b**) at 24 °C.

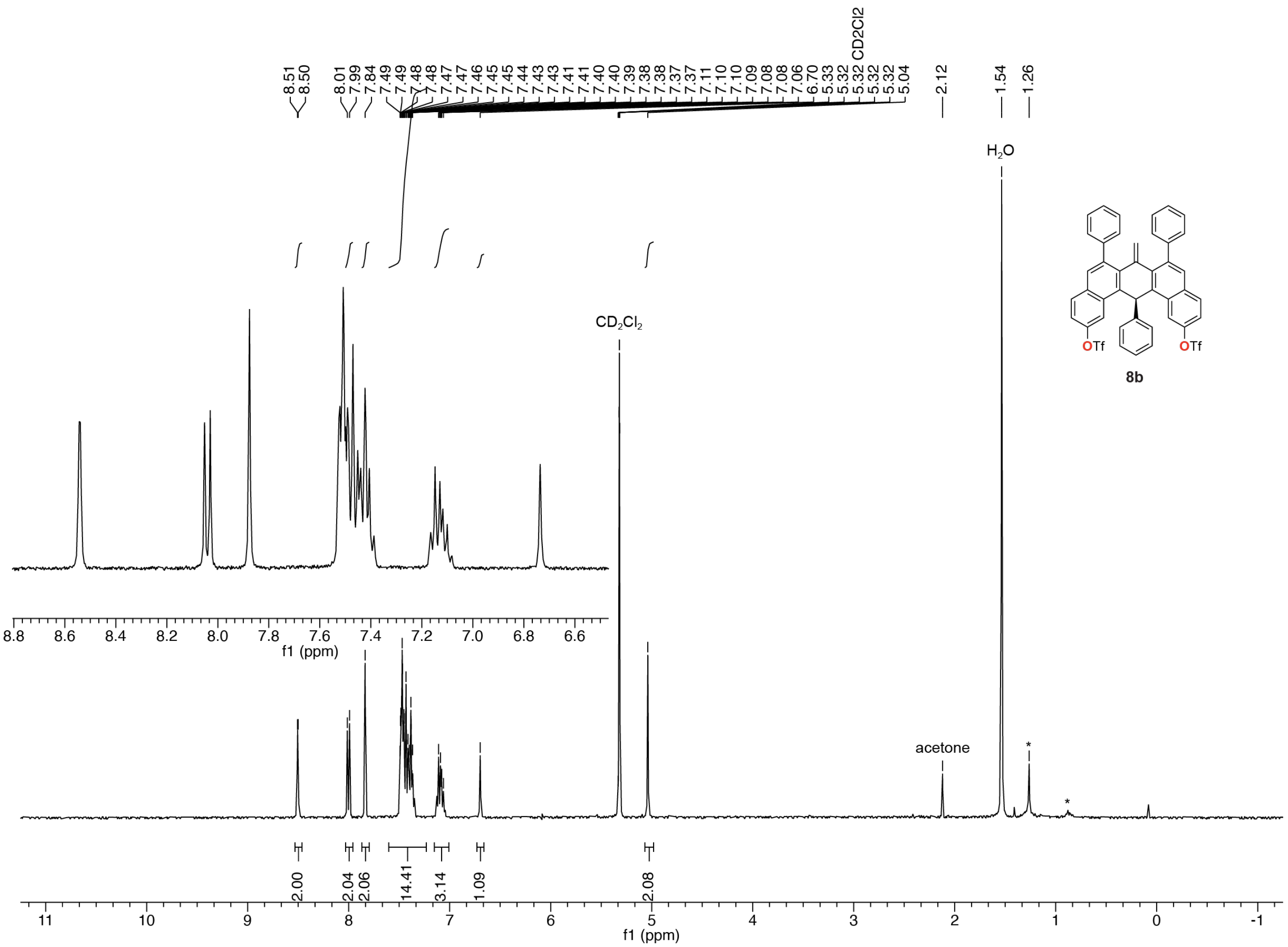

Figure S17. ^{1}H NMR (600 MHz, CD_2Cl_2) of 7-methylene-6,8,14-triphenyl-7,14-dihydrobenzo[m]tetraphene-2,12-diyl bis(trifluoromethanesulfonate) (**8b**) at 24 °C (* H grease).

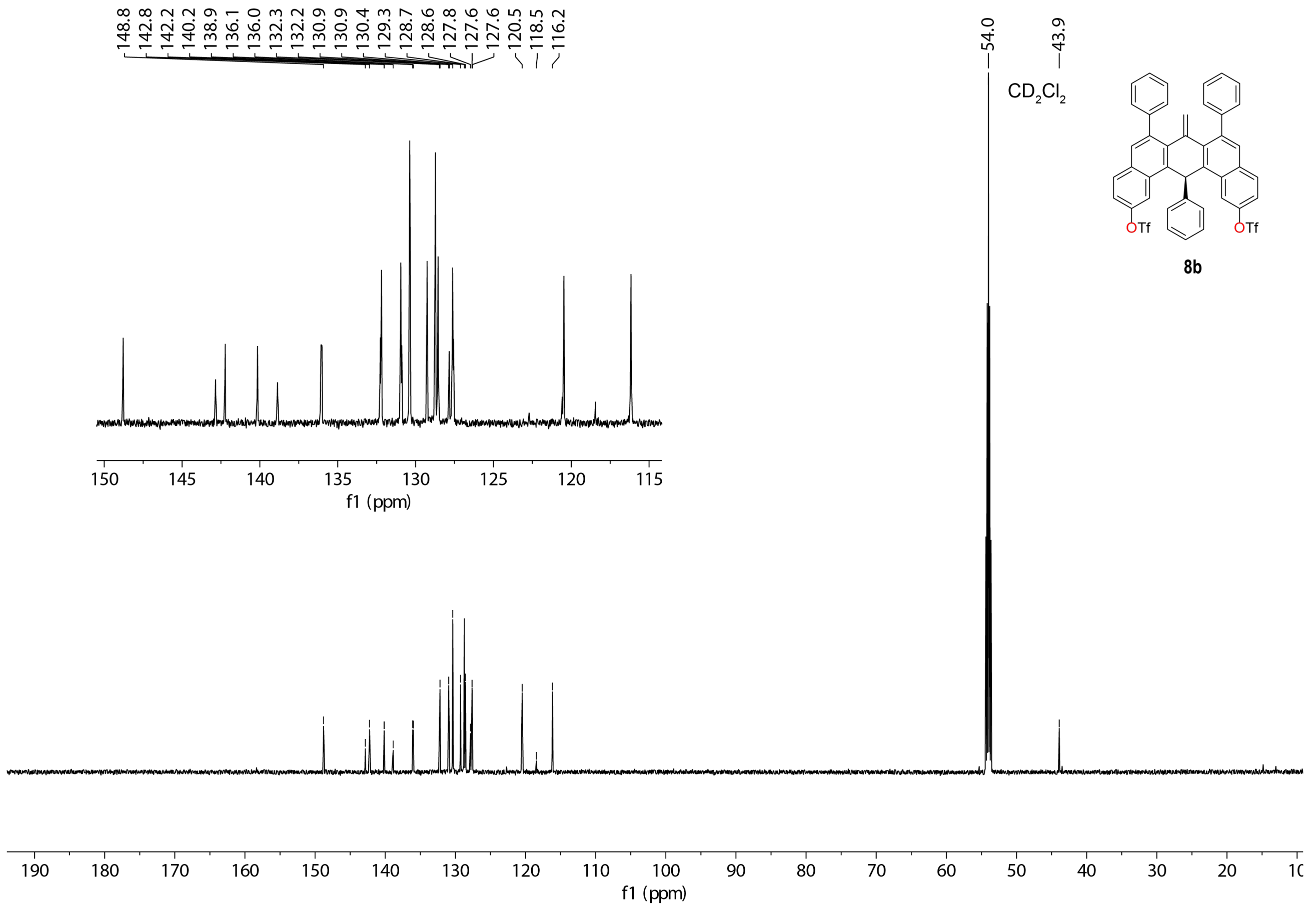

Figure S18. $^{13}C\{^{1}H\}$ NMR (151 MHz, CD_2Cl_2) spectrum of 7-methylene-6,8,14-triphenyl-7,14-dihydrobenzo[m]tetraphene-2,12-diyl bis(trifluoromethanesulfonate) (**8b**) at 24 °C.

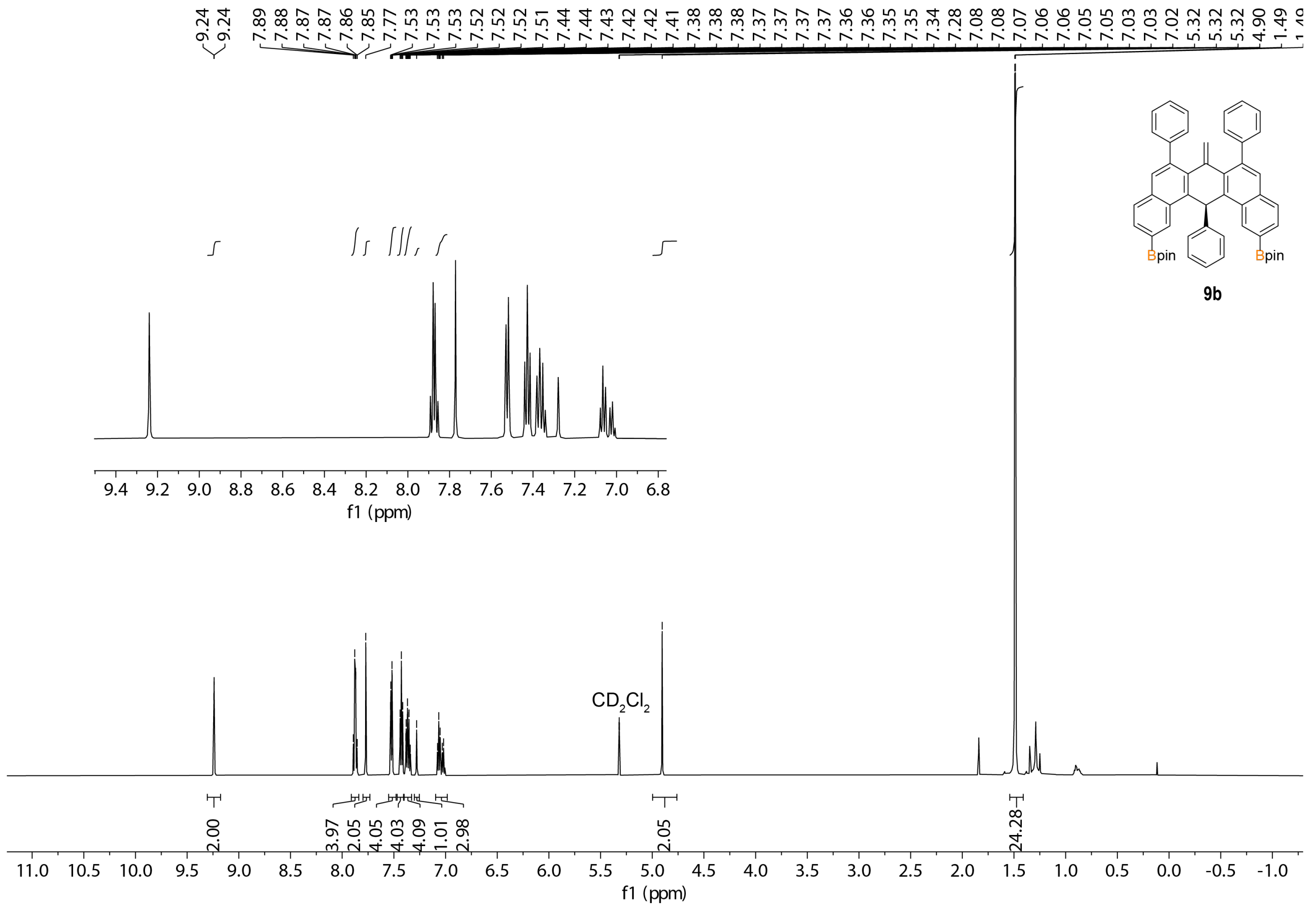

Figure S19. ^{1}H NMR (600 MHz, CD_2Cl_2) of 2,2'-(7-methylene-6,8,14-triphenyl-7,14-dihydrobenzo[m]tetraphene-2,12-diyl)bis(4,4,5,5-tetramethyl-1,3,2-dioxaborolane) (**9b**) at 24 °C.

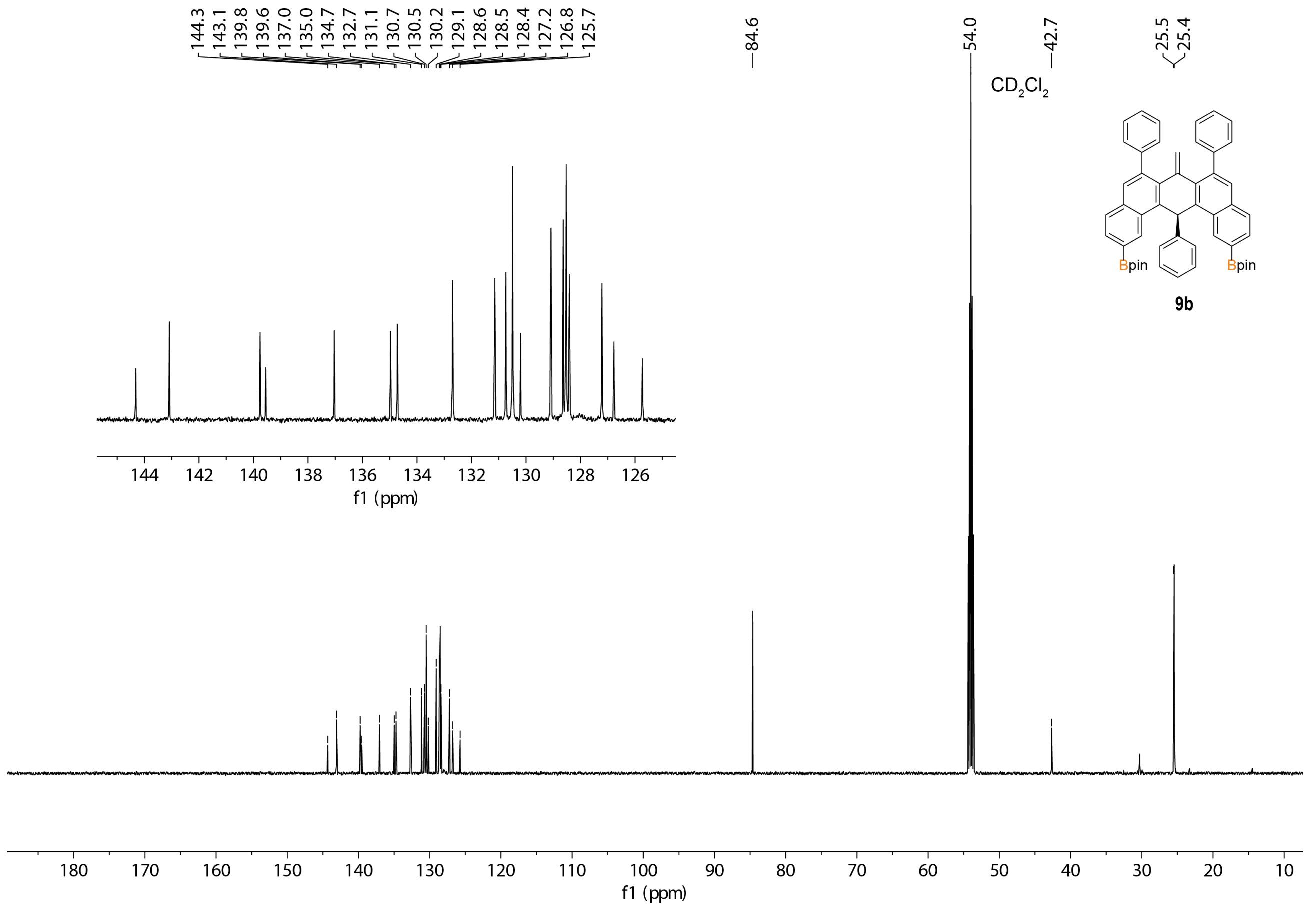

Figure S20. ^{13}C {^{1}H} NMR (151 MHz, CD_2Cl_2) spectrum of 2,2'-(7-methylene-6,8,14-triphenyl-7,14-dihydrobenzo[m]tetraphene-2,12-diyl)bis(4,4,5,5-tetramethyl-1,3,2-dioxaborolane) (**9b**) at 24 °C.

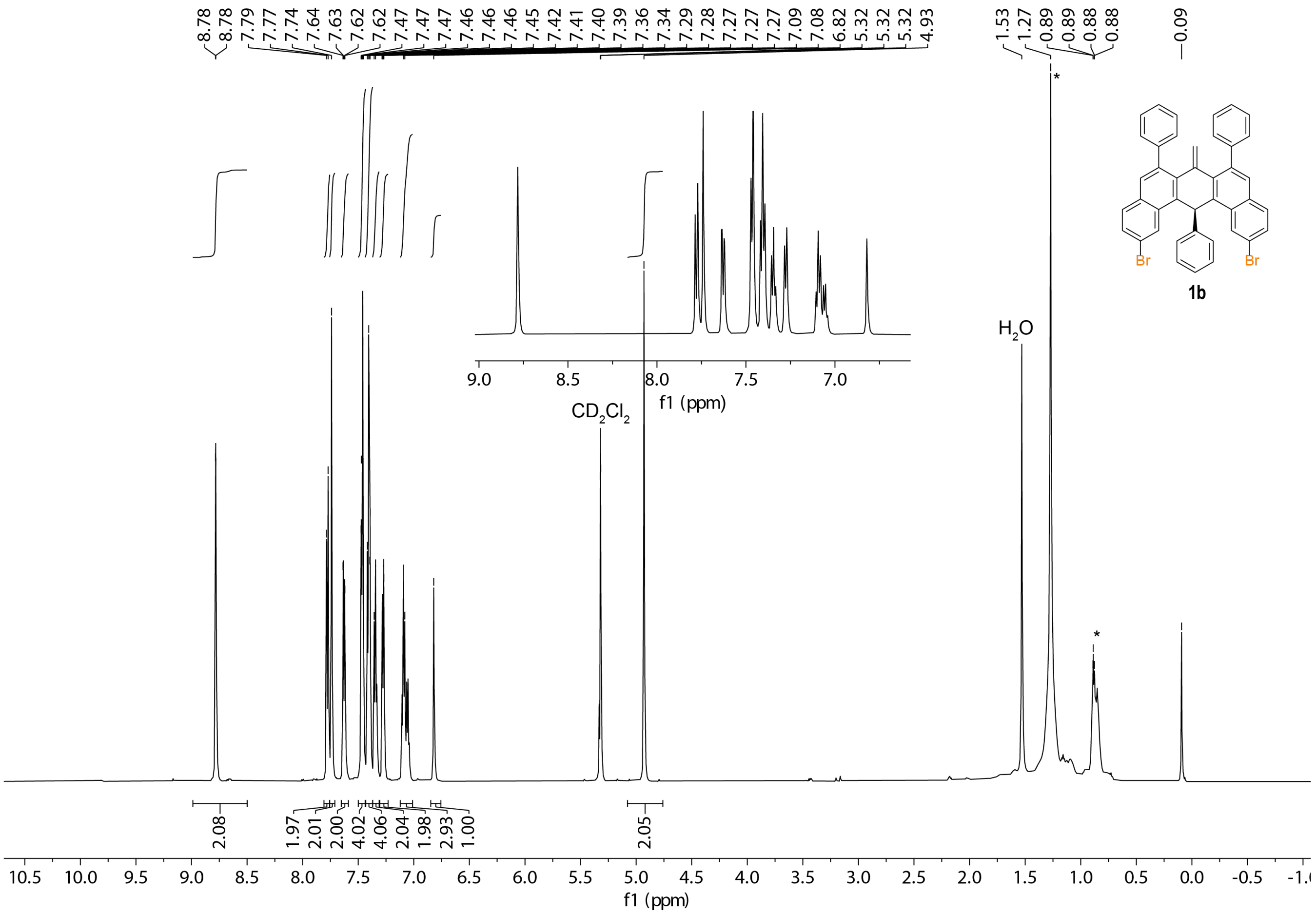

Figure S21. ^{1}H NMR (600 MHz, CD_2Cl_2) of 2,12-dibromo-7-methylene-6,8,14-triphenyl-7,14-dihydrobenzo[m]tetraphene (**1b**) at 24 °C (* H grease).

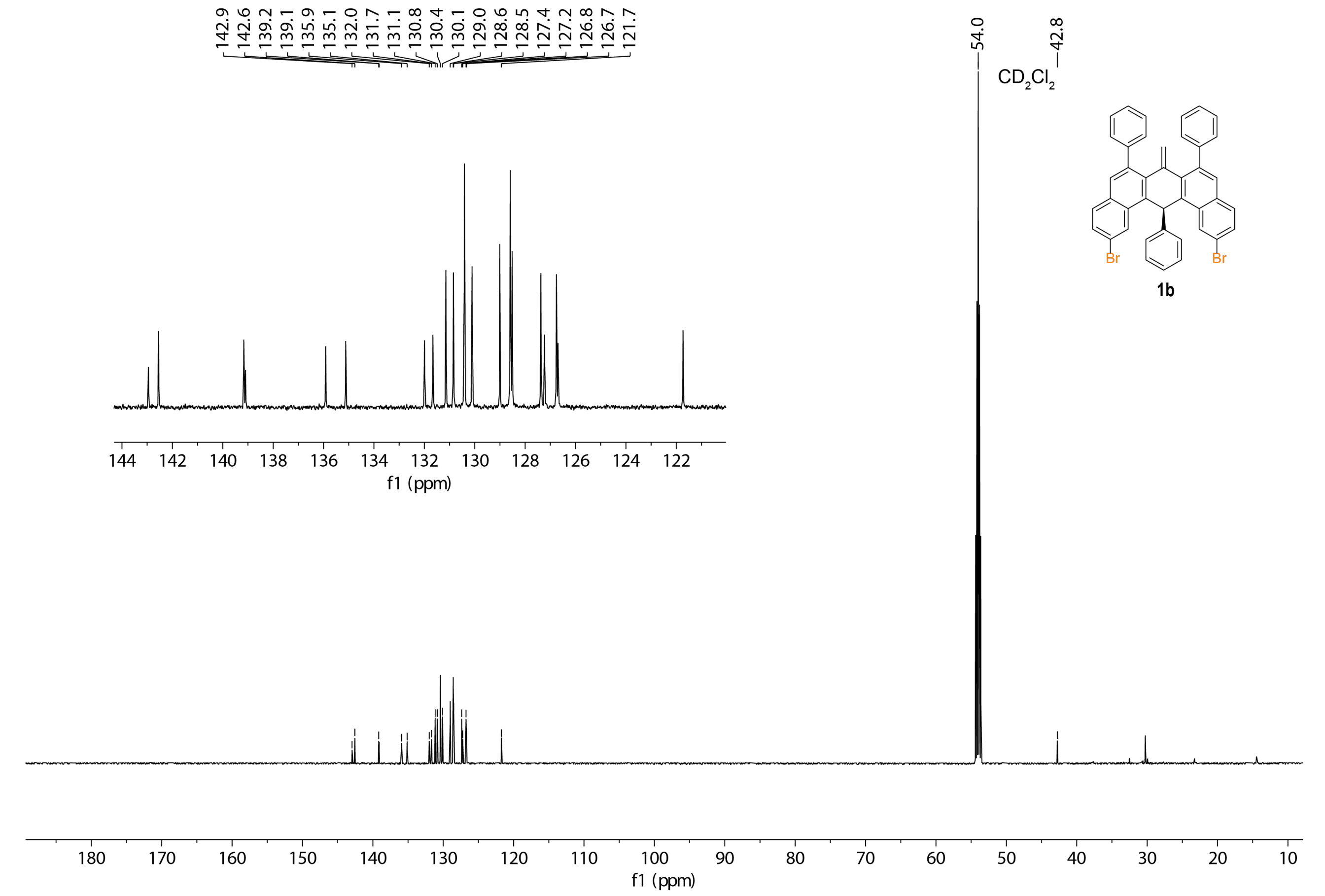

Figure S22. $^{13}C\{^1H\}$ NMR (151 MHz, CD_2Cl_2) spectrum of 2,12-dibromo-7-methylene-6,8,14-triphenyl-7,14-dihydrobenzo[m]tetraphene (**1b**) at 24 °C.

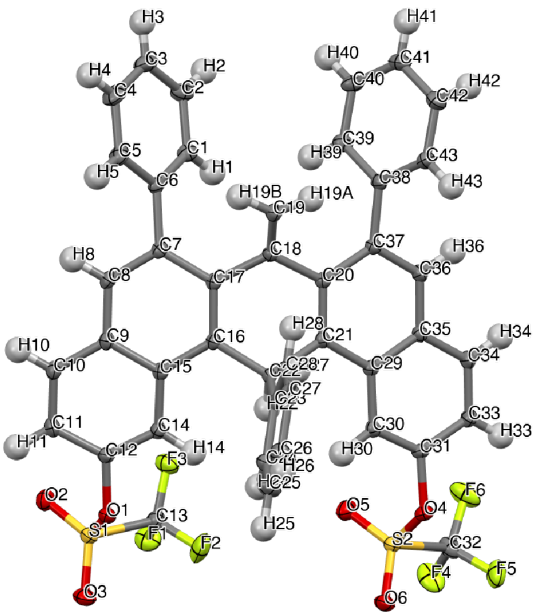

Figure S23. Single crystal X-ray structure diagram of 7-methylene-6,8,14-triphenyl-7,14-dihydrobenzo[m]tetraphene-2,12-diyl bis(trifluoromethanesulfonate) (**8b**). ORTEP thermal ellipsoids are at the 50% probability level. Color coding: C (gray), H (white), O (red), S (yellow), F (green).

Table S1. Crystal data and structure refinement for 7-methylene-6,8,14-triphenyl-7,14-dihydrobenzo[m]tetraphene-2,12-diyl bis(trifluoromethanesulfonate) (**8b**).

Identification code	CCDC 2130875	
Empirical formula	$C_{43} H_{26} F_6 O_6 S_2$	
Formula weight	816.76	
Temperature	100(2) K	
Wavelength	1.54184 Å	
Crystal system	Triclinic	
Space group	P –1	
Unit cell dimensions	a = 11.1512(2) Å	α = 66.5470(10)°.
	b = 12.8186(2) Å	β = 84.6210(10)°.
	c = 14.1810(2) Å	γ = 72.0490(10)°.
Volume	1768.11(5) $Å^3$	
Z	2	
Density (calculated)	1.534 Mg/m^3	
Absorption coefficient	2.108 mm^{-1}	
F(000)	836	
Crystal size	0.580 × 0.370 × 0.210 mm^3	
Theta range for data collection	3.399 to 74.501°	
Index ranges	$-13 \leq h \leq 13$, $-16 \leq k \leq 15$, $-17 \leq l \leq 12$	
Reflections collected	33619	
Independent reflections	7143 [R(int) = 0.0307]	
Completeness to theta = 74.000°	98.9 %	
Absorption correction	Semi–empirical from equivalents	
Max. and min. transmission	1.00000 and 0.72955	
Refinement method	Full–matrix least–squares on F^2	
Data / restraints / parameters	7143 / 0 / 514	
Goodness–of–fit on F^2	1.047	
Final R indices [I>2sigma(I)]	R1 = 0.0371, wR2 = 0.0958	
R indices (all data)	R1 = 0.0373, wR2 = 0.0959	
Extinction coefficient	n/a	
Largest diff. peak and hole	0.321 and –0.473 e $Å^{-3}$	

Table S2. Atomic coordinates (× 10^4) and equivalent isotropic displacement parameters ($Å^2$ × 10^3) for 7-methylene-6,8,14-triphenyl-7,14-dihydrobenzo[m]tetraphene-2,12-diyl bis(trifluoromethanesulfonate) (**8b**). U(eq) is defined as one third of the trace of the orthogonalized U^{ij} tensor.

	x	y	z	U(eq)
C(1)	5696(1)	4868(1)	6424(1)	20(1)
C(2)	5082(2)	4656(1)	7350(1)	24(1)
C(3)	5716(2)	4438(2)	8234(1)	29(1)
C(4)	6965(2)	4425(2)	8186(1)	31(1)
C(5)	7584(2)	4630(1)	7262(1)	23(1)
C(6)	6949(1)	4867(1)	6363(1)	16(1)
C(7)	7623(1)	5023(1)	5381(1)	15(1)
C(8)	8704(1)	4139(1)	5390(1)	17(1)
C(9)	9298(1)	4091(1)	4474(1)	16(1)
C(10)	10356(1)	3121(1)	4497(1)	19(1)
C(11)	10838(1)	2988(1)	3611(1)	18(1)
C(12)	10235(1)	3851(1)	2680(1)	16(1)
C(13)	8962(1)	3407(1)	875(1)	22(1)
C(14)	9260(1)	4833(1)	2605(1)	16(1)
C(15)	8756(1)	4989(1)	3515(1)	15(1)
C(16)	7708(1)	5976(1)	3512(1)	14(1)
C(17)	7151(1)	6005(1)	4418(1)	14(1)
C(18)	6113(1)	7100(1)	4355(1)	15(1)
C(19)	6008(1)	7564(1)	5066(1)	17(1)
C(20)	5303(1)	7708(1)	3396(1)	14(1)
C(21)	5864(1)	7674(1)	2490(1)	14(1)
C(22)	7257(1)	7029(1)	2500(1)	13(1)
C(23)	8094(1)	7852(1)	2246(1)	14(1)
C(24)	8965(1)	7890(1)	1468(1)	20(1)
C(25)	9745(2)	8617(2)	1248(1)	25(1)
C(26)	9667(1)	9318(1)	1800(1)	23(1)
C(27)	8802(1)	9288(1)	2575(1)	21(1)
C(28)	8021(1)	8562(1)	2795(1)	18(1)
C(29)	5148(1)	8224(1)	1541(1)	14(1)
C(30)	5672(1)	8216(1)	587(1)	15(1)
C(31)	4898(1)	8729(1)	−265(1)	15(1)
C(32)	4361(2)	8072(2)	−2325(1)	25(1)
C(33)	3601(1)	9291(1)	−277(1)	18(1)
C(34)	3087(1)	9325(1)	627(1)	18(1)
C(35)	3838(1)	8804(1)	1546(1)	16(1)
C(36)	3298(1)	8851(1)	2474(1)	18(1)
C(37)	3985(1)	8326(1)	3383(1)	15(1)
C(38)	3256(1)	8420(1)	4302(1)	16(1)
C(39)	3108(1)	7417(1)	5122(1)	19(1)
C(40)	2323(2)	7536(1)	5914(1)	22(1)
C(41)	1662(2)	8651(2)	5896(1)	23(1)
C(42)	1802(2)	9655(2)	5085(1)	29(1)
C(43)	2598(2)	9542(1)	4293(1)	25(1)
F(1)	8746(1)	2675(1)	503(1)	33(1)
F(2)	8803(1)	4464(1)	136(1)	39(1)
F(3)	8151(1)	3497(1)	1601(1)	28(1)
F(4)	4543(1)	7275(1)	−2734(1)	39(1)
F(5)	4148(1)	9144(1)	−3065(1)	36(1)
F(6)	3370(1)	8046(1)	−1736(1)	38(1)
O(1)	10708(1)	3770(1)	1728(1)	17(1)
O(2)	10630(1)	1699(1)	2247(1)	23(1)
O(3)	11368(1)	2820(1)	544(1)	26(1)
O(4)	5447(1)	8802(1)	−1236(1)	17(1)
O(5)	5886(1)	6634(1)	−717(1)	28(1)
O(6)	6727(1)	7880(1)	−2307(1)	24(1)
S(1)	10590(1)	2777(1)	1399(1)	18(1)
S(2)	5783(1)	7717(1)	−1570(1)	17(1)

Table S3. Bond lengths [Å] and angles [°] for 7-methylene-6,8,14-triphenyl-7,14-dihydrobenzo[m]tetraphene-2,12-diyl bis(trifluoromethanesulfonate) (**8b**).

C(1)–C(2)	1.389(2)
C(1)–C(6)	1.391(2)
C(1)–H(1)	0.9500
C(2)–C(3)	1.384(2)
C(2)–H(2)	0.9500
C(3)–C(4)	1.384(3)
C(3)–H(3)	0.9500
C(4)–C(5)	1.389(2)
C(4)–H(4)	0.9500
C(5)–C(6)	1.395(2)
C(5)–H(5)	0.9500
C(6)–C(7)	1.4901(19)
C(7)–C(8)	1.374(2)
C(7)–C(17)	1.4435(19)
C(8)–C(9)	1.4163(19)
C(8)–H(8)	0.9500
C(9)–C(10)	1.419(2)
C(9)–C(15)	1.4213(19)
C(10)–C(11)	1.372(2)
C(10)–H(10)	0.9500
C(11)–C(12)	1.402(2)
C(11)–H(11)	0.9500
C(12)–C(14)	1.360(2)
C(12)–O(1)	1.4340(16)
C(13)–F(2)	1.3128(18)
C(13)–F(3)	1.3212(18)
C(13)–F(1)	1.3316(19)
C(13)–S(1)	1.8362(16)
C(14)–C(15)	1.4218(19)
C(14)–H(14)	0.9500
C(15)–C(16)	1.4326(19)
C(16)–C(17)	1.3819(19)
C(16)–C(22)	1.5194(18)
C(17)–C(18)	1.4956(19)
C(18)–C(19)	1.341(2)
C(18)–C(20)	1.4916(18)
C(19)–H(19A)	0.9500
C(19)–H(19B)	0.9500
C(20)–C(21)	1.3863(19)
C(20)–C(37)	1.4361(19)
C(21)–C(29)	1.4304(18)
C(21)–C(22)	1.5169(18)
C(22)–C(23)	1.5354(18)
C(22)–H(22)	1.0000
C(23)–C(28)	1.395(2)
C(23)–C(24)	1.3955(19)
C(24)–C(25)	1.394(2)
C(24)–H(24)	0.9500
C(25)–C(26)	1.387(2)
C(25)–H(25)	0.9500
C(26)–C(27)	1.389(2)
C(26)–H(26)	0.9500
C(27)–C(28)	1.393(2)
C(27)–H(27)	0.9500
C(28)–H(28)	0.9500
C(29)–C(35)	1.4212(19)
C(29)–C(30)	1.4243(19)
C(30)–C(31)	1.3607(19)
C(30)–H(30)	0.9500
C(31)–C(33)	1.401(2)

C(31)–O(4)	1.4334(16)
C(32)–F(4)	1.3197(19)
C(32)–F(5)	1.320(2)
C(32)–F(6)	1.3216(19)
C(32)–S(2)	1.8316(16)
C(33)–C(34)	1.365(2)
C(33)–H(33)	0.9500
C(34)–C(35)	1.4188(19)
C(34)–H(34)	0.9500
C(35)–C(36)	1.4104(19)
C(36)–C(37)	1.371(2)
C(36)–H(36)	0.9500
C(37)–C(38)	1.4970(18)
C(38)–C(39)	1.392(2)
C(38)–C(43)	1.395(2)
C(39)–C(40)	1.386(2)
C(39)–H(39)	0.9500
C(40)–C(41)	1.383(2)
C(40)–H(40)	0.9500
C(41)–C(42)	1.383(2)
C(41)–H(41)	0.9500
C(42)–C(43)	1.391(2)
C(42)–H(42)	0.9500
C(43)–H(43)	0.9500
O(1)–S(1)	1.5632(10)
O(2)–S(1)	1.4169(11)
O(3)–S(1)	1.4163(11)
O(4)–S(2)	1.5708(10)
O(5)–S(2)	1.4128(11)
O(6)–S(2)	1.4148(11)

C(2)–C(1)–C(6)	121.14(14)
C(2)–C(1)–H(1)	119.4
C(6)–C(1)–H(1)	119.4
C(3)–C(2)–C(1)	120.07(15)
C(3)–C(2)–H(2)	120.0
C(1)–C(2)–H(2)	120.0
C(2)–C(3)–C(4)	119.34(15)
C(2)–C(3)–H(3)	120.3
C(4)–C(3)–H(3)	120.3
C(3)–C(4)–C(5)	120.70(15)
C(3)–C(4)–H(4)	119.6
C(5)–C(4)–H(4)	119.6
C(4)–C(5)–C(6)	120.42(15)
C(4)–C(5)–H(5)	119.8
C(6)–C(5)–H(5)	119.8
C(1)–C(6)–C(5)	118.30(13)
C(1)–C(6)–C(7)	120.76(13)
C(5)–C(6)–C(7)	120.76(13)
C(8)–C(7)–C(17)	118.95(13)
C(8)–C(7)–C(6)	117.52(12)
C(17)–C(7)–C(6)	123.38(12)
C(7)–C(8)–C(9)	122.15(13)
C(7)–C(8)–H(8)	118.9
C(9)–C(8)–H(8)	118.9
C(8)–C(9)–C(10)	121.20(13)
C(8)–C(9)–C(15)	118.84(13)
C(10)–C(9)–C(15)	119.78(13)
C(11)–C(10)–C(9)	121.62(13)
C(11)–C(10)–H(10)	119.2
C(9)–C(10)–H(10)	119.2
C(10)–C(11)–C(12)	117.14(13)
C(10)–C(11)–H(11)	121.4
C(12)–C(11)–H(11)	121.4

C(14)–C(12)–C(11)	123.98(13)
C(14)–C(12)–O(1)	116.18(12)
C(11)–C(12)–O(1)	119.67(12)
F(2)–C(13)–F(3)	109.14(13)
F(2)–C(13)–F(1)	109.49(13)
F(3)–C(13)–F(1)	108.86(12)
F(2)–C(13)–S(1)	110.56(10)
F(3)–C(13)–S(1)	110.89(10)
F(1)–C(13)–S(1)	107.87(11)
C(12)–C(14)–C(15)	119.49(13)
C(12)–C(14)–H(14)	120.3
C(15)–C(14)–H(14)	120.3
C(9)–C(15)–C(14)	117.79(12)
C(9)–C(15)–C(16)	118.73(12)
C(14)–C(15)–C(16)	123.44(12)
C(17)–C(16)–C(15)	120.97(12)
C(17)–C(16)–C(22)	120.10(12)
C(15)–C(16)–C(22)	118.85(12)
C(16)–C(17)–C(7)	119.68(12)
C(16)–C(17)–C(18)	117.56(12)
C(7)–C(17)–C(18)	122.69(12)
C(19)–C(18)–C(20)	122.51(13)
C(19)–C(18)–C(17)	121.65(12)
C(20)–C(18)–C(17)	115.49(12)
C(18)–C(19)–H(19A)	120.0
C(18)–C(19)–H(19B)	120.0
H(19A)–C(19)–H(19B)	120.0
C(21)–C(20)–C(37)	119.69(12)
C(21)–C(20)–C(18)	117.79(12)
C(37)–C(20)–C(18)	122.52(12)
C(20)–C(21)–C(29)	121.17(12)
C(20)–C(21)–C(22)	119.80(12)
C(29)–C(21)–C(22)	119.02(12)
C(21)–C(22)–C(16)	112.28(11)
C(21)–C(22)–C(23)	113.05(11)
C(16)–C(22)–C(23)	109.88(11)
C(21)–C(22)–H(22)	107.1
C(16)–C(22)–H(22)	107.1
C(23)–C(22)–H(22)	107.1
C(28)–C(23)–C(24)	118.53(13)
C(28)–C(23)–C(22)	121.06(12)
C(24)–C(23)–C(22)	120.40(12)
C(25)–C(24)–C(23)	120.62(14)
C(25)–C(24)–H(24)	119.7
C(23)–C(24)–H(24)	119.7
C(26)–C(25)–C(24)	120.41(14)
C(26)–C(25)–H(25)	119.8
C(24)–C(25)–H(25)	119.8
C(25)–C(26)–C(27)	119.38(14)
C(25)–C(26)–H(26)	120.3
C(27)–C(26)–H(26)	120.3
C(26)–C(27)–C(28)	120.30(14)
C(26)–C(27)–H(27)	119.8
C(28)–C(27)–H(27)	119.8
C(27)–C(28)–C(23)	120.76(13)
C(27)–C(28)–H(28)	119.6
C(23)–C(28)–H(28)	119.6
C(35)–C(29)–C(30)	117.90(12)
C(35)–C(29)–C(21)	118.43(12)
C(30)–C(29)–C(21)	123.66(12)
C(31)–C(30)–C(29)	118.96(12)
C(31)–C(30)–H(30)	120.5
C(29)–C(30)–H(30)	120.5
C(30)–C(31)–C(33)	124.21(13)

C(30)–C(31)–O(4)	118.59(12)
C(33)–C(31)–O(4)	116.95(12)
F(4)–C(32)–F(5)	109.44(14)
F(4)–C(32)–F(6)	109.60(14)
F(5)–C(32)–F(6)	108.83(13)
F(4)–C(32)–S(2)	108.32(11)
F(5)–C(32)–S(2)	109.97(11)
F(6)–C(32)–S(2)	110.66(11)
C(34)–C(33)–C(31)	117.59(13)
C(34)–C(33)–H(33)	121.2
C(31)–C(33)–H(33)	121.2
C(33)–C(34)–C(35)	121.24(13)
C(33)–C(34)–H(34)	119.4
C(35)–C(34)–H(34)	119.4
C(36)–C(35)–C(34)	120.72(13)
C(36)–C(35)–C(29)	119.20(12)
C(34)–C(35)–C(29)	120.08(13)
C(37)–C(36)–C(35)	122.31(13)
C(37)–C(36)–H(36)	118.8
C(35)–C(36)–H(36)	118.8
C(36)–C(37)–C(20)	119.16(13)
C(36)–C(37)–C(38)	115.43(12)
C(20)–C(37)–C(38)	125.38(12)
C(39)–C(38)–C(43)	118.74(13)
C(39)–C(38)–C(37)	121.57(13)
C(43)–C(38)–C(37)	119.34(13)
C(40)–C(39)–C(38)	120.42(14)
C(40)–C(39)–H(39)	119.8
C(38)–C(39)–H(39)	119.8
C(41)–C(40)–C(39)	120.62(14)
C(41)–C(40)–H(40)	119.7
C(39)–C(40)–H(40)	119.7
C(40)–C(41)–C(42)	119.49(14)
C(40)–C(41)–H(41)	120.3
C(42)–C(41)–H(41)	120.3
C(41)–C(42)–C(43)	120.20(15)
C(41)–C(42)–H(42)	119.9
C(43)–C(42)–H(42)	119.9
C(42)–C(43)–C(38)	120.53(14)
C(42)–C(43)–H(43)	119.7
C(38)–C(43)–H(43)	119.7
C(12)–O(1)–S(1)	121.94(9)
C(31)–O(4)–S(2)	121.25(9)
O(3)–S(1)–O(2)	122.53(7)
O(3)–S(1)–O(1)	106.29(6)
O(2)–S(1)–O(1)	112.89(6)
O(3)–S(1)–C(13)	105.71(7)
O(2)–S(1)–C(13)	106.02(7)
O(1)–S(1)–C(13)	101.08(6)
O(5)–S(2)–O(6)	123.06(7)
O(5)–S(2)–O(4)	111.76(6)
O(6)–S(2)–O(4)	106.38(6)
O(5)–S(2)–C(32)	107.90(8)
O(6)–S(2)–C(32)	104.26(7)
O(4)–S(2)–C(32)	101.08(6)

Symmetry transformations used to generate equivalent atoms:

Table S4. Anisotropic displacement parameters ($Å^2 \times 10^3$) for 7-methylene-6,8,14-triphenyl-7,14-dihydrobenzo[m]tetraphene-2,12-diyl bis(trifluoromethanesulfonate) (**8b**). The anisotropic displacement factor exponent takes the form: $-2p^2 [h^2a^{*2}U^{11} + ... + 2 h k a^* b^* U^{12}]$

	U^{11}	U^{22}	U^{33}	U^{23}	U^{13}	U^{12}
C(1)	22(1)	20(1)	18(1)	−9(1)	1(1)	−8(1)
C(2)	28(1)	22(1)	25(1)	−10(1)	8(1)	−13(1)
C(3)	41(1)	26(1)	17(1)	−7(1)	9(1)	−12(1)
C(4)	40(1)	38(1)	15(1)	−9(1)	−4(1)	−10(1)
C(5)	25(1)	27(1)	18(1)	−8(1)	−4(1)	−7(1)
C(6)	21(1)	13(1)	14(1)	−5(1)	0(1)	−5(1)
C(7)	17(1)	16(1)	15(1)	−6(1)	−1(1)	−6(1)
C(8)	19(1)	17(1)	14(1)	−4(1)	−3(1)	−5(1)
C(9)	16(1)	16(1)	17(1)	−6(1)	−2(1)	−4(1)
C(10)	18(1)	17(1)	17(1)	−4(1)	−3(1)	−2(1)
C(11)	15(1)	16(1)	21(1)	−7(1)	−1(1)	−1(1)
C(12)	15(1)	18(1)	15(1)	−7(1)	2(1)	−5(1)
C(13)	20(1)	24(1)	19(1)	−6(1)	0(1)	−7(1)
C(14)	15(1)	15(1)	15(1)	−4(1)	0(1)	−5(1)
C(15)	14(1)	15(1)	16(1)	−6(1)	−1(1)	−5(1)
C(16)	13(1)	14(1)	15(1)	−5(1)	−2(1)	−4(1)
C(17)	14(1)	15(1)	15(1)	−6(1)	−1(1)	−5(1)
C(18)	13(1)	16(1)	14(1)	−4(1)	1(1)	−5(1)
C(19)	16(1)	18(1)	16(1)	−6(1)	0(1)	−4(1)
C(20)	14(1)	14(1)	14(1)	−5(1)	0(1)	−4(1)
C(21)	13(1)	13(1)	15(1)	−5(1)	0(1)	−4(1)
C(22)	13(1)	13(1)	13(1)	−5(1)	0(1)	−2(1)
C(23)	12(1)	14(1)	13(1)	−2(1)	−3(1)	−1(1)
C(24)	20(1)	21(1)	22(1)	−10(1)	5(1)	−6(1)
C(25)	22(1)	27(1)	30(1)	−13(1)	11(1)	−11(1)
C(26)	18(1)	23(1)	29(1)	−8(1)	2(1)	−10(1)
C(27)	23(1)	21(1)	21(1)	−8(1)	−2(1)	−7(1)
C(28)	18(1)	19(1)	15(1)	−6(1)	1(1)	−6(1)
C(29)	14(1)	13(1)	14(1)	−5(1)	1(1)	−5(1)
C(30)	13(1)	14(1)	16(1)	−6(1)	0(1)	−4(1)
C(31)	17(1)	16(1)	12(1)	−5(1)	3(1)	−6(1)
C(32)	20(1)	30(1)	31(1)	−19(1)	1(1)	−5(1)
C(33)	17(1)	19(1)	16(1)	−5(1)	−3(1)	−3(1)
C(34)	14(1)	20(1)	18(1)	−6(1)	−2(1)	−2(1)
C(35)	13(1)	16(1)	16(1)	−5(1)	0(1)	−4(1)
C(36)	13(1)	20(1)	18(1)	−7(1)	0(1)	−2(1)
C(37)	15(1)	15(1)	15(1)	−6(1)	1(1)	−4(1)
C(38)	12(1)	21(1)	15(1)	−7(1)	0(1)	−3(1)
C(39)	21(1)	20(1)	20(1)	−10(1)	3(1)	−8(1)
C(40)	26(1)	25(1)	18(1)	−9(1)	5(1)	−15(1)
C(41)	22(1)	31(1)	20(1)	−13(1)	6(1)	−10(1)
C(42)	32(1)	23(1)	25(1)	−11(1)	8(1)	1(1)
C(43)	29(1)	20(1)	18(1)	−4(1)	6(1)	−2(1)
F(1)	31(1)	46(1)	32(1)	−22(1)	−3(1)	−13(1)
F(2)	32(1)	34(1)	34(1)	9(1)	−12(1)	−12(1)
F(3)	19(1)	40(1)	29(1)	−17(1)	3(1)	−9(1)
F(4)	37(1)	45(1)	51(1)	−37(1)	−7(1)	−5(1)
F(5)	32(1)	34(1)	32(1)	−10(1)	−14(1)	1(1)
F(6)	21(1)	56(1)	54(1)	−36(1)	9(1)	−17(1)
O(1)	16(1)	18(1)	16(1)	−7(1)	3(1)	−3(1)
O(2)	26(1)	18(1)	23(1)	−7(1)	−2(1)	−4(1)
O(3)	23(1)	35(1)	22(1)	−16(1)	6(1)	−7(1)
O(4)	19(1)	19(1)	13(1)	−6(1)	2(1)	−7(1)
O(5)	39(1)	18(1)	22(1)	−7(1)	3(1)	−4(1)
O(6)	18(1)	34(1)	20(1)	−12(1)	4(1)	−4(1)
S(1)	17(1)	19(1)	16(1)	−8(1)	1(1)	−3(1)
S(2)	16(1)	19(1)	15(1)	−7(1)	1(1)	3(1)

Table S5. Hydrogen coordinates (× 10^4) and isotropic displacement parameters ($Å^2$ × 10^3) for 7-methylene-6,8,14-triphenyl-7,14-dihydrobenzo[m]tetraphene-2,12-diyl bis(trifluoromethanesulfonate) (**8b**).

	x	y	z	U(eq)
H(1)	5253	5015	5821	23
H(2)	4226	4661	7376	29
H(3)	5297	4298	8868	34
H(4)	7404	4275	8791	37
H(5)	8445	4607	7242	28
H(8)	9066	3540	6032	21
H(10)	10741	2549	5141	22
H(11)	11549	2339	3628	22
H(14)	8920	5412	1949	19
H(19A)	5400	8308	4969	21
H(19B)	6542	7149	5667	21
H(22)	7376	6704	1952	16
H(24)	9026	7414	1084	25
H(25)	10335	8634	717	30
H(26)	10199	9813	1649	28
H(27)	8744	9764	2957	25
H(28)	7431	8550	3325	21
H(30)	6548	7857	547	18
H(33)	3097	9635	−891	21
H(34)	2211	9704	640	22
H(36)	2427	9261	2468	21
H(39)	3548	6646	5139	23
H(40)	2238	6844	6474	26
H(41)	1117	8727	6437	28
H(42)	1352	10423	5070	35
H(43)	2694	10235	3742	30

Table S6. Torsion angles [°] for 7-methylene-6,8,14-triphenyl-7,14-dihydrobenzo[m]tetraphene-2,12-diyl bis(trifluoromethanesulfonate) (**8b**).

C(6)–C(1)–C(2)–C(3)	–0.1(2)
C(1)–C(2)–C(3)–C(4)	–0.5(2)
C(2)–C(3)–C(4)–C(5)	0.1(3)
C(3)–C(4)–C(5)–C(6)	0.9(3)
C(2)–C(1)–C(6)–C(5)	1.0(2)
C(2)–C(1)–C(6)–C(7)	176.31(13)
C(4)–C(5)–C(6)–C(1)	–1.4(2)
C(4)–C(5)–C(6)–C(7)	–176.70(14)
C(1)–C(6)–C(7)–C(8)	–122.16(15)
C(5)–C(6)–C(7)–C(8)	53.05(19)
C(1)–C(6)–C(7)–C(17)	53.41(19)
C(5)–C(6)–C(7)–C(17)	–131.38(15)
C(17)–C(7)–C(8)–C(9)	–6.9(2)
C(6)–C(7)–C(8)–C(9)	168.91(13)
C(7)–C(8)–C(9)–C(10)	–174.99(14)
C(7)–C(8)–C(9)–C(15)	0.1(2)
C(8)–C(9)–C(10)–C(11)	171.53(14)
C(15)–C(9)–C(10)–C(11)	–3.5(2)
C(9)–C(10)–C(11)–C(12)	–0.3(2)
C(10)–C(11)–C(12)–C(14)	4.0(2)
C(10)–C(11)–C(12)–O(1)	178.96(12)
C(11)–C(12)–C(14)–C(15)	–3.6(2)
O(1)–C(12)–C(14)–C(15)	–178.75(12)
C(8)–C(9)–C(15)–C(14)	–171.34(12)
C(10)–C(9)–C(15)–C(14)	3.8(2)
C(8)–C(9)–C(15)–C(16)	6.45(19)
C(10)–C(9)–C(15)–C(16)	–178.41(13)
C(12)–C(14)–C(15)–C(9)	–0.4(2)
C(12)–C(14)–C(15)–C(16)	–178.09(13)
C(9)–C(15)–C(16)–C(17)	–6.2(2)
C(14)–C(15)–C(16)–C(17)	171.46(13)
C(9)–C(15)–C(16)–C(22)	170.54(12)
C(14)–C(15)–C(16)–C(22)	–11.8(2)
C(15)–C(16)–C(17)–C(7)	–0.6(2)
C(22)–C(16)–C(17)–C(7)	–177.25(12)
C(15)–C(16)–C(17)–C(18)	176.35(12)
C(22)–C(16)–C(17)–C(18)	–0.35(18)
C(8)–C(7)–C(17)–C(16)	7.1(2)
C(6)–C(7)–C(17)–C(16)	–168.43(12)
C(8)–C(7)–C(17)–C(18)	–169.66(13)
C(6)–C(7)–C(17)–C(18)	14.8(2)
C(16)–C(17)–C(18)–C(19)	–140.01(14)
C(7)–C(17)–C(18)–C(19)	36.8(2)
C(16)–C(17)–C(18)–C(20)	33.42(17)
C(7)–C(17)–C(18)–C(20)	–149.77(13)
C(19)–C(18)–C(20)–C(21)	139.96(14)
C(17)–C(18)–C(20)–C(21)	–33.40(17)
C(19)–C(18)–C(20)–C(37)	–39.4(2)
C(17)–C(18)–C(20)–C(37)	147.23(13)
C(37)–C(20)–C(21)–C(29)	–1.4(2)
C(18)–C(20)–C(21)–C(29)	179.26(12)
C(37)–C(20)–C(21)–C(22)	179.64(12)
C(18)–C(20)–C(21)–C(22)	0.26(19)
C(20)–C(21)–C(22)–C(16)	31.23(17)
C(29)–C(21)–C(22)–C(16)	–147.80(12)
C(20)–C(21)–C(22)–C(23)	–93.78(15)
C(29)–C(21)–C(22)–C(23)	87.20(15)
C(17)–C(16)–C(22)–C(21)	–31.22(17)
C(15)–C(16)–C(22)–C(21)	152.01(12)
C(17)–C(16)–C(22)–C(23)	95.51(14)
C(15)–C(16)–C(22)–C(23)	–81.26(15)
C(21)–C(22)–C(23)–C(28)	54.12(17)

C(16)–C(22)–C(23)–C(28)	–72.17(15)
C(21)–C(22)–C(23)–C(24)	–127.05(13)
C(16)–C(22)–C(23)–C(24)	106.66(14)
C(28)–C(23)–C(24)–C(25)	0.2(2)
C(22)–C(23)–C(24)–C(25)	–178.65(14)
C(23)–C(24)–C(25)–C(26)	–0.1(2)
C(24)–C(25)–C(26)–C(27)	0.0(2)
C(25)–C(26)–C(27)–C(28)	–0.1(2)
C(26)–C(27)–C(28)–C(23)	0.2(2)
C(24)–C(23)–C(28)–C(27)	–0.3(2)
C(22)–C(23)–C(28)–C(27)	178.59(13)
C(20)–C(21)–C(29)–C(35)	–0.4(2)
C(22)–C(21)–C(29)–C(35)	178.62(12)
C(20)–C(21)–C(29)–C(30)	–179.84(12)
C(22)–C(21)–C(29)–C(30)	–0.8(2)
C(35)–C(29)–C(30)–C(31)	–1.76(19)
C(21)–C(29)–C(30)–C(31)	177.70(13)
C(29)–C(30)–C(31)–C(33)	0.9(2)
C(29)–C(30)–C(31)–O(4)	175.06(11)
C(30)–C(31)–C(33)–C(34)	0.2(2)
O(4)–C(31)–C(33)–C(34)	–174.04(12)
C(31)–C(33)–C(34)–C(35)	–0.4(2)
C(33)–C(34)–C(35)–C(36)	179.59(14)
C(33)–C(34)–C(35)–C(29)	–0.5(2)
C(30)–C(29)–C(35)–C(36)	–178.51(12)
C(21)–C(29)–C(35)–C(36)	2.0(2)
C(30)–C(29)–C(35)–C(34)	1.6(2)
C(21)–C(29)–C(35)–C(34)	–177.92(13)
C(34)–C(35)–C(36)–C(37)	177.99(14)
C(29)–C(35)–C(36)–C(37)	–1.9(2)
C(35)–C(36)–C(37)–C(20)	0.2(2)
C(35)–C(36)–C(37)–C(38)	–177.71(13)
C(21)–C(20)–C(37)–C(36)	1.5(2)
C(18)–C(20)–C(37)–C(36)	–179.17(13)
C(21)–C(20)–C(37)–C(38)	179.14(13)
C(18)–C(20)–C(37)–C(38)	–1.5(2)
C(36)–C(37)–C(38)–C(39)	115.46(15)
C(20)–C(37)–C(38)–C(39)	–62.3(2)
C(36)–C(37)–C(38)–C(43)	–57.67(19)
C(20)–C(37)–C(38)–C(43)	124.59(16)
C(43)–C(38)–C(39)–C(40)	–0.2(2)
C(37)–C(38)–C(39)–C(40)	–173.33(13)
C(38)–C(39)–C(40)–C(41)	0.8(2)
C(39)–C(40)–C(41)–C(42)	–0.8(2)
C(40)–C(41)–C(42)–C(43)	0.2(3)
C(41)–C(42)–C(43)–C(38)	0.5(3)
C(39)–C(38)–C(43)–C(42)	–0.5(2)
C(37)–C(38)–C(43)–C(42)	172.83(15)
C(14)–C(12)–O(1)–S(1)	–114.47(12)
C(11)–C(12)–O(1)–S(1)	70.15(16)
C(30)–C(31)–O(4)–S(2)	82.45(14)
C(33)–C(31)–O(4)–S(2)	–102.99(13)
C(12)–O(1)–S(1)–O(3)	–168.46(10)
C(12)–O(1)–S(1)–O(2)	–31.45(12)
C(12)–O(1)–S(1)–C(13)	81.37(11)
F(2)–C(13)–S(1)–O(3)	–53.89(13)
F(3)–C(13)–S(1)–O(3)	–175.09(10)
F(1)–C(13)–S(1)–O(3)	65.79(12)
F(2)–C(13)–S(1)–O(2)	174.66(11)
F(3)–C(13)–S(1)–O(2)	53.46(12)
F(1)–C(13)–S(1)–O(2)	–65.66(11)
F(2)–C(13)–S(1)–O(1)	56.71(12)
F(3)–C(13)–S(1)–O(1)	–64.48(11)
F(1)–C(13)–S(1)–O(1)	176.40(10)

C(31)–O(4)–S(2)–O(5)	–19.85(12)
C(31)–O(4)–S(2)–O(6)	–156.69(10)
C(31)–O(4)–S(2)–C(32)	94.70(11)
F(4)–C(32)–S(2)–O(5)	–67.54(13)
F(5)–C(32)–S(2)–O(5)	172.90(10)
F(6)–C(32)–S(2)–O(5)	52.63(13)
F(4)–C(32)–S(2)–O(6)	64.80(13)
F(5)–C(32)–S(2)–O(6)	–54.76(12)
F(6)–C(32)–S(2)–O(6)	–175.03(11)
F(4)–C(32)–S(2)–O(4)	175.06(11)
F(5)–C(32)–S(2)–O(4)	55.50(12)
F(6)–C(32)–S(2)–O(4)	–64.77(12)

Symmetry transformations used to generate equivalent atoms:

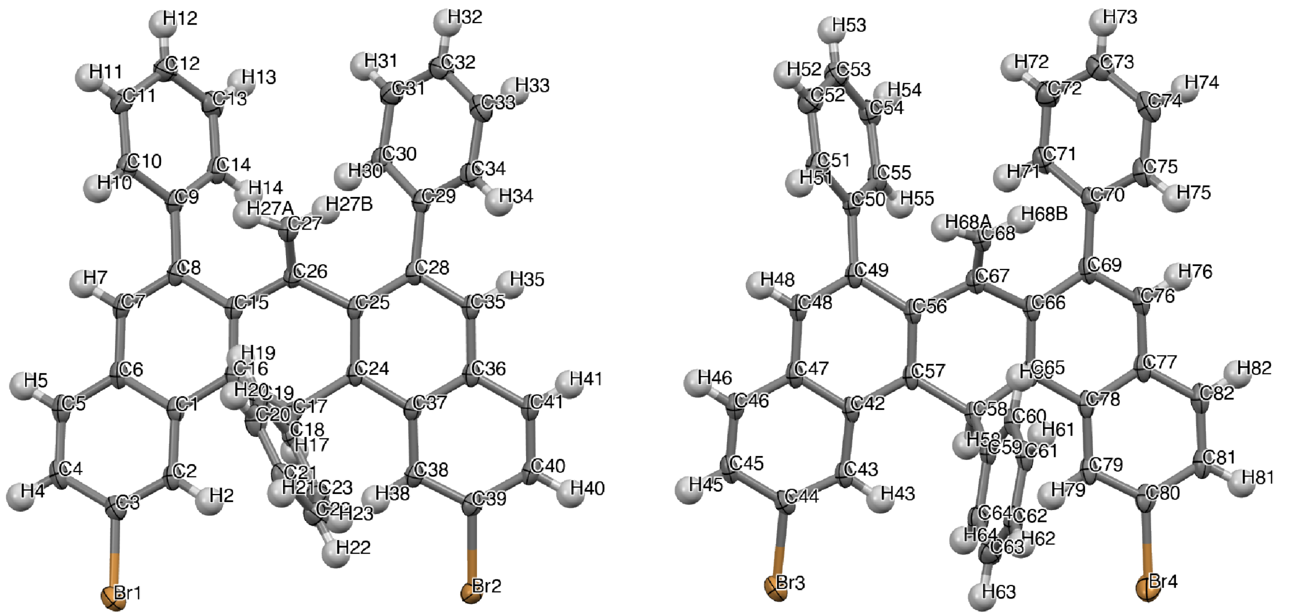

Figure S24. Single crystal X-ray structure diagram of 2,12-dibromo-7-methylene-6,8,14-triphenyl-7,14-dihydrobenzo[m]tetraphene (**1b**). ORTEP thermal ellipsoids are at the 50% probability level. Color coding: C (gray), H (white), Br (orange).

Table S7. Crystal data and structure refinement for 2,12-dibromo-7-methylene-6,8,14-triphenyl-7,14-dihydrobenzo[m]tetraphene (**1b**).

Identification code	CCDC 2130874	
Empirical formula	$C_{41}H_{26}Br_2$	
Formula weight	678.44	
Temperature	100(2) K	
Wavelength	1.54184 Å	
Crystal system	Triclinic	
Space group	P –1	
Unit cell dimensions	a = 14.2199(2) Å	α = 101.1430(10)°.
	b = 15.4193(2) Å	β = 104.0640(10)°.
	c = 16.50750(10) Å	γ = 115.6900(10)°.
Volume	2973.59(7) Å^3	
Z	4	
Density (calculated)	1.515 Mg/m^3	
Absorption coefficient	3.673 mm^{-1}	
F(000)	1368	
Crystal size	0.180 × 0.100 × 0.050 mm^3	
Theta range for data collection	2.936 to 74.487°	
Index ranges	$-17 \le h \le 17$, $-19 \le k \le 19$, $-20 \le l \le 20$	
Reflections collected	92521	
Independent reflections	12009 [R(int) = 0.0465]	
Completeness to theta = 74.000°	98.9 %	
Absorption correction	Semi–empirical from equivalents	
Max. and min. transmission	1.00000 and 0.65357	
Refinement method	Full–matrix least–squares on F^2	
Data / restraints / parameters	12009 / 0 / 775	
Goodness–of–fit on F^2	1.084	
Final R indices [I>2sigma(I)]	R1 = 0.0352, wR2 = 0.0934	
R indices (all data)	R1 = 0.0374, wR2 = 0.0952	
Extinction coefficient	n/a	
Largest diff. peak and hole	0.763 and –1.119 e Å^{-3}	

Table S8. Atomic coordinates (× 10^4) and equivalent isotropic displacement parameters (Å^2 × 10^3) for 2,12-dibromo-7-methylene-6,8,14-triphenyl-7,14-dihydrobenzo[m]tetraphene (**1b**). U(eq) is defined as one third of the trace of the orthogonalized U^{ij} tensor.

	x	y	z	U(eq)
C(1)	1369(2)	5060(2)	3800(1)	20(1)
C(2)	2167(2)	4742(2)	3847(1)	22(1)
C(3)	1840(2)	3731(2)	3686(2)	26(1)
C(4)	709(2)	2969(2)	3455(2)	28(1)
C(5)	−68(2)	3268(2)	3418(2)	27(1)
C(6)	235(2)	4314(2)	3603(1)	22(1)
C(7)	−557(2)	4636(2)	3586(1)	22(1)
C(8)	−274(2)	5645(2)	3762(1)	21(1)
C(9)	−1196(2)	5881(2)	3665(1)	21(1)
C(10)	−1928(2)	5528(2)	4107(2)	26(1)
C(11)	−2827(2)	5695(2)	3987(2)	29(1)
C(12)	−2997(2)	6223(2)	3435(2)	30(1)
C(13)	−2285(2)	6559(2)	2975(2)	28(1)
C(14)	−1395(2)	6385(2)	3085(2)	24(1)
C(15)	880(2)	6408(2)	3990(1)	19(1)
C(16)	1658(2)	6108(2)	3966(1)	19(1)
C(17)	2872(2)	6901(2)	4198(1)	19(1)
C(18)	3577(2)	7148(2)	5165(1)	21(1)
C(19)	3119(2)	7132(2)	5822(2)	26(1)
C(20)	3758(2)	7347(2)	6697(2)	31(1)
C(21)	4870(2)	7597(2)	6932(2)	32(1)
C(22)	5345(2)	7635(2)	6289(2)	30(1)
C(23)	4699(2)	7400(2)	5410(2)	25(1)
C(24)	3004(2)	7869(2)	4011(1)	19(1)
C(25)	2267(2)	8200(2)	4114(1)	19(1)
C(26)	1317(2)	7534(2)	4350(1)	20(1)
C(27)	974(2)	7913(2)	4938(2)	23(1)
C(28)	2455(2)	9172(2)	4032(1)	20(1)
C(29)	1696(2)	9597(2)	4087(1)	21(1)
C(30)	537(2)	9018(2)	3615(2)	26(1)
C(31)	−138(2)	9448(2)	3636(2)	30(1)
C(32)	331(2)	10472(2)	4137(2)	31(1)
C(33)	1476(2)	11062(2)	4606(2)	31(1)
C(34)	2151(2)	10627(2)	4581(2)	26(1)
C(35)	3409(2)	9768(2)	3887(1)	21(1)
C(36)	4162(2)	9441(2)	3763(1)	21(1)
C(37)	3944(2)	8458(2)	3788(1)	19(1)
C(38)	4659(2)	8112(2)	3573(1)	21(1)
C(39)	5554(2)	8741(2)	3391(1)	22(1)
C(40)	5824(2)	9744(2)	3428(1)	23(1)
C(41)	5124(2)	10073(2)	3599(1)	23(1)
C(42)	4816(2)	9545(2)	1164(1)	21(1)
C(43)	5782(2)	9449(2)	1389(1)	22(1)
C(44)	6797(2)	10234(2)	1474(2)	24(1)
C(45)	6922(2)	11132(2)	1315(2)	26(1)
C(46)	6003(2)	11241(2)	1106(2)	24(1)
C(47)	4941(2)	10472(2)	1042(1)	22(1)
C(48)	4037(2)	10655(2)	930(1)	23(1)
C(49)	3024(2)	9959(2)	925(1)	22(1)
C(50)	2235(2)	10312(2)	1052(2)	22(1)
C(51)	1909(2)	10821(2)	528(2)	28(1)
C(52)	1263(2)	11235(2)	722(2)	33(1)
C(53)	934(2)	11155(2)	1445(2)	31(1)
C(54)	1262(2)	10662(2)	1974(2)	29(1)
C(55)	1906(2)	10243(2)	1780(2)	25(1)
C(56)	2843(2)	8957(2)	910(1)	20(1)
C(57)	3717(2)	8762(2)	1055(1)	20(1)
C(58)	3517(2)	7709(2)	1060(1)	20(1)

C(59)	3498(2)	7098(2)	196(1)	21(1)
C(60)	2557(2)	6653(2)	–587(1)	22(1)
C(61)	2540(2)	6115(2)	–1377(2)	27(1)
C(62)	3454(2)	6020(2)	–1407(2)	31(1)
C(63)	4390(2)	6446(2)	–634(2)	32(1)
C(64)	4402(2)	6972(2)	164(2)	27(1)
C(65)	2450(2)	7076(2)	1219(1)	21(1)
C(66)	1569(2)	7263(2)	1030(1)	21(1)
C(67)	1689(2)	8082(2)	639(1)	21(1)
C(68)	842(2)	7986(2)	–17(2)	24(1)
C(69)	538(2)	6621(2)	1137(1)	23(1)
C(70)	–408(2)	6819(2)	1027(2)	24(1)
C(71)	–238(2)	7788(2)	1430(2)	29(1)
C(72)	–1137(2)	7954(2)	1335(2)	34(1)
C(73)	–2227(2)	7153(2)	839(2)	34(1)
C(74)	–2408(2)	6188(2)	452(2)	33(1)
C(75)	–1514(2)	6024(2)	536(2)	28(1)
C(76)	431(2)	5778(2)	1361(2)	25(1)
C(77)	1285(2)	5526(2)	1501(1)	24(1)
C(78)	2334(2)	6199(2)	1464(1)	22(1)
C(79)	3186(2)	5943(2)	1628(1)	25(1)
C(80)	2980(2)	5046(2)	1780(2)	27(1)
C(81)	1932(2)	4353(2)	1788(2)	28(1)
C(82)	1117(2)	4609(2)	1665(2)	27(1)
Br(1)	2938(1)	3339(1)	3754(1)	34(1)
Br(2)	6442(1)	8220(1)	3037(1)	27(1)
Br(3)	8106(1)	10126(1)	1856(1)	31(1)
Br(4)	4130(1)	4707(1)	1987(1)	38(1)

Table S9. Bond lengths [Å] and angles [°] for 2,12-dibromo-7-methylene-6,8,14-triphenyl-7,14-dihydrobenzo[m]tetraphene (**1b**).

C(1)–C(2)	1.409(3)
C(1)–C(6)	1.422(3)
C(1)–C(16)	1.433(3)
C(2)–C(3)	1.367(3)
C(2)–H(2)	0.9500
C(3)–C(4)	1.415(3)
C(3)–Br(1)	1.891(2)
C(4)–C(5)	1.362(4)
C(4)–H(4)	0.9500
C(5)–C(6)	1.422(3)
C(5)–H(5)	0.9500
C(6)–C(7)	1.412(3)
C(7)–C(8)	1.375(3)
C(7)–H(7)	0.9500
C(8)–C(15)	1.441(3)
C(8)–C(9)	1.486(3)
C(9)–C(10)	1.394(3)
C(9)–C(14)	1.397(3)
C(10)–C(11)	1.386(3)
C(10)–H(10)	0.9500
C(11)–C(12)	1.380(4)
C(11)–H(11)	0.9500
C(12)–C(13)	1.394(4)
C(12)–H(12)	0.9500
C(13)–C(14)	1.381(3)
C(13)–H(13)	0.9500
C(14)–H(14)	0.9500
C(15)–C(16)	1.377(3)
C(15)–C(26)	1.496(3)
C(16)–C(17)	1.512(3)
C(17)–C(24)	1.524(3)
C(17)–C(18)	1.534(3)
C(17)–H(17)	1.0000
C(18)–C(19)	1.394(3)
C(18)–C(23)	1.395(3)
C(19)–C(20)	1.392(3)
C(19)–H(19)	0.9500
C(20)–C(21)	1.384(4)
C(20)–H(20)	0.9500
C(21)–C(22)	1.388(4)
C(21)–H(21)	0.9500
C(22)–C(23)	1.393(3)
C(22)–H(22)	0.9500
C(23)–H(23)	0.9500
C(24)–C(25)	1.381(3)
C(24)–C(37)	1.436(3)
C(25)–C(28)	1.443(3)
C(25)–C(26)	1.497(3)
C(26)–C(27)	1.333(3)
C(27)–H(27A)	0.9500
C(27)–H(27B)	0.9500
C(28)–C(35)	1.380(3)
C(28)–C(29)	1.497(3)
C(29)–C(30)	1.397(3)
C(29)–C(34)	1.400(3)
C(30)–C(31)	1.386(3)
C(30)–H(30)	0.9500
C(31)–C(32)	1.392(4)
C(31)–H(31)	0.9500
C(32)–C(33)	1.382(4)
C(32)–H(32)	0.9500

C(33)–C(34)	1.391(3)
C(33)–H(33)	0.9500
C(34)–H(34)	0.9500
C(35)–C(36)	1.407(3)
C(35)–H(35)	0.9500
C(36)–C(37)	1.421(3)
C(36)–C(41)	1.422(3)
C(37)–C(38)	1.421(3)
C(38)–C(39)	1.371(3)
C(38)–H(38)	0.9500
C(39)–C(40)	1.407(3)
C(39)–Br(2)	1.907(2)
C(40)–C(41)	1.359(3)
C(40)–H(40)	0.9500
C(41)–H(41)	0.9500
C(42)–C(43)	1.414(3)
C(42)–C(47)	1.422(3)
C(42)–C(57)	1.441(3)
C(43)–C(44)	1.374(3)
C(43)–H(43)	0.9500
C(44)–C(45)	1.403(3)
C(44)–Br(3)	1.906(2)
C(45)–C(46)	1.360(3)
C(45)–H(45)	0.9500
C(46)–C(47)	1.421(3)
C(46)–H(46)	0.9500
C(47)–C(48)	1.409(3)
C(48)–C(49)	1.371(3)
C(48)–H(48)	0.9500
C(49)–C(56)	1.445(3)
C(49)–C(50)	1.483(3)
C(50)–C(55)	1.399(3)
C(50)–C(51)	1.399(3)
C(51)–C(52)	1.389(4)
C(51)–H(51)	0.9500
C(52)–C(53)	1.391(4)
C(52)–H(52)	0.9500
C(53)–C(54)	1.387(4)
C(53)–H(53)	0.9500
C(54)–C(55)	1.392(3)
C(54)–H(54)	0.9500
C(55)–H(55)	0.9500
C(56)–C(57)	1.378(3)
C(56)–C(67)	1.485(3)
C(57)–C(58)	1.524(3)
C(58)–C(65)	1.527(3)
C(58)–C(59)	1.537(3)
C(58)–H(58)	1.0000
C(59)–C(64)	1.392(3)
C(59)–C(60)	1.396(3)
C(60)–C(61)	1.392(3)
C(60)–H(60)	0.9500
C(61)–C(62)	1.381(4)
C(61)–H(61)	0.9500
C(62)–C(63)	1.387(4)
C(62)–H(62)	0.9500
C(63)–C(64)	1.399(3)
C(63)–H(63)	0.9500
C(64)–H(64)	0.9500
C(65)–C(66)	1.381(3)
C(65)–C(78)	1.440(3)
C(66)–C(69)	1.449(3)
C(66)–C(67)	1.490(3)
C(67)–C(68)	1.340(3)

C(68)–H(68A)	0.9500
C(68)–H(68B)	0.9500
C(69)–C(76)	1.375(3)
C(69)–C(70)	1.481(3)
C(70)–C(75)	1.399(3)
C(70)–C(71)	1.400(3)
C(71)–C(72)	1.387(4)
C(71)–H(71)	0.9500
C(72)–C(73)	1.390(4)
C(72)–H(72)	0.9500
C(73)–C(74)	1.382(4)
C(73)–H(73)	0.9500
C(74)–C(75)	1.381(4)
C(74)–H(74)	0.9500
C(75)–H(75)	0.9500
C(76)–C(77)	1.410(4)
C(76)–H(76)	0.9500
C(77)–C(82)	1.420(3)
C(77)–C(78)	1.426(3)
C(78)–C(79)	1.411(3)
C(79)–C(80)	1.372(3)
C(79)–H(79)	0.9500
C(80)–C(81)	1.413(3)
C(80)–Br(4)	1.899(3)
C(81)–C(82)	1.361(4)
C(81)–H(81)	0.9500
C(82)–H(82)	0.9500

C(2)–C(1)–C(6)	118.6(2)
C(2)–C(1)–C(16)	122.8(2)
C(6)–C(1)–C(16)	118.6(2)
C(3)–C(2)–C(1)	120.0(2)
C(3)–C(2)–H(2)	120.0
C(1)–C(2)–H(2)	120.0
C(2)–C(3)–C(4)	122.3(2)
C(2)–C(3)–Br(1)	118.62(18)
C(4)–C(3)–Br(1)	119.02(17)
C(5)–C(4)–C(3)	118.2(2)
C(5)–C(4)–H(4)	120.9
C(3)–C(4)–H(4)	120.9
C(4)–C(5)–C(6)	121.5(2)
C(4)–C(5)–H(5)	119.2
C(6)–C(5)–H(5)	119.2
C(7)–C(6)–C(1)	118.7(2)
C(7)–C(6)–C(5)	122.1(2)
C(1)–C(6)–C(5)	119.2(2)
C(8)–C(7)–C(6)	122.6(2)
C(8)–C(7)–H(7)	118.7
C(6)–C(7)–H(7)	118.7
C(7)–C(8)–C(15)	118.9(2)
C(7)–C(8)–C(9)	117.63(19)
C(15)–C(8)–C(9)	123.43(19)
C(10)–C(9)–C(14)	119.0(2)
C(10)–C(9)–C(8)	119.8(2)
C(14)–C(9)–C(8)	121.0(2)
C(11)–C(10)–C(9)	120.6(2)
C(11)–C(10)–H(10)	119.7
C(9)–C(10)–H(10)	119.7
C(12)–C(11)–C(10)	120.1(2)
C(12)–C(11)–H(11)	120.0
C(10)–C(11)–H(11)	120.0
C(11)–C(12)–C(13)	119.8(2)
C(11)–C(12)–H(12)	120.1
C(13)–C(12)–H(12)	120.1

C(14)–C(13)–C(12)	120.2(2)
C(14)–C(13)–H(13)	119.9
C(12)–C(13)–H(13)	119.9
C(13)–C(14)–C(9)	120.3(2)
C(13)–C(14)–H(14)	119.9
C(9)–C(14)–H(14)	119.9
C(16)–C(15)–C(8)	119.57(19)
C(16)–C(15)–C(26)	116.97(18)
C(8)–C(15)–C(26)	123.12(19)
C(15)–C(16)–C(1)	121.41(19)
C(15)–C(16)–C(17)	120.13(19)
C(1)–C(16)–C(17)	118.24(19)
C(16)–C(17)–C(24)	112.72(17)
C(16)–C(17)–C(18)	110.79(17)
C(24)–C(17)–C(18)	111.35(17)
C(16)–C(17)–H(17)	107.2
C(24)–C(17)–H(17)	107.2
C(18)–C(17)–H(17)	107.2
C(19)–C(18)–C(23)	118.2(2)
C(19)–C(18)–C(17)	121.0(2)
C(23)–C(18)–C(17)	120.8(2)
C(20)–C(19)–C(18)	120.9(2)
C(20)–C(19)–H(19)	119.6
C(18)–C(19)–H(19)	119.6
C(21)–C(20)–C(19)	120.3(2)
C(21)–C(20)–H(20)	119.8
C(19)–C(20)–H(20)	119.8
C(20)–C(21)–C(22)	119.5(2)
C(20)–C(21)–H(21)	120.3
C(22)–C(21)–H(21)	120.3
C(21)–C(22)–C(23)	120.1(2)
C(21)–C(22)–H(22)	119.9
C(23)–C(22)–H(22)	119.9
C(22)–C(23)–C(18)	120.9(2)
C(22)–C(23)–H(23)	119.5
C(18)–C(23)–H(23)	119.5
C(25)–C(24)–C(37)	121.14(19)
C(25)–C(24)–C(17)	119.73(19)
C(37)–C(24)–C(17)	119.00(19)
C(24)–C(25)–C(28)	120.20(19)
C(24)–C(25)–C(26)	117.06(19)
C(28)–C(25)–C(26)	122.69(19)
C(27)–C(26)–C(15)	121.1(2)
C(27)–C(26)–C(25)	122.5(2)
C(15)–C(26)–C(25)	115.75(18)
C(26)–C(27)–H(27A)	120.0
C(26)–C(27)–H(27B)	120.0
H(27A)–C(27)–H(27B)	120.0
C(35)–C(28)–C(25)	118.1(2)
C(35)–C(28)–C(29)	116.35(19)
C(25)–C(28)–C(29)	125.53(19)
C(30)–C(29)–C(34)	117.6(2)
C(30)–C(29)–C(28)	122.3(2)
C(34)–C(29)–C(28)	119.9(2)
C(31)–C(30)–C(29)	121.2(2)
C(31)–C(30)–H(30)	119.4
C(29)–C(30)–H(30)	119.4
C(30)–C(31)–C(32)	120.3(2)
C(30)–C(31)–H(31)	119.9
C(32)–C(31)–H(31)	119.9
C(33)–C(32)–C(31)	119.6(2)
C(33)–C(32)–H(32)	120.2
C(31)–C(32)–H(32)	120.2
C(32)–C(33)–C(34)	119.9(2)

C(32)–C(33)–H(33)	120.0
C(34)–C(33)–H(33)	120.0
C(33)–C(34)–C(29)	121.4(2)
C(33)–C(34)–H(34)	119.3
C(29)–C(34)–H(34)	119.3
C(28)–C(35)–C(36)	122.8(2)
C(28)–C(35)–H(35)	118.6
C(36)–C(35)–H(35)	118.6
C(35)–C(36)–C(37)	119.28(19)
C(35)–C(36)–C(41)	120.9(2)
C(37)–C(36)–C(41)	119.8(2)
C(38)–C(37)–C(36)	117.70(19)
C(38)–C(37)–C(24)	124.1(2)
C(36)–C(37)–C(24)	118.2(2)
C(39)–C(38)–C(37)	119.9(2)
C(39)–C(38)–H(38)	120.1
C(37)–C(38)–H(38)	120.1
C(38)–C(39)–C(40)	122.8(2)
C(38)–C(39)–Br(2)	118.58(17)
C(40)–C(39)–Br(2)	118.64(17)
C(41)–C(40)–C(39)	118.1(2)
C(41)–C(40)–H(40)	120.9
C(39)–C(40)–H(40)	120.9
C(40)–C(41)–C(36)	121.5(2)
C(40)–C(41)–H(41)	119.2
C(36)–C(41)–H(41)	119.2
C(43)–C(42)–C(47)	117.85(19)
C(43)–C(42)–C(57)	123.8(2)
C(47)–C(42)–C(57)	118.4(2)
C(44)–C(43)–C(42)	119.9(2)
C(44)–C(43)–H(43)	120.1
C(42)–C(43)–H(43)	120.1
C(43)–C(44)–C(45)	122.9(2)
C(43)–C(44)–Br(3)	119.09(18)
C(45)–C(44)–Br(3)	118.01(17)
C(46)–C(45)–C(44)	117.9(2)
C(46)–C(45)–H(45)	121.0
C(44)–C(45)–H(45)	121.0
C(45)–C(46)–C(47)	121.7(2)
C(45)–C(46)–H(46)	119.2
C(47)–C(46)–H(46)	119.2
C(48)–C(47)–C(46)	120.4(2)
C(48)–C(47)–C(42)	119.7(2)
C(46)–C(47)–C(42)	119.7(2)
C(49)–C(48)–C(47)	121.9(2)
C(49)–C(48)–H(48)	119.0
C(47)–C(48)–H(48)	119.0
C(48)–C(49)–C(56)	118.0(2)
C(48)–C(49)–C(50)	118.2(2)
C(56)–C(49)–C(50)	123.34(19)
C(55)–C(50)–C(51)	118.4(2)
C(55)–C(50)–C(49)	119.0(2)
C(51)–C(50)–C(49)	122.2(2)
C(52)–C(51)–C(50)	120.7(2)
C(52)–C(51)–H(51)	119.6
C(50)–C(51)–H(51)	119.6
C(51)–C(52)–C(53)	120.3(2)
C(51)–C(52)–H(52)	119.9
C(53)–C(52)–H(52)	119.9
C(54)–C(53)–C(52)	119.5(2)
C(54)–C(53)–H(53)	120.2
C(52)–C(53)–H(53)	120.2
C(53)–C(54)–C(55)	120.3(2)
C(53)–C(54)–H(54)	119.8

C(55)–C(54)–H(54)	119.8
C(54)–C(55)–C(50)	120.7(2)
C(54)–C(55)–H(55)	119.7
C(50)–C(55)–H(55)	119.7
C(57)–C(56)–C(49)	121.12(19)
C(57)–C(56)–C(67)	118.02(19)
C(49)–C(56)–C(67)	120.5(2)
C(56)–C(57)–C(42)	119.7(2)
C(56)–C(57)–C(58)	120.42(19)
C(42)–C(57)–C(58)	119.8(2)
C(57)–C(58)–C(65)	113.73(18)
C(57)–C(58)–C(59)	111.17(17)
C(65)–C(58)–C(59)	109.64(17)
C(57)–C(58)–H(58)	107.3
C(65)–C(58)–H(58)	107.3
C(59)–C(58)–H(58)	107.3
C(64)–C(59)–C(60)	118.2(2)
C(64)–C(59)–C(58)	121.8(2)
C(60)–C(59)–C(58)	120.0(2)
C(61)–C(60)–C(59)	120.5(2)
C(61)–C(60)–H(60)	119.7
C(59)–C(60)–H(60)	119.7
C(62)–C(61)–C(60)	120.9(2)
C(62)–C(61)–H(61)	119.6
C(60)–C(61)–H(61)	119.6
C(61)–C(62)–C(63)	119.4(2)
C(61)–C(62)–H(62)	120.3
C(63)–C(62)–H(62)	120.3
C(62)–C(63)–C(64)	119.8(2)
C(62)–C(63)–H(63)	120.1
C(64)–C(63)–H(63)	120.1
C(59)–C(64)–C(63)	121.2(2)
C(59)–C(64)–H(64)	119.4
C(63)–C(64)–H(64)	119.4
C(66)–C(65)–C(78)	120.8(2)
C(66)–C(65)–C(58)	121.05(19)
C(78)–C(65)–C(58)	117.7(2)
C(65)–C(66)–C(69)	120.5(2)
C(65)–C(66)–C(67)	117.36(19)
C(69)–C(66)–C(67)	122.0(2)
C(68)–C(67)–C(56)	119.9(2)
C(68)–C(67)–C(66)	122.7(2)
C(56)–C(67)–C(66)	116.71(19)
C(67)–C(68)–H(68A)	120.0
C(67)–C(68)–H(68B)	120.0
H(68A)–C(68)–H(68B)	120.0
C(76)–C(69)–C(66)	118.1(2)
C(76)–C(69)–C(70)	117.8(2)
C(66)–C(69)–C(70)	124.0(2)
C(75)–C(70)–C(71)	117.7(2)
C(75)–C(70)–C(69)	120.5(2)
C(71)–C(70)–C(69)	121.7(2)
C(72)–C(71)–C(70)	121.0(2)
C(72)–C(71)–H(71)	119.5
C(70)–C(71)–H(71)	119.5
C(71)–C(72)–C(73)	120.2(2)
C(71)–C(72)–H(72)	119.9
C(73)–C(72)–H(72)	119.9
C(74)–C(73)–C(72)	119.3(3)
C(74)–C(73)–H(73)	120.3
C(72)–C(73)–H(73)	120.3
C(75)–C(74)–C(73)	120.5(2)
C(75)–C(74)–H(74)	119.7
C(73)–C(74)–H(74)	119.7

C(74)–C(75)–C(70)	121.2(2)
C(74)–C(75)–H(75)	119.4
C(70)–C(75)–H(75)	119.4
C(69)–C(76)–C(77)	122.8(2)
C(69)–C(76)–H(76)	118.6
C(77)–C(76)–H(76)	118.6
C(76)–C(77)–C(82)	121.4(2)
C(76)–C(77)–C(78)	119.3(2)
C(82)–C(77)–C(78)	119.3(2)
C(79)–C(78)–C(77)	118.3(2)
C(79)–C(78)–C(65)	123.5(2)
C(77)–C(78)–C(65)	118.2(2)
C(80)–C(79)–C(78)	120.1(2)
C(80)–C(79)–H(79)	120.0
C(78)–C(79)–H(79)	120.0
C(79)–C(80)–C(81)	122.3(2)
C(79)–C(80)–Br(4)	119.52(18)
C(81)–C(80)–Br(4)	118.16(18)
C(82)–C(81)–C(80)	118.2(2)
C(82)–C(81)–H(81)	120.9
C(80)–C(81)–H(81)	120.9
C(81)–C(82)–C(77)	121.7(2)
C(81)–C(82)–H(82)	119.1
C(77)–C(82)–H(82)	119.1

Symmetry transformations used to generate equivalent atoms:

Table S10. Anisotropic displacement parameters (Å^2 × 10^3) for 2,12-dibromo-7-methylene-6,8,14-triphenyl-7,14-dihydrobenzo[m]tetraphene (**1b**). The anisotropic displacement factor exponent takes the form: $-2p^2$ [$h^2a^{*2}U^{11}$ + ... + 2 h k a* b* U^{12}]

	U^{11}	U^{22}	U^{33}	U^{23}	U^{13}	U^{12}
C(1)	23(1)	16(1)	15(1)	4(1)	5(1)	5(1)
C(2)	26(1)	18(1)	19(1)	5(1)	8(1)	7(1)
C(3)	31(1)	21(1)	25(1)	8(1)	11(1)	13(1)
C(4)	35(1)	15(1)	26(1)	6(1)	10(1)	8(1)
C(5)	26(1)	17(1)	24(1)	6(1)	6(1)	3(1)
C(6)	23(1)	17(1)	17(1)	6(1)	4(1)	4(1)
C(7)	18(1)	18(1)	20(1)	6(1)	4(1)	2(1)
C(8)	19(1)	20(1)	17(1)	6(1)	5(1)	5(1)
C(9)	17(1)	16(1)	20(1)	3(1)	2(1)	2(1)
C(10)	22(1)	24(1)	24(1)	10(1)	6(1)	6(1)
C(11)	21(1)	28(1)	31(1)	8(1)	10(1)	7(1)
C(12)	22(1)	24(1)	33(1)	4(1)	4(1)	9(1)
C(13)	26(1)	22(1)	26(1)	6(1)	1(1)	8(1)
C(14)	22(1)	19(1)	22(1)	6(1)	5(1)	4(1)
C(15)	19(1)	16(1)	16(1)	5(1)	6(1)	5(1)
C(16)	20(1)	15(1)	14(1)	4(1)	5(1)	4(1)
C(17)	18(1)	16(1)	20(1)	6(1)	7(1)	7(1)
C(18)	22(1)	10(1)	22(1)	4(1)	4(1)	4(1)
C(19)	22(1)	19(1)	23(1)	5(1)	5(1)	3(1)
C(20)	34(1)	23(1)	21(1)	6(1)	7(1)	7(1)
C(21)	37(1)	23(1)	22(1)	4(1)	-1(1)	12(1)
C(22)	26(1)	23(1)	31(1)	4(1)	1(1)	12(1)
C(23)	24(1)	21(1)	28(1)	6(1)	6(1)	11(1)
C(24)	19(1)	14(1)	16(1)	4(1)	4(1)	5(1)
C(25)	17(1)	14(1)	14(1)	3(1)	3(1)	2(1)
C(26)	17(1)	16(1)	21(1)	6(1)	6(1)	5(1)
C(27)	23(1)	20(1)	24(1)	8(1)	9(1)	8(1)
C(28)	19(1)	16(1)	16(1)	4(1)	3(1)	6(1)
C(29)	22(1)	20(1)	19(1)	9(1)	8(1)	8(1)
C(30)	24(1)	20(1)	27(1)	9(1)	7(1)	7(1)
C(31)	23(1)	32(1)	34(1)	16(1)	10(1)	13(1)
C(32)	34(1)	34(1)	38(1)	18(1)	18(1)	22(1)
C(33)	38(1)	24(1)	31(1)	9(1)	13(1)	15(1)
C(34)	25(1)	22(1)	26(1)	7(1)	7(1)	10(1)
C(35)	22(1)	16(1)	20(1)	5(1)	6(1)	5(1)
C(36)	21(1)	17(1)	17(1)	5(1)	6(1)	5(1)
C(37)	17(1)	17(1)	16(1)	4(1)	3(1)	5(1)
C(38)	18(1)	20(1)	19(1)	6(1)	5(1)	6(1)
C(39)	20(1)	22(1)	19(1)	5(1)	5(1)	7(1)
C(40)	19(1)	21(1)	21(1)	7(1)	7(1)	3(1)
C(41)	23(1)	16(1)	22(1)	6(1)	7(1)	4(1)
C(42)	21(1)	16(1)	16(1)	2(1)	7(1)	2(1)
C(43)	22(1)	17(1)	18(1)	3(1)	7(1)	4(1)
C(44)	20(1)	23(1)	21(1)	4(1)	8(1)	6(1)
C(45)	23(1)	18(1)	24(1)	4(1)	11(1)	1(1)
C(46)	26(1)	16(1)	24(1)	6(1)	11(1)	4(1)
C(47)	24(1)	16(1)	19(1)	3(1)	8(1)	4(1)
C(48)	25(1)	15(1)	20(1)	5(1)	8(1)	4(1)
C(49)	23(1)	17(1)	17(1)	5(1)	6(1)	5(1)
C(50)	20(1)	15(1)	23(1)	4(1)	7(1)	3(1)
C(51)	31(1)	28(1)	26(1)	11(1)	12(1)	14(1)
C(52)	34(1)	33(1)	35(1)	15(1)	13(1)	18(1)
C(53)	26(1)	28(1)	35(1)	7(1)	11(1)	12(1)
C(54)	27(1)	23(1)	27(1)	5(1)	12(1)	6(1)
C(55)	24(1)	19(1)	23(1)	6(1)	6(1)	4(1)
C(56)	20(1)	15(1)	18(1)	4(1)	7(1)	3(1)
C(57)	21(1)	14(1)	14(1)	2(1)	4(1)	2(1)
C(58)	18(1)	15(1)	19(1)	4(1)	5(1)	3(1)

C(59)	21(1)	13(1)	21(1)	5(1)	7(1)	3(1)
C(60)	22(1)	15(1)	22(1)	7(1)	7(1)	2(1)
C(61)	27(1)	17(1)	21(1)	3(1)	6(1)	0(1)
C(62)	35(1)	17(1)	29(1)	3(1)	14(1)	5(1)
C(63)	31(1)	24(1)	36(1)	4(1)	15(1)	12(1)
C(64)	25(1)	21(1)	26(1)	4(1)	6(1)	8(1)
C(65)	21(1)	14(1)	16(1)	2(1)	5(1)	1(1)
C(66)	21(1)	15(1)	17(1)	2(1)	6(1)	2(1)
C(67)	21(1)	17(1)	19(1)	4(1)	8(1)	5(1)
C(68)	21(1)	19(1)	23(1)	6(1)	9(1)	4(1)
C(69)	22(1)	16(1)	20(1)	3(1)	8(1)	2(1)
C(70)	22(1)	21(1)	22(1)	7(1)	11(1)	4(1)
C(71)	26(1)	23(1)	30(1)	5(1)	13(1)	5(1)
C(72)	34(1)	29(1)	34(1)	7(1)	15(1)	13(1)
C(73)	30(1)	40(2)	33(1)	14(1)	13(1)	16(1)
C(74)	25(1)	35(1)	28(1)	10(1)	8(1)	6(1)
C(75)	26(1)	25(1)	24(1)	8(1)	9(1)	5(1)
C(76)	23(1)	18(1)	23(1)	6(1)	10(1)	1(1)
C(77)	26(1)	17(1)	17(1)	3(1)	7(1)	2(1)
C(78)	24(1)	15(1)	15(1)	3(1)	5(1)	2(1)
C(79)	22(1)	18(1)	19(1)	4(1)	2(1)	2(1)
C(80)	26(1)	21(1)	21(1)	6(1)	2(1)	7(1)
C(81)	32(1)	20(1)	21(1)	8(1)	6(1)	5(1)
C(82)	29(1)	19(1)	21(1)	7(1)	9(1)	2(1)
Br(1)	39(1)	24(1)	46(1)	12(1)	20(1)	19(1)
Br(2)	23(1)	29(1)	32(1)	13(1)	13(1)	13(1)
Br(3)	20(1)	28(1)	40(1)	11(1)	12(1)	7(1)
Br(4)	29(1)	30(1)	44(1)	16(1)	3(1)	12(1)

Table S11. Hydrogen coordinates (× 10^4) and isotropic displacement parameters (Å^2 × 10^3) for 2,12-dibromo-7-methylene-6,8,14-triphenyl-7,14-dihydrobenzo[m]tetraphene (**1b**).

	x	y	z	U(eq)
H(2)	2932	5231	3990	27
H(4)	498	2268	3329	33
H(5)	-831	2765	3265	32
H(7)	-1317	4136	3448	27
H(10)	-1811	5170	4492	31
H(11)	-3326	5446	4286	35
H(12)	-3599	6357	3368	35
H(13)	-2411	6909	2584	34
H(14)	-917	6610	2765	29
H(17)	3168	6592	3811	22
H(19)	2360	6973	5671	31
H(20)	3429	7322	7135	37
H(21)	5305	7741	7529	38
H(22)	6113	7822	6448	36
H(23)	5026	7411	4971	31
H(27A)	443	7467	5134	28
H(27B)	1260	8630	5160	28
H(30)	205	8317	3273	31
H(31)	-924	9042	3308	35
H(32)	-133	10765	4156	37
H(33)	1803	11763	4946	37
H(34)	2937	11039	4905	31
H(35)	3563	10427	3870	26
H(38)	4518	7446	3555	25
H(40)	6478	10179	3337	28
H(41)	5282	10741	3608	28
H(43)	5730	8844	1483	26
H(45)	7628	11649	1352	31
H(46)	6072	11846	999	29
H(48)	4133	11280	855	28
H(51)	2132	10883	35	34
H(52)	1044	11576	359	40
H(53)	489	11436	1575	38
H(54)	1045	10611	2471	34
H(55)	2124	9905	2146	30
H(58)	4165	7818	1559	24
H(60)	1924	6716	-580	27
H(61)	1891	5811	-1903	32
H(62)	3441	5665	-1952	37
H(63)	5021	6381	-646	38
H(64)	5038	7248	694	32
H(68A)	980	8478	-308	29
H(68B)	103	7427	-190	29
H(71)	503	8340	1774	35
H(72)	-1006	8618	1611	40
H(73)	-2843	7268	766	41
H(74)	-3152	5634	125	40
H(75)	-1652	5359	255	34
H(76)	-247	5347	1425	30
H(79)	3904	6393	1634	30
H(81)	1799	3725	1876	34
H(82)	418	4163	1691	33

Table S12. Torsion angles [°] for 2,12-dibromo-7-methylene-6,8,14-triphenyl-7,14-dihydrobenzo[m]tetraphene (**1b**).

C(6)–C(1)–C(2)–C(3)	1.3(3)
C(16)–C(1)–C(2)–C(3)	179.6(2)
C(1)–C(2)–C(3)–C(4)	1.1(3)
C(1)–C(2)–C(3)–Br(1)	179.92(16)
C(2)–C(3)–C(4)–C(5)	–1.8(4)
Br(1)–C(3)–C(4)–C(5)	179.33(17)
C(3)–C(4)–C(5)–C(6)	0.1(3)
C(2)–C(1)–C(6)–C(7)	177.82(19)
C(16)–C(1)–C(6)–C(7)	–0.5(3)
C(2)–C(1)–C(6)–C(5)	–2.9(3)
C(16)–C(1)–C(6)–C(5)	178.69(19)
C(4)–C(5)–C(6)–C(7)	–178.5(2)
C(4)–C(5)–C(6)–C(1)	2.2(3)
C(1)–C(6)–C(7)–C(8)	–0.7(3)
C(5)–C(6)–C(7)–C(8)	–179.9(2)
C(6)–C(7)–C(8)–C(15)	–1.2(3)
C(6)–C(7)–C(8)–C(9)	176.0(2)
C(7)–C(8)–C(9)–C(10)	53.2(3)
C(15)–C(8)–C(9)–C(10)	–129.8(2)
C(7)–C(8)–C(9)–C(14)	–122.0(2)
C(15)–C(8)–C(9)–C(14)	55.1(3)
C(14)–C(9)–C(10)–C(11)	–1.4(3)
C(8)–C(9)–C(10)–C(11)	–176.6(2)
C(9)–C(10)–C(11)–C(12)	–0.6(4)
C(10)–C(11)–C(12)–C(13)	2.0(4)
C(11)–C(12)–C(13)–C(14)	–1.4(4)
C(12)–C(13)–C(14)–C(9)	–0.7(3)
C(10)–C(9)–C(14)–C(13)	2.1(3)
C(8)–C(9)–C(14)–C(13)	177.2(2)
C(7)–C(8)–C(15)–C(16)	4.5(3)
C(9)–C(8)–C(15)–C(16)	–172.6(2)
C(7)–C(8)–C(15)–C(26)	–168.6(2)
C(9)–C(8)–C(15)–C(26)	14.3(3)
C(8)–C(15)–C(16)–C(1)	–5.8(3)
C(26)–C(15)–C(16)–C(1)	167.69(18)
C(8)–C(15)–C(16)–C(17)	179.63(18)
C(26)–C(15)–C(16)–C(17)	–6.9(3)
C(2)–C(1)–C(16)–C(15)	–174.4(2)
C(6)–C(1)–C(16)–C(15)	3.8(3)
C(2)–C(1)–C(16)–C(17)	0.2(3)
C(6)–C(1)–C(16)–C(17)	178.52(18)
C(15)–C(16)–C(17)–C(24)	–27.4(3)
C(1)–C(16)–C(17)–C(24)	157.81(18)
C(15)–C(16)–C(17)–C(18)	98.1(2)
C(1)–C(16)–C(17)–C(18)	–76.7(2)
C(16)–C(17)–C(18)–C(19)	–33.4(3)
C(24)–C(17)–C(18)–C(19)	92.9(2)
C(16)–C(17)–C(18)–C(23)	146.9(2)
C(24)–C(17)–C(18)–C(23)	–86.8(2)
C(23)–C(18)–C(19)–C(20)	–1.0(3)
C(17)–C(18)–C(19)–C(20)	179.3(2)
C(18)–C(19)–C(20)–C(21)	1.1(4)
C(19)–C(20)–C(21)–C(22)	0.2(4)
C(20)–C(21)–C(22)–C(23)	–1.5(4)
C(21)–C(22)–C(23)–C(18)	1.6(4)
C(19)–C(18)–C(23)–C(22)	–0.4(3)
C(17)–C(18)–C(23)–C(22)	179.3(2)
C(16)–C(17)–C(24)–C(25)	33.4(3)
C(18)–C(17)–C(24)–C(25)	–91.9(2)
C(16)–C(17)–C(24)–C(37)	–150.89(18)
C(18)–C(17)–C(24)–C(37)	83.9(2)
C(37)–C(24)–C(25)–C(28)	–2.3(3)

C(17)–C(24)–C(25)–C(28)	173.32(18)
C(37)–C(24)–C(25)–C(26)	–179.77(18)
C(17)–C(24)–C(25)–C(26)	–4.1(3)
C(16)–C(15)–C(26)–C(27)	–133.8(2)
C(8)–C(15)–C(26)–C(27)	39.5(3)
C(16)–C(15)–C(26)–C(25)	37.7(3)
C(8)–C(15)–C(26)–C(25)	–149.03(19)
C(24)–C(25)–C(26)–C(27)	139.5(2)
C(28)–C(25)–C(26)–C(27)	–37.8(3)
C(24)–C(25)–C(26)–C(15)	–31.8(3)
C(28)–C(25)–C(26)–C(15)	150.80(19)
C(24)–C(25)–C(28)–C(35)	–2.7(3)
C(26)–C(25)–C(28)–C(35)	174.54(19)
C(24)–C(25)–C(28)–C(29)	177.0(2)
C(26)–C(25)–C(28)–C(29)	–5.7(3)
C(35)–C(28)–C(29)–C(30)	131.7(2)
C(25)–C(28)–C(29)–C(30)	–48.1(3)
C(35)–C(28)–C(29)–C(34)	–44.1(3)
C(25)–C(28)–C(29)–C(34)	136.1(2)
C(34)–C(29)–C(30)–C(31)	–0.2(3)
C(28)–C(29)–C(30)–C(31)	–176.1(2)
C(29)–C(30)–C(31)–C(32)	–0.3(4)
C(30)–C(31)–C(32)–C(33)	0.6(4)
C(31)–C(32)–C(33)–C(34)	–0.4(4)
C(32)–C(33)–C(34)–C(29)	–0.1(4)
C(30)–C(29)–C(34)–C(33)	0.4(3)
C(28)–C(29)–C(34)–C(33)	176.4(2)
C(25)–C(28)–C(35)–C(36)	4.0(3)
C(29)–C(28)–C(35)–C(36)	–175.80(19)
C(28)–C(35)–C(36)–C(37)	–0.1(3)
C(28)–C(35)–C(36)–C(41)	178.8(2)
C(35)–C(36)–C(37)–C(38)	173.88(19)
C(41)–C(36)–C(37)–C(38)	–5.0(3)
C(35)–C(36)–C(37)–C(24)	–5.0(3)
C(41)–C(36)–C(37)–C(24)	176.13(19)
C(25)–C(24)–C(37)–C(38)	–172.6(2)
C(17)–C(24)–C(37)–C(38)	11.7(3)
C(25)–C(24)–C(37)–C(36)	6.2(3)
C(17)–C(24)–C(37)–C(36)	–169.51(18)
C(36)–C(37)–C(38)–C(39)	2.9(3)
C(24)–C(37)–C(38)–C(39)	–178.3(2)
C(37)–C(38)–C(39)–C(40)	1.7(3)
C(37)–C(38)–C(39)–Br(2)	–176.38(15)
C(38)–C(39)–C(40)–C(41)	–4.2(3)
Br(2)–C(39)–C(40)–C(41)	173.89(16)
C(39)–C(40)–C(41)–C(36)	1.9(3)
C(35)–C(36)–C(41)–C(40)	–176.2(2)
C(37)–C(36)–C(41)–C(40)	2.6(3)
C(47)–C(42)–C(43)–C(44)	–0.8(3)
C(57)–C(42)–C(43)–C(44)	179.6(2)
C(42)–C(43)–C(44)–C(45)	–2.2(3)
C(42)–C(43)–C(44)–Br(3)	175.80(16)
C(43)–C(44)–C(45)–C(46)	2.8(3)
Br(3)–C(44)–C(45)–C(46)	–175.22(17)
C(44)–C(45)–C(46)–C(47)	–0.4(3)
C(45)–C(46)–C(47)–C(48)	172.4(2)
C(45)–C(46)–C(47)–C(42)	–2.6(3)
C(43)–C(42)–C(47)–C(48)	–171.9(2)
C(57)–C(42)–C(47)–C(48)	7.8(3)
C(43)–C(42)–C(47)–C(46)	3.1(3)
C(57)–C(42)–C(47)–C(46)	–177.22(19)
C(46)–C(47)–C(48)–C(49)	–175.5(2)
C(42)–C(47)–C(48)–C(49)	–0.6(3)
C(47)–C(48)–C(49)–C(56)	–8.9(3)

C(47)–C(48)–C(49)–C(50)	163.5(2)
C(48)–C(49)–C(50)–C(55)	–119.5(2)
C(56)–C(49)–C(50)–C(55)	52.5(3)
C(48)–C(49)–C(50)–C(51)	53.1(3)
C(56)–C(49)–C(50)–C(51)	–134.9(2)
C(55)–C(50)–C(51)–C(52)	–0.7(3)
C(49)–C(50)–C(51)–C(52)	–173.3(2)
C(50)–C(51)–C(52)–C(53)	0.3(4)
C(51)–C(52)–C(53)–C(54)	0.3(4)
C(52)–C(53)–C(54)–C(55)	–0.6(4)
C(53)–C(54)–C(55)–C(50)	0.1(3)
C(51)–C(50)–C(55)–C(54)	0.5(3)
C(49)–C(50)–C(55)–C(54)	173.3(2)
C(48)–C(49)–C(56)–C(57)	11.5(3)
C(50)–C(49)–C(56)–C(57)	–160.5(2)
C(48)–C(49)–C(56)–C(67)	–161.8(2)
C(50)–C(49)–C(56)–C(67)	26.1(3)
C(49)–C(56)–C(57)–C(42)	–4.4(3)
C(67)–C(56)–C(57)–C(42)	169.10(18)
C(49)–C(56)–C(57)–C(58)	178.38(19)
C(67)–C(56)–C(57)–C(58)	–8.1(3)
C(43)–C(42)–C(57)–C(56)	174.4(2)
C(47)–C(42)–C(57)–C(56)	–5.2(3)
C(43)–C(42)–C(57)–C(58)	–8.3(3)
C(47)–C(42)–C(57)–C(58)	172.03(18)
C(56)–C(57)–C(58)–C(65)	–20.1(3)
C(42)–C(57)–C(58)–C(65)	162.61(18)
C(56)–C(57)–C(58)–C(59)	104.2(2)
C(42)–C(57)–C(58)–C(59)	–73.1(2)
C(57)–C(58)–C(59)–C(64)	103.9(2)
C(65)–C(58)–C(59)–C(64)	–129.5(2)
C(57)–C(58)–C(59)–C(60)	–76.0(2)
C(65)–C(58)–C(59)–C(60)	50.6(3)
C(64)–C(59)–C(60)–C(61)	–1.2(3)
C(58)–C(59)–C(60)–C(61)	178.66(19)
C(59)–C(60)–C(61)–C(62)	–0.6(3)
C(60)–C(61)–C(62)–C(63)	1.5(4)
C(61)–C(62)–C(63)–C(64)	–0.4(4)
C(60)–C(59)–C(64)–C(63)	2.2(3)
C(58)–C(59)–C(64)–C(63)	–177.6(2)
C(62)–C(63)–C(64)–C(59)	–1.5(4)
C(57)–C(58)–C(65)–C(66)	23.5(3)
C(59)–C(58)–C(65)–C(66)	–101.7(2)
C(57)–C(58)–C(65)–C(78)	–164.20(18)
C(59)–C(58)–C(65)–C(78)	70.7(2)
C(78)–C(65)–C(66)–C(69)	4.5(3)
C(58)–C(65)–C(66)–C(69)	176.60(19)
C(78)–C(65)–C(66)–C(67)	–170.17(19)
C(58)–C(65)–C(66)–C(67)	1.9(3)
C(57)–C(56)–C(67)–C(68)	–136.0(2)
C(49)–C(56)–C(67)–C(68)	37.5(3)
C(57)–C(56)–C(67)–C(66)	35.0(3)
C(49)–C(56)–C(67)–C(66)	–151.5(2)
C(65)–C(66)–C(67)–C(68)	139.1(2)
C(69)–C(66)–C(67)–C(68)	–35.5(3)
C(65)–C(66)–C(67)–C(56)	–31.6(3)
C(69)–C(66)–C(67)–C(56)	153.8(2)
C(65)–C(66)–C(69)–C(76)	–5.2(3)
C(67)–C(66)–C(69)–C(76)	169.3(2)
C(65)–C(66)–C(69)–C(70)	174.4(2)
C(67)–C(66)–C(69)–C(70)	–11.2(3)
C(76)–C(69)–C(70)–C(75)	–46.0(3)
C(66)–C(69)–C(70)–C(75)	134.4(2)
C(76)–C(69)–C(70)–C(71)	131.6(2)

C(66)–C(69)–C(70)–C(71)	–47.9(3)
C(75)–C(70)–C(71)–C(72)	–0.8(4)
C(69)–C(70)–C(71)–C(72)	–178.6(2)
C(70)–C(71)–C(72)–C(73)	0.4(4)
C(71)–C(72)–C(73)–C(74)	0.9(4)
C(72)–C(73)–C(74)–C(75)	–1.7(4)
C(73)–C(74)–C(75)–C(70)	1.3(4)
C(71)–C(70)–C(75)–C(74)	0.0(3)
C(69)–C(70)–C(75)–C(74)	177.8(2)
C(66)–C(69)–C(76)–C(77)	0.9(3)
C(70)–C(69)–C(76)–C(77)	–178.7(2)
C(69)–C(76)–C(77)–C(82)	–175.2(2)
C(69)–C(76)–C(77)–C(78)	3.9(3)
C(76)–C(77)–C(78)–C(79)	178.4(2)
C(82)–C(77)–C(78)–C(79)	–2.5(3)
C(76)–C(77)–C(78)–C(65)	–4.5(3)
C(82)–C(77)–C(78)–C(65)	174.58(19)
C(66)–C(65)–C(78)–C(79)	177.3(2)
C(58)–C(65)–C(78)–C(79)	4.9(3)
C(66)–C(65)–C(78)–C(77)	0.4(3)
C(58)–C(65)–C(78)–C(77)	–171.99(18)
C(77)–C(78)–C(79)–C(80)	2.8(3)
C(65)–C(78)–C(79)–C(80)	–174.1(2)
C(78)–C(79)–C(80)–C(81)	–0.6(3)
C(78)–C(79)–C(80)–Br(4)	179.27(16)
C(79)–C(80)–C(81)–C(82)	–2.0(3)
Br(4)–C(80)–C(81)–C(82)	178.12(17)
C(80)–C(81)–C(82)–C(77)	2.3(3)
C(76)–C(77)–C(82)–C(81)	179.0(2)
C(78)–C(77)–C(82)–C(81)	–0.1(3)

Symmetry transformations used to generate equivalent atoms:
